# Euglenatides, Potent Antiproliferative Cyclic Peptides Isolated from the Freshwater Photosynthetic Microalga *Euglena gracilis*

**DOI:** 10.1002/anie.202203175

**Published:** 2022-04-06

**Authors:** Mohammed Aldholmi, Rizwan Ahmad, Daniel Carretero‐Molina, Ignacio Pérez‐Victoria, Jesús Martín, Fernando Reyes, Olga Genilloud, Léa Gourbeyre, Thierry Gefflaut, Hanne Carlsson, Alexei Maklakov, Ellis O'Neill, Robert A. Field, Barrie Wilkinson, Maria O'Connell, A. Ganesan

**Affiliations:** ^1^ Natural Products and Alternative Medicine College of Clinical Pharmacy Imam Abdulrahman Bin Faisal University Dammam 31441 Saudi Arabia; ^2^ Fundación MEDINA Centro de Excelencia en Investigación de Medicamentos Innovadores en Andalucía Avenida del Conocimiento 34 18016 Armilla Granada Spain; ^3^ Université Clermont Auvergne Clermont Auvergne INP, CNRS, Institut Pascal 63000 Clermont-Ferrand France; ^4^ School of Biological Sciences University of East Anglia Norwich Research Park Norwich NR4 7TJ UK; ^5^ School of Chemistry University of Nottingham Nottingham NG7 2RD UK; ^6^ Manchester Institute of Biotechnology University of Manchester Manchester M1 7DN UK; ^7^ John Innes Centre Norwich Research Park Norwich NR4 7UH UK; ^8^ School of Pharmacy University of East Anglia Norwich Research Park Norwich NR4 7TJ UK

**Keywords:** Antiproliferative, Cyclic Peptides, Microalgae, Natural Products, Nematodes

## Abstract

By limiting the nitrogen source to glutamic acid, we isolated cyclic peptides from *Euglena gracilis* containing asparagine and non‐proteinogenic amino acids. Structure elucidation was accomplished through spectroscopic methods, mass spectrometry and chemical degradation. The euglenatides potently inhibit pathogenic fungi and cancer cell lines e.g., euglenatide B exhibiting IC_50_ values of 4.3 μM in *Aspergillus fumigatus* and 0.29 μM in MCF‐7 breast cancer cells. In an unprecedented convergence of non‐ribosomal peptide synthetase and polyketide synthase assembly‐line biosynthesis between unicellular species and the metazoan kingdom, euglenatides bear resemblance to nemamides from *Caenorhabditis elegans* and inhibited both producing organisms *E. gracilis* and *C. elegans*. By molecular network analysis, we detected over forty euglenatide‐like metabolites in *E. gracilis*, *E. sanguinea* and *E. mutabilis*, suggesting an important biological role for these natural products.

## Graphical abstract

Light and glutamic acid induced *E. gracilis* to produce euglenatides with antifungal and anticancer activity. Euglenatides were also detected in *E. sanguinea* and *E. mutabilis*. They resemble the nemamides isolated from *C. elegans*, inhibited both producing species, and represent uniquely convergent assembly‐line biosynthesis between unicellular organisms and metazoans.

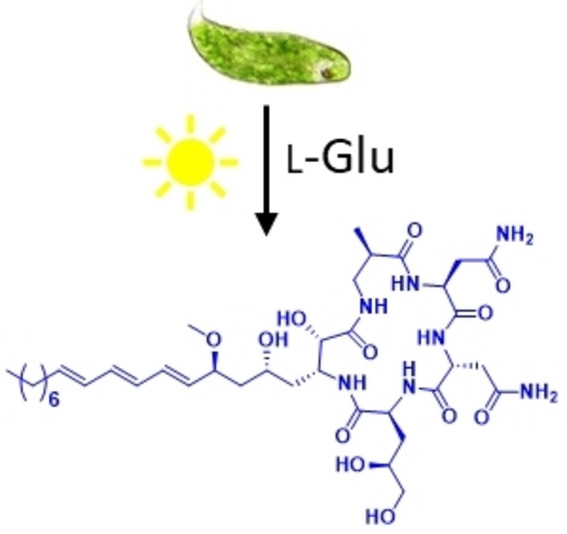

## Introduction

Microorganisms are a bountiful source of biologically active secondary metabolites, especially those with antimicrobial or cytotoxic properties.[^1^, ^2^] Indeed, the treatment of infectious disease and cancer relies heavily on natural products or their semisynthetic or fully synthetic analogues.^[3]^ Nevertheless, we have only scratched the surface of microbial metabolite diversity, as a tiny fraction of species are successfully isolated and cultured.[^4^, ^5^] Furthermore, even within this minority, the vast number of secondary metabolite biosynthetic gene clusters (BGCs) remain silent under artificial laboratory conditions.^[6]^ Many approaches are being studied for the activation of BGCs including the alteration of fermentation parameters, addition of small molecule elicitors, expression or deletion of transcriptional modulators and reconstitution in a heterologous host.[^7^, ^8^]

**Figure 1:**
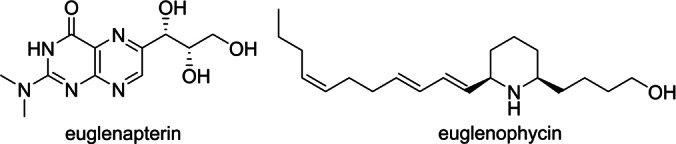
Previous secondary metabolites isolated from *Euglena*.

As a case in point, the common freshwater unicellular microalga *Euglena gracilis* is capable of plant‐like photoautotrophic growth with light as an energy source and carbon dioxide as the carbon source, animal‐like heterotrophic feeding with an external carbon source, or mixotrophically combining the two modes. *E. gracilis* is produced commercially as a food supplement,^[9]^ investigated as a potential biofuel source of lipids, carbohydrates and vitamins,^[10]^ and even grown with complete replacement of hydrogen by deuterium,^[11]^ or in outer space as a potential bioregenerative life support system.^[12]^ Despite such intensive scrutiny, the heterocycle euglenapterin (Figure 1) is the only secondary metabolite reported from *E. gracilis*.[^13^, ^14^] In fact, the only natural product of note from the entire *Euglena* genus (>250 species) is the cytotoxic alkaloid euglenophycin, isolated from *E. sanguinea* and detected in several other species but absent in *E. gracilis*.[^15^, ^16^] Nonetheless, partial genome sequencing of *E. gracilis* and transcriptome analysis identified a large number of enzymes involved in secondary metabolism.[^17^, ^18^] This included 19 non‐ribosomal peptide synthetases (NRPSs) and 14 polyketide synthases (PKSs), multidomain enzymes involved in assembly‐line biosynthesis,[^19^, ^20^] suggesting that *E. gracilis* is a potential source of hitherto undiscovered natural products.

## Results and Discussion

### Discovery of Euglenatides

**Figure 2:**
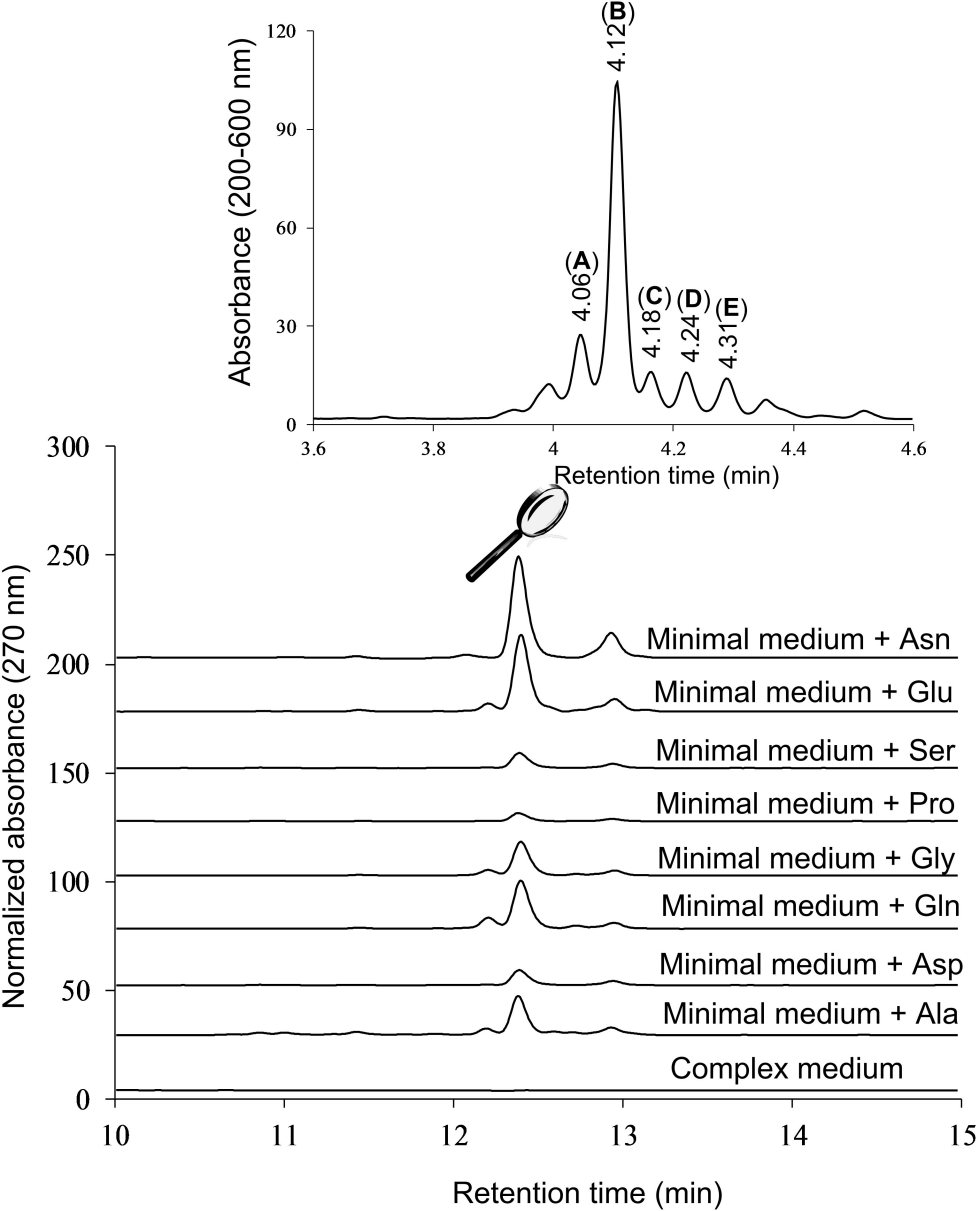
Overlay of HPLC traces at 270 nm from *E. gracilis* extracts cultured in complex or minimal media supplemented with 30 mM of individual amino acids. Under optimized HPLC conditions, the major induced peak split into multiple overlapping signals.

We cultured *Euglena gracilis* var. *saccharophila* Klebs (strain 1224/7A) under photosynthetic and heterotrophic conditions (media composition in Table S1, S2). Disappointingly, only common chlorophyll and xanthophyll photosynthetic pigments were perceived in extracts of either the supernatant or the algal biomass. The addition of small molecule epigenetic modulators, that we used to activate BGCs in *Aspergillus* fungi,^[21]^ was also to no avail. A breakthrough came about by considering the role of our nitrogen source. While the standard culture medium contains a complex mixture of amino acids arising from tryptone and yeast extract protein hydrolysates, Oda et al. reported that amino acids can either promote or inhibit growth in *E. gracilis*, with glutamic acid being the most effective nutrient.^[22]^ Consequently, we removed tryptone and yeast extract from our medium and instead used a minimal composition supplemented by glutamic acid. Gratifyingly, new metabolites were now observed in the biomass extract of *E. gracilis* grown photosynthetically, and the effect was replicated by several other amino acids, notably asparagine and glutamine (Figure 2). Time course experiments indicated a maximal metabolite production after ten days of cultivation in minimal medium with the addition of 30 mM glutamic acid (Figure S1). Under higher resolution in the HPLC, the main induced peak split into multiple closely overlapping signals (Figure 2). We named the compounds corresponding to the five major peaks as euglenatides A–E in order of chromatographic elution.

From UV/Vis absorption maxima at 260, 269 and 280 nm (Figure S2), we surmised the euglenatides contain a conjugated triene substructure.^[23]^ In negative mode electrospray mass spectrometry (ESI‐MS, Figure S3), the molecular ions and predicted molecular formulae did not correspond to any known natural products: euglenatide A, *m*/*z* 825.4612 [*M*−H]^−^ (calcd. for C_39_H_65_N_6_O_13_, 825.4615, Δ=−0.4 ppm); euglenatide B *m*/*z* 808.4464 [*M*−H]^−^ (calcd. for C_38_H_62_N_7_O_12_, 808.4462, Δ=0.3 ppm); euglenatide C *m*/*z* 822.4615 [*M*−H]^−^ (calcd. for C_39_H_64_N_7_O_12_, 822.4618, Δ=−0.4 ppm); euglenatide D *m*/*z* 809.4659 [*M*−H]^−^ (calcd. for C_39_H_65_N_6_O_12_, 809.4666, Δ=−0.9 ppm); euglenatide E *m*/*z* 792.4516 [*M*−H]^−^ (calcd. for C_38_H_62_N_7_O_11_, 792.4513, Δ=0.4 ppm). In positive mode ESI‐MS (Figure S4), in addition to the molecular ion, all euglenatides produced prominent fragments of [*M*−32]^+^ and [*M*−50]^+^ that we attributed to the loss of methanol (32 Da) or methanol+water (50 Da).

### Isolation and Structure Elucidation

We cultured *E. gracilis* in larger 18 L scale, with minimal medium+30 mM Glu, at ambient temperature under photosynthetic conditions (irradiation by daylight lamps, 2000 lumens). Following cell lysis and extraction, the residue of 1.7 g was initially purified by silica flash chromatography to give 187 mg of a mixture of euglenatides. Repeated cycles of semi‐preparative HPLC finally afforded 2.8 mg of pure euglenatide A, 5.7 mg of euglenatide B, 2.8 mg of euglenatide C, 2.3 mg of euglenatide D and 2.4 mg of euglenatide E. IR absorptions at 3347 cm^−1^ (NH) and 1651 cm^−1^ (CO) revealed the presence of amide carbonyl groups while the broad peak at 2873 cm^−1^ (CH) was characteristic of an aliphatic chain.

**Figure 3:**
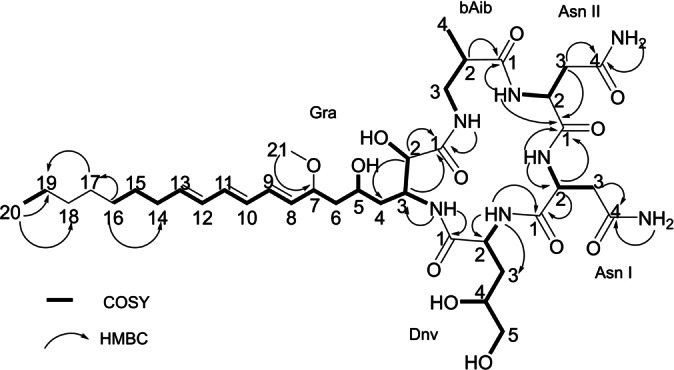
Key COSY and HMBC correlations used to establish the molecular connectivity of euglenatide B.

The molecular connectivity of euglenatides was established by extensive 1D and 2D‐NMR experiments (Figures S5–S40). For euglenatide B (Table S3), the presence of nine amide protons at *δ*_H_ 6.88 (1 H, s), 6.90 (1 H, d), 7.06 (1 H, s), 7.29 (1 H, s), 7.36 (1 H, s), 7.65 (1 H, d), 7.77 (1 H, d), 7.78 (1 H, s) and 9.02 (1 H, d) in the ^1^H NMR spectrum, and seven carbonyl carbon signals at *δ*_C_ 176.3, 173.4, 172.4, 172.3, 172.1, 171.1 and 169.6 implied a peptide backbone. In addition, overlapping signals at *δ*_H_ 1.25 in the ^1^H NMR spectrum and a cluster of methylene carbon signals at *δ*_C_ 28.5–31.2 in the HSQC spectrum, and a methyl group at *δ*_H_ 0.85 (3H, t) and *δ*_C_ 13.9 indicated the presence of a long aliphatic chain. The signals at *δ*_H_ 4.31, 4.38, 4.44 and 5.13 were diagnostic of four hydroxyls while the singlet at *δ*_H_ 3.13, accompanied by *δ*_C_ 55.5, indicated a methoxy group. A triene, as inferred from the UV/Vis spectrum, was confirmed by six proton signals at *δ*_H_ 5.51 (1 H, dd), 5.72 (1 H, dt), 6.07 (1 H, dd), 6.15 (1 H, dd), 6.20 (1 H, dd), 6.24 (1 H, dd), and six carbon signals at *δ*_C_ 135.1, 133.9, 132.8, 131.3, 130.3, 130.0. Analysis of ^1^H, ^13^C, and HSQC data, in addition to key COSY and HMBC correlations, unveiled the presence of two asparagine residues (Asn I and Asn II) and three non‐proteinogenic amino acids: β‐aminoisobutyric acid (βAib), 4,5‐dihydroxynorvaline (Dnv) and the novel β‐amino‐2,5‐dihydroxy‐7‐methoxy‐8,10,12‐eicosatrienoic acid residue that we named graciline (Gra) after its origin from *E. gracilis*. The sequence of amino acid residues was established by correlations of the NH protons to the amide carbonyls and CH_α_ and CH_β_ protons in the amino acid side chains in HMBC and COSY spectra, resulting in the complete 2D structure of euglenatide B (Figure 3). The allylic loss of methanol and water assisted by the conjugated triene is presumably a facile process, accounting for the resulting fragment ions observed in positive mode mass spectrometry.

**Figure 4:**
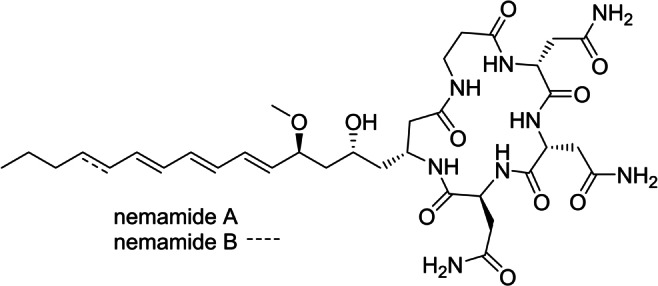
The structures of nemamides A and B.

The non‐proteinogenic amino acid βAib is rarely found within natural products, previously reported in only the guanidine phascolosomine from the sipunculid worm *Phascolion strombi*,^[24]^ and the lipopeptides YM‐170320 and fusaristatins from fungal *Candida* and *Fusarium* sp. respectively.[^25^, ^26^] To the best of our knowledge, euglenatide B is the first natural product identified to contain Dnv, although the *O*‐carbamoyl derivative is present in the antimicrobial *Streptomyces* polyoxin nucleoside peptides and GE81112 tetrapeptides.[^27^, ^28^] Meanwhile, to our surprise, the long chain C20 lipophilic β‐amino acid Gra bears a resemblance to the C16 amino acid found within the nemamides isolated by the Butcher group from the nematode *Caenorhabditis elegans* (Figure 4).^[29]^ The nemamides are the product of the multimodule megasynthases PKS‐1 and NRPS‐1 in *C. elegans*, and the first example of such assembly‐line biosynthesis in a metazoan. Furthermore, the presence of PKS‐1 and NRPS‐1 homologs in many nematode genomes implies an important physiological function for these peptides. It is remarkable that nemamides, the only known metazoan PKS‐NRPS metabolites, should possess structural similarity in the lipophilic region to euglenatides of microalgal origin. While the rest of the nemamide backbone contains three Asn residues, the euglenatides are more complex and contain additional stereocenters with one Asn replaced by Dnv and βAla substituted by the chiral βAib in euglenatide B.

The structures of euglenatides A, C, D and E were elucidated through their relationship to euglenatide B. In brief (Figure S41, Tables S4–S7), euglenatide A contained the same amino acids as euglenatide B, except that Asn II was replaced by a second Dnv residue. Euglenatides D and E were deoxy analogues of euglenatides A and B, respectively, without the C2‐hydroxyl group of the graciline residue. The NMR spectrum of euglenatide C was unique relative to the others, as it contained an extra methyl group (^1^H, *δ* 1.02 ppm; ^13^C, *δ* 19.5 ppm), and COSY/HMBC correlations assigned this to the replacement of Dnv by 4,5‐dihydroxynorleucine (Dnl).

Numerous attempts to obtain diffraction quality X‐ray crystals of euglenatide B were unsuccessful. The stereochemistry of Asn residues was instead determined through their conversion to aspartic acid by microscale acidic degradation. The hydrolysate was subjected to Marfey's analysis by chemical derivatization with *N*_α_‐(2,4‐dinitro‐5‐fluorophenyl)‐L‐valinamide (L‐FDVA).^[30]^ Both L‐FDVA‐L‐Asp (RT=20.66 min) and L‐FDVA‐D‐Asp (RT=25.52 min) were found, indicating one each of L‐Asn and D‐Asn in euglenatide B, whereas only the D amino acid was detected from euglenatide A (Figure S42).

Previously, the configurational assignment of βAib by Marfey analysis was complicated by difficult HPLC separation of the two diastereomers and required mathematical peak fitting by Gaussian deconvolution.^[31]^ By careful optimization of conditions including mobile and stationary phase, temperature, flow rate and isocratic solvent system, we devised a new protocol with sufficient chromatographic resolution: L‐FDVA‐L‐βAib, RT=59.47 min and L‐FDVA‐D‐βAib, RT=56.98 min (Figure S43). This confirmed that both euglenatides A and B contain the (*R*)‐enantiomer, identical in stereochemistry to naturally occurring βAib isolated from bacterial, fungal or plant sources. On the other hand, in humans, βAib is a catabolite of thymine and valine metabolism and both enantiomers are detected in plasma.^[32]^

Determination of the Dnv stereochemistry was challenging due to the lack of commercial standards and we employed a bi‐enzymatic recycling cascade synthesis from Ala involving pyruvate‐aldolase and L‐ or D‐α‐transaminase.^[33]^ This resulted in two scalemic samples: the L‐series contained L‐Ala (70 %)+L‐*syn*‐Dnv (26 %)+L‐*anti*‐Dnv (4 %) while the D‐series contained D‐Ala (25 %)+D‐*syn*‐Dnv (9 %)+D‐*anti*‐Dnv (66 %). Marfey analysis with these samples resulted in clear separation of the four Dnv diastereomers: L‐FDVA‐L‐*anti*‐Dnv, RT=17.03 min; L‐FDVA‐L‐*syn*‐Dnv, RT=32.38 min; L‐FDVA‐D‐*anti*‐Dnv, RT=24.92 min; L‐FDVA‐D‐*syn*‐Dnv, RT=29.90 min (Figure S44). Our hydrolysates were found to produce L‐FDVA‐L‐*anti*‐Dnv, and further confirmation came from enantiopure Boc‐L‐*anti*‐Dnv(4‐TBDMS)‐OEt, an intermediate in GE81112A total synthesis kindly provided by Dr. Armin Bauer at Sanofi‐Aventis.^[34]^ Global deprotection of this material to Dnv followed by Marfey's analysis gave an identical result to our sample. We note that of the four diastereomers of Dnv, only L‐*anti*‐Dnv or its *O*‐carbamoyl derivative are currently observed in natural products.

**Figure 5:**
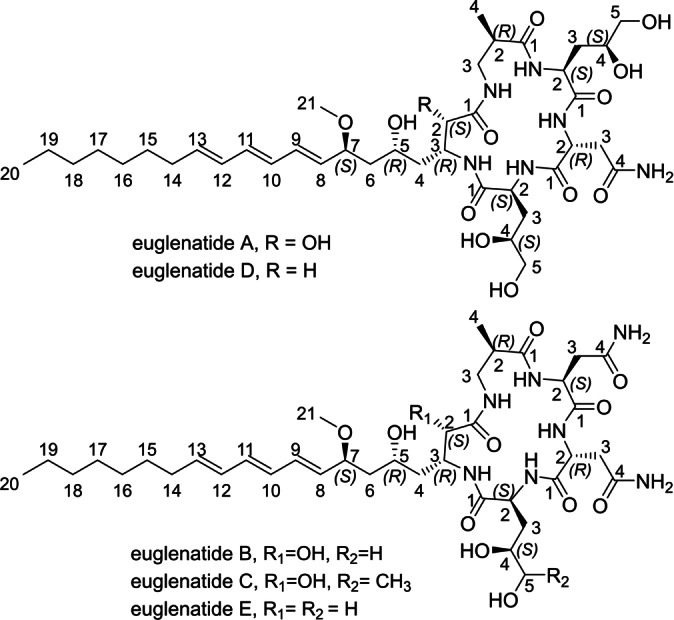
Structures of euglenatides A–E.

Neither the intact Gra residue nor simpler fragments were recognized from our acid hydrolysis, due to the lability of this complex amino acid under these conditions. The ^1^H and ^13^C NMR chemical shifts, coupling constants and NOESY correlations (Tables S8–S11) of 2‐deoxyGra in euglenatides D and E closely matched the data for nemamide A, as did the CD spectra (Figure S45). We conclude that 2‐deoxyGra is identical in relative and absolute stereochemistry with the nemamide side chain. The chirality of the additional C2 hydroxyl group in graciline was deduced from comparison of *J* values with nemamide A. Combining all the above information completed the structure elucidation of euglenatides A–E (Figure 5).

### Antiproliferative Activity of Euglenatides

The euglenatides were tested for antibacterial activity with methicillin‐sensitive *Staphylococcus aureus* (MSSA ATCC 29213), methicillin‐resistant *S. aureus* (MRSA MB5393) and *Escherichia coli* ATCC 25922 but were inactive at the highest tested concentration of 128 μg mL^−1^ (Figure S46). On the other hand, antifungal activity was observed against the yeast *Candida albicans* ATCC 64124. Euglenatides B, C and E had IC_50_ values of 13–14 μM, whereas euglenatide A (27 μM) and D (40 μM) were less active (Figure S47). Since this strain is ketoconazole resistant, it signifies the euglenatides are not cross‐resistant with the clinically important azole class of antifungal agents. All five euglenatides had a higher activity against the mold *Aspergillus fumigatus* ATCC 46645, with similar IC_50_ values of 4–9 μM. The lack of activity against bacteria suggested a specific target in eukaryotic cells absent in prokaryotes, and we screened the most abundant member, euglenatide B, in several human cancer cell lines. Potent antiproliferative activity was noted with respective IC_50_ values of 533, 773 and 292 nM against the THP‐1 acute monocytic leukemia, A‐549 lung adenocarcinoma and MCF‐7 breast cancer cell lines (Figure S48).

The Butcher group did not report bioassays with the worm nemamides, perhaps due to the paucity of material as only 0.07 mg of nemamide A and even less of nemamide B was isolated from 50 L of nematode culture. Instead, *C. elegans* mutants that do not produce nemamides were discerned to be defective in recovery from arrest of the L1 larval stage under starvation conditions. Based on the similarity of the lipophilic side chain between nemamides and euglenatides, we predict the former would possess antiproliferative properties against fungal and mammalian cancer cells.

### Effects on Producing Organisms

**Figure 6:**
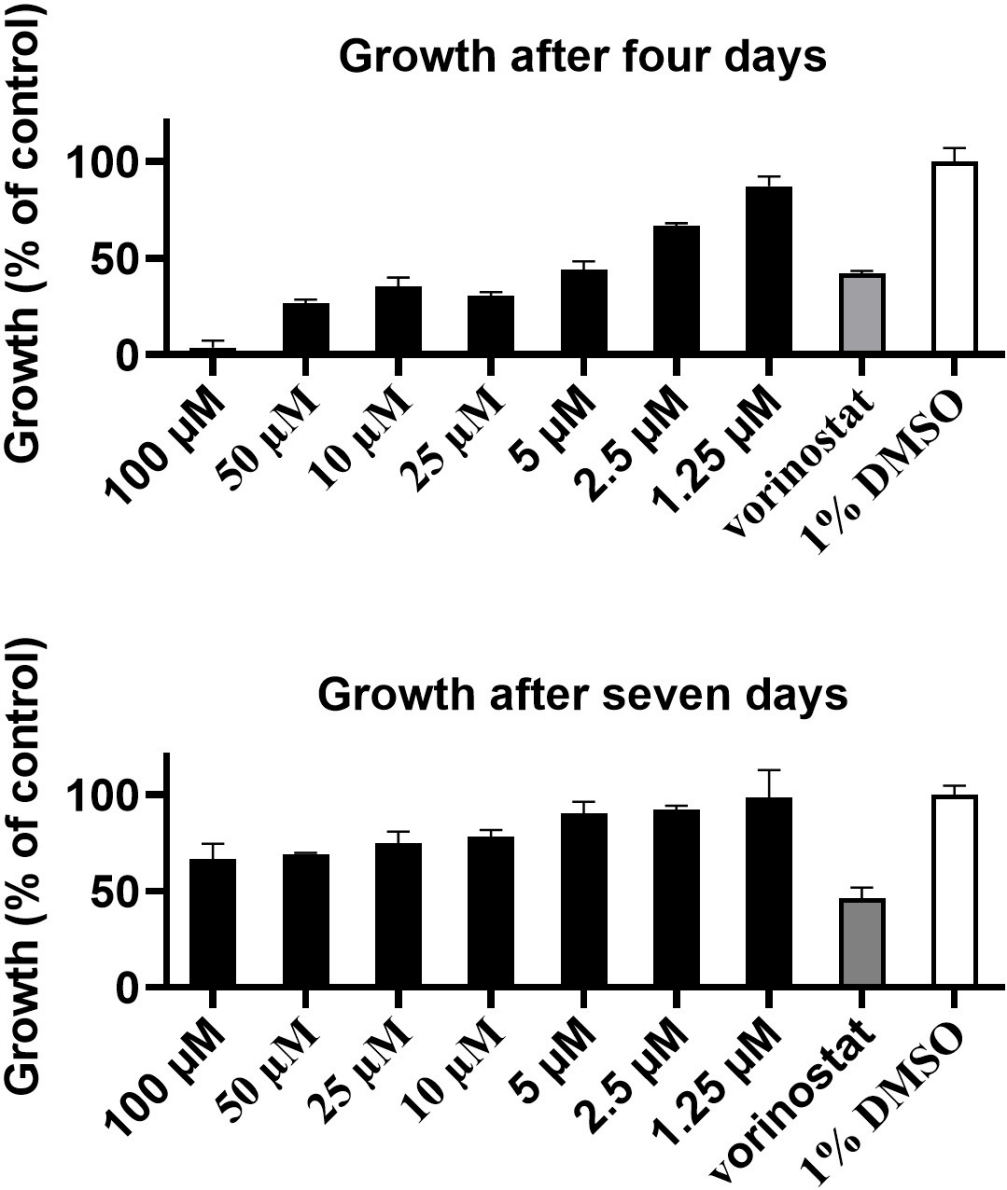
Algastatic effects of euglenatide B (1.25 μM to 100 μM) on the growth of *E. gracilis* after four and seven days of treatment. Vorinostat (1 mM) and DMSO (1 %) were used as positive and negative controls respectively. The data represent the average±standard error of three replicates.

**Figure 7:**
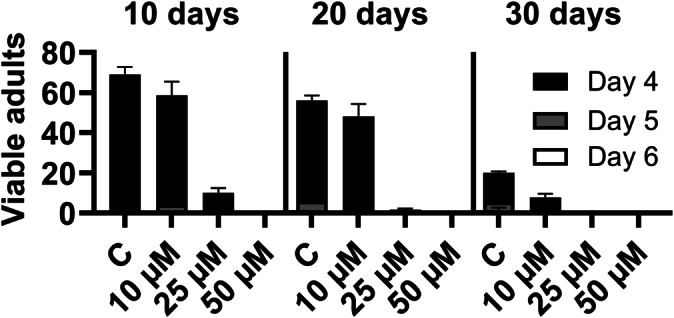
Inhibition of L1 *C. elegans* recovery by euglenatide B (10 μM to 50 μM, C=control) after starvation for 10, 20 or 30 days. The data represent three replicates with 100 eggs per treatment, based on the total number of sexually mature adults recovered by day four, five and six, respectively.

Given the biological activity against eukaryotes, we were curious if euglenatides would influence the producer *E. gracilis* itself. After four days of algal incubation with euglenatide B, growth was significantly inhibited (Figure 6). The effect appears to be cytostatic rather than cytotoxic, as the algae recovered after a longer period of seven days. The question then arises whether the nemamides have similar bioactivity against their nematode producer *C. elegans*. Although the worm nemamides are unavailable for testing, we indirectly addressed this issue by using our euglenatides. At concentrations of 10 and 25 μM, euglenatide B was strongly inhibitory to starvation recovery of the L1 larval stage of *C. elegans* (Figure 7).

It is tempting to speculate that the nemamides in *C. elegans* and the euglenatides in *E. gracilis* perform similar biological functions. While low concentrations might serve regulatory purposes and facilitate recovery from starvation conditions without negative effects on the producing organism, the external addition of higher amounts is apparently deleterious and overcomes mechanisms of self‐resistance.[^35^, ^36^] In our *Euglena* cultures, we did not detect euglenatides in the supernatant, implying that they are not secreted into the external environment. It is possible the natural products are sequestered in a specific cellular compartment to reduce self‐exposure and serve as antifeedants that discourage predators.

### A Common Biosynthetic Pathway in Euglena

We cultivated *E. sanguinea* and *E. mutabilis* strains under a variety of conditions and subjected extracts from cultures with good or excellent growth to metabolomic profiling by LC‐MS. The UV/Vis profile of the *E. sanguinea* extract was similar to that of *E. gracilis*, while the *E. mutabilis* extract did not have significant UV/Vis absorptions in the 200–600 nM range (Figures S49, S50). This suggests that both *E. gracilis* and *E. sanguinea* produce euglenatides with conjugated trienes, while they are either absent or in very low amounts in *E. mutabilis*.

**Figure 8:**
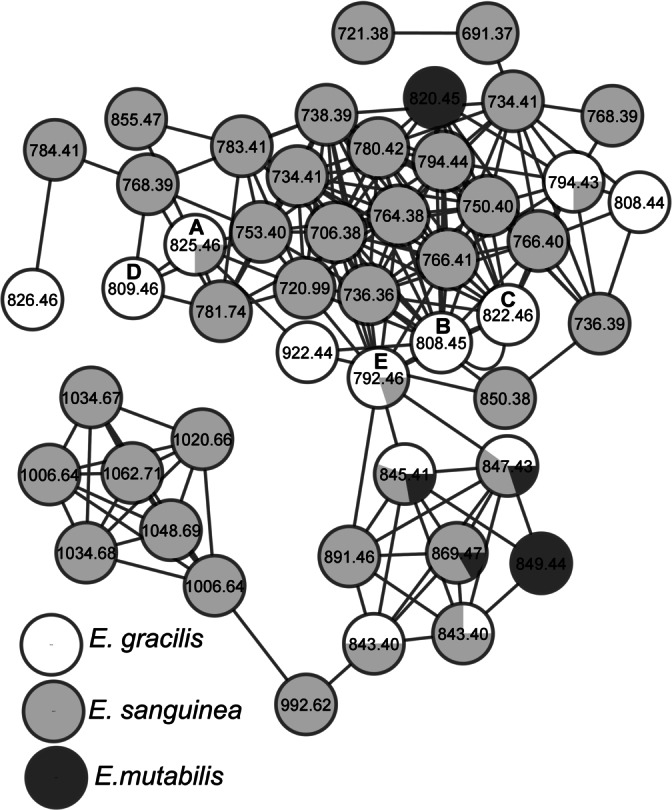
Molecular network analysis of euglenatides produced by *E. gracilis* (white circles), *E. sanguinea* (gray) and *E. mutabilis* (black). Each node represents the precursor mass of a single metabolite. The color of the nodes indicates the relative abundance of each metabolite between strains based on the MS ion intensities of precursors. The isolated euglenatides are represented by A, B, C, D and E respectively.

MS/MS spectra of *E. gracilis*, *E. sanguinea* and *E. mutabilis* extracts were obtained in negative ion mode and used to construct a molecular network of related metabolites (Figure 8).^[37]^ Each node in the molecular network is labelled with the precursor mass of the molecule deduced from the corresponding MS/MS spectrum. The constructed molecular network groups the structurally similar metabolites from three *Euglena* species in a cluster. The higher the similarity, the closer they are in the cluster. For example, precursor masses 808 and 822 represent the MS/MS spectra of euglenatides B and C which differ only by the addition of a methyl group, while precursor masses of 809 and 825 represent euglenatides A and D that differ by an additional hydroxyl group. Our network analysis indicates the presence of over 40 euglenatide‐like metabolites within these three species, including ubiquitous examples such as 845 and 847 and others such as 849 and 1006, detected only in *E. mutabilis* and *E. sanguinea*, respectively. The broad distribution, and the multitude of family members, point to an important function for euglenatides and we searched the available genomic *Euglena gracilis* data for homology with the nemamide BGC.^[38]^ However, due to the incomplete assembly, and the likely absence of gene clustering in *Euglena*, we did not identify a candidate BGC for euglenatide biosynthesis.

## Conclusion

We demonstrate that even a species like *Euglena gracilis*, subject to scientific study for centuries, harbors unusual and novel secondary metabolites. Ultimately, our successful unmasking of the euglenatides relied on the traditional “one strain, many compounds (OSMAC)” approach of altering fermentation conditions.^[39]^ Specifically, culture in minimal media with glutamic acid as the sole nitrogen source served to activate a normally quiescent biosynthetic pathway. The resulting euglenatides contain L‐ and D‐asparagine, as well as (*R*)‐β‐aminoisobutyric acid, a rare constituent in peptide natural products. In addition, the euglenatides are the first peptide natural products disclosed to incorporate 4,5‐dihydroxynorvaline, 4,5‐dihdroxynorleucine and the novel C20 polyketide derived β‐amino acid graciline with a conjugated triene and two oxygenated stereocenters in the side chain. Interestingly, the *E. sanguinea* metabolite euglenophycin (Figure 1) also contains 20 carbons, and it is plausible that it shares a common C20 polyunsaturated fatty acid precursor with the euglenatides.

Euglenatides exhibited significant antiproliferative activity against *Candida* and *Aspergillus*, the two major pathogenic fungal genera, and a panel of human cancer cells. By molecular network analysis, we detected over 40 euglenatide‐like metabolites in three *Euglena* species, implying a widespread occurrence within the genus. The euglenatides share a structural similarity to the nemamides, the only known metazoan natural product of NRPS‐PKS origin and inhibited the growth of both producing organisms *E. gracilis* and *C. elegans*. The astonishing structural congruence between protist and animal kingdom secondary metabolites is unique in chemical ecology. The ability to harvest multimg quantities of euglenatides from *E. gracilis* will expedite further research into the biological ramifications of these cyclic peptides.

## Conflict of interest

The authors declare no conflict of interest.

1

## Supporting information

As a service to our authors and readers, this journal provides supporting information supplied by the authors. Such materials are peer reviewed and may be re‐organized for online delivery, but are not copy‐edited or typeset. Technical support issues arising from supporting information (other than missing files) should be addressed to the authors.

Supporting Information

## Data Availability

The data that support the findings of this study are available from the corresponding author upon reasonable request.
