# Supplementary material for: Euglenatides, Potent Antiproliferative Cyclic Peptides Isolated from the Freshwater Photosynthetic Microalga Euglena gracilis
Source: Angew Chem Int Ed Engl. 2022 Apr 6;61(23):e202203175. doi: 10.1002/anie.202203175 (PMC9321709; doi:10.1002/anie.202203175)
Supplement: Supplementary file 1 — Supporting Information [file ANIE-61-0-s001.pdf]

Angewandte Chemie
Eine Zeitschrift der Gesellschaft Deutscher Chemiker

Supporting Information

Euglenatides, Potent Antiproliferative Cyclic Peptides Isolated from the Freshwater Photosynthetic Microalga *Euglena gracilis*

M. Aldholmi, R. Ahmad, D. Carretero-Molina, I. Pérez-Victoria, J. Martín, F. Reyes, O. Genilloud, L. Gourbeyre, T. Gefflaut, H. Carlsson, A. Maklakov, E. O'Neill, R. A. Field, B. Wilkinson, M. O'Connell, A. Ganesan**

Supporting information

Table of Contents

Index of tables 2
Index of figures 3
Materials and methods 6
1. General methods and materials 6
2. Inocula preparation 6
3. Small-scale cultivation and extraction 7
4. HPLC analysis 7
5. LC-PDA-MS analysis 8
6. High resolution MS 8
7. Determination of the optimum fermentation period 9
8. Large-scale cultivation, isolation and purification of euglenatides 9
9. Marfey’s analysis 14
10. Synthesis of Dnv stereoisomers using recycling enzyme cascades 15
11. Antimicrobial assays 16
12. Cancer cell growth inhibition assay 19
13. *E. gracilis* growth inhibition assay 20
14. *C.elegans* starvation recovery assay 20
15. Euglenatide-related metabolites and molecular networking analysis 21
References 65

Index of tables

Table S1 EG (*E. gracilis*) medium. 48

Table S2 JM (Jaworski's medium). 48

Table S3 ^{1}H and ^{13}C NMR data (500 and 125 MHz, DMSO-d_6) of euglenatide B. 49

Table S4 ^{1}H and ^{13}C NMR data (500 and 125 MHz, DMSO-d_6) of euglenatide E. 50

Table S5 ^{1}H and ^{13}C NMR data (500 and 125 MHz, DMSO-d_6) for euglenatide C. 51

Table S6 ^{1}H and ^{13}C NMR data (500 and 125 MHz, DMSO-d_6) of euglenatide A. 52

Table S7 ^{1}H and ^{13}C NMR data (500 and 125 MHz, DMSO-d_6) for euglenatide D. 53

Table S8 Comparison of ^{1}H NMR chemical shifts in the triene side chain between euglenatides and nemamide A. 57

Table S9 Comparison of ^{13}C NMR chemical shifts in the triene side chain between euglenatides and nemamide A. 58

Table S10 Comparison of NOESY correlations and *J* coupling between protons at C2, N2, N18 and C17 in the triene side chain of euglenatides and reported data for nemamide A. 58

Table S11 Comparison of NOESY correlations and *J* coupling between protons at C17, C18, C20, C21, C22 and C24 in the triene side chain of euglenatides and reported data for nemamide A. 59

Index of figures

Figure S1 Comparative UHPLC traces (at 270 nm; 100 × 2.1mm, 2.6μm Accucore C18 column; 0.5 mL/min flow rate; 20-100% acetonitrile over 10 min) of *E. gracilis* extracts from day 1 to day 13 of inoculation. 23

Figure S2 UV-Vis profile of *E. gracilis* metabolites showing absorbance maxima at 260, 269 and 280 nm. 23

Figure S3 Negative ion mode ESI-MS spectra of euglenatides A, B, C, D and E showing $[M-H]^-$ and $[M+Cl]^-$. 24

Figure S4 Positive ion mode MS spectra of euglenatides A, B, C, D and E showing $[M+Na]^+$ and two characteristic protonated product ions corresponding to a neutral loss of 50 Da (loss of H_2O and MeOH) and 32 Da (loss of MeOH). 25

Figure S5 Semi-preparative HPLC trace (at 270 nm) of *E. gracilis* metabolites with euglenatides A-E indicated. 26

Figure S6 LC-UV-MS chromatograms, IR and MS spectra of euglenatide A. 27

Figure S7 ^{1}H NMR spectrum of euglenatide A in DMSO-d_6. 28

Figure S8 ^{13}C NMR spectrum of euglenatide A in DMSO-d_6. 28

Figure S9 COSY NMR spectrum of euglenatide A in DMSO-d_6. 29

Figure S10 HSQC NMR spectrum of euglenatide A in DMSO-d_6. 29

Figure S11 HMBC NMR spectrum of euglenatide A in DMSO-d_6. 30

Figure S12 NOESY NMR spectrum of euglenatide A in DMSO-d_6. 30

Figure S13 LC-UV-MS chromatograms, IR and MS spectra of euglenatide B. 31

Figure S14 ^{1}H NMR spectrum of euglenatide B in DMSO-d_6. 32

Figure S15 ^{13}C NMR spectrum of euglenatide B in DMSO-d_6. 32

Figure S16 COSY NMR spectrum of euglenatide B in DMSO-d_6. 33

Figure S17 HSQC NMR spectrum of euglenatide B in DMSO-d_6. 33

Figure S18 HMBC NMR spectrum of euglenatide B in DMSO-d_6. 34

Figure S19 NOESY NMR spectrum of euglenatide B in DMSO-d_6. 34

Figure S20 LC-UV-MS chromatograms, IR and MS spectra of euglenatide C. 35

Figure S21 ^{1}H NMR spectrum of euglenatide C in DMSO-d_6. 36

Figure S22 ^{13}C NMR spectrum of euglenatide C in DMSO-d_6. 36

Figure S23 COSY NMR spectrum of euglenatide C in DMSO-d_6. 37

Figure S24 HSQC NMR spectrum of euglenatide C in DMSO-d_6. 37

Figure S25 HMBC NMR spectrum of euglenatide C in DMSO-d_6. 38

Figure S26 NOESY NMR spectrum of euglenatide C in DMSO-d_6. 38

Figure S27 LC-UV-MS chromatograms, IR and MS spectra of euglenatide D. 39

Figure S28 ^{1}H NMR spectrum of euglenatide D in DMSO-d_6. 40

Figure S29 ^{13}C NMR spectrum of euglenatide D in DMSO-d_6. 40

Figure S30 COSY NMR spectrum of euglenatide D in DMSO-d_6. 41

Figure S31 HSQC NMR spectrum of euglenatide D in DMSO-d_6. 41

Figure S32 HMBC NMR spectrum of euglenatide D in DMSO-d_6. 42

Figure S33 NOESY NMR spectrum of euglenatide D in DMSO-d_6. 42

Figure S34 LC-UV-MS chromatograms, IR and MS spectra of euglenatide E. 43

Figure S35 ^{1}H NMR spectrum of euglenatide E in DMSO-d_6. 44

Figure S36 ^{13}C NMR spectrum of euglenatide E in DMSO-d_6. 44

Figure S37 COSY NMR spectrum of euglenatide E in DMSO-d_6. 45

Figure S38 HSQC NMR spectrum of euglenatide E in DMSO-d_6. 45

Figure S39 HMBC NMR spectrum of euglenatide E in DMSO-d_6. 46

Figure S40 NOESY NMR spectrum of euglenatide E in DMSO-d_6. …………………..46

Figure S41 Key COSY and HMBC correlations used to establish the molecular connectivity of euglenatides, A, C, D and E. 47

Figure S42 Top: Reaction of Asp with Marfey's reagent. Bottom: extracted negative ion chromatograms of the *m/z* 412 in derivatised Asp standards and euglenatides A and B hydrolysates. 54

Figure S43 Top: Reaction of βAib with Marfey's reagent. Bottom: extracted negative ion chromatograms of the *m/z* 382 in derivatised βAib standards and euglenatides A and B hydrolysates. 55

Figure S44 Top: hydrolysis of the protected Dnv derivative and reaction with Marfey's reagent. Bottom: extracted ion chromatograms and spectra of the *m/z* 428 in derivatised Dnv standards and euglenatides A and B hydrolysates. 56

Figure S45 Comparison of CD spectra between euglenatide E and those reported for nemamide A and triene diastereomers.[9] 60

Figure S46 Dose–response curves of bacterial strains (MSSA, MRSA and *E. coli*) incubated with euglenatides A, B, C, D and E. 61

Figure S47 Dose–response curves and corresponding IC_{50} values of *C. albicans* and *A. fumigatus* incubated with euglenatides A, B, C, D and E. 62

Figure S48 Dose–response curves of THP-1, MCF-7 and A-549 cell lines incubated with euglenatide B or vorinostat (positive control). Data points represent the average ± standard error of three replicates. 63

Figure S49 Comparative total absorbance (TAC, 200-600 nm) of *E sanguinea* cultivated in EG:JM medium without peptide extracts + 60 mM Asn, EG:JM medium and EG:JM medium without peptide extracts + 30 mM Asn + 30 mM Glu. 64

Figure S50 Comparative total absorbance (200-600 nm) and total ion chromatograms of *E. mutabilis* cultivated in EG:JM medium. 64

Materials and methods

1. General methods and materials

Unless stated otherwise, all chemicals and solvents were purchased from Sigma-Aldrich, Alfa Aesar or Fisher Scientific. All solvents were of HPLC grade or equivalent. Commercial media and media components were purchased from Sigma-Aldrich, Alfa Aesar, Fisher Scientific or Formedium. Unless stated otherwise, the pH of media was adjusted to 4.8 with 1 N HCl and 1 N KOH prior to autoclaving.

UV spectra were acquired on an SPD-M20A photodiode array detector attached to a Shimadzu Prominence/Nexera UHPLC. NMR spectra were recorded on a Bruker Avance III spectrometer (500 and 125 MHz for ^{1}H and ^{13}C NMR, respectively) equipped with a 1.7-mm TCI MicroCryoProbe. Chemical shifts were reported in ppm using the signals of the residual solvent as internal reference (δH 2.50 and δC 39.5 ppm for DMSO-d_6). Optical rotations were acquired on a Jasco P-2000 polarimeter (JASCO Corporation, Tokyo, Japan). IR spectra were measured with a JASCO FT/IR-4100 spectrometer (JASCO Corporation) equipped with a PIKE MIRacle™ (JASCO Corporation) single reflection ATR accessory.

2. Inocula preparation

Euglena gracilis var. *saccharophila* Klebs (strain 1224/7A) was obtained from the Culture Collection of Algae and Protozoa (CCAP) (https://www.ccap.ac.uk) and treated with antibiotics to produce axenic cultures using the protocol recommended by CCAP.[1] Cultures were cultivated in EG:JM (*Euglena gracilis* medium (Table S1) plus Jaworski's Medium (Table S2), replacing ''Lab- Lemco'' with Tryptone)[2] and incubated in a light chamber at 22 °C on a 14 h light /10 h dark cycle with a light intensity of 100 μmol. photons. m^{-2} s^{-1}. After four days of incubation, *E. gracilis* cells were harvested by centrifugation at 2000 x g and 10 °C for 5 min and suspended in

EG:JM medium. The absorbance at 740 nm was adjusted on a CLARIOstar microplate reader to 0.1 (≈ 4 g of wet cells per litre) using EG:JM medium as a diluent and blank. Cells were harvested by centrifugation at 2000 x g and 10 ^{o}C for 5 min, washed three times with sterile Milli-Q water to remove any trace components of EG:JM medium, and suspended in the medium used for cultivation. This suspension was used to inoculate the production media to obtain approximately 0.2 g of wet cells per litre (1:20 dilution).

3. Small-scale cultivation and extraction

50-mL tubes each containing 10 mL of the medium were inoculated with *Euglena* cells suspension to obtain approximately 0.2 g of wet cells per litre (1:20 dilution). Cultures were cultivated at ambient temperature 22-25 ^{o}C under daylight lamps (2000 lumens). After six days, cultures were centrifuged at 3000 x g and 4 ^{o}C for 20 min, and cells were vortexed in 10 mL 90% MeOH. Cell debris was removed by centrifugation at 3000 x g and 4 ^{o}C for 10 min. After centrifugation, the solvents were evaporated, and the residue of each extract was then dissolved in 1 mL MeOH and passed through SOLA HRP SPE cartridges (Thermo Fisher Scientific) and a 0.2 µm PTFE filter (Whatman, Sigma-Aldrich). For cultivation in the dark, the media were supplemented with 15 g/ L glucose (Glc) and incubated at 30 ^{o}C and shaken at 200 rpm in the dark for four days after inoculation with *E. gracilis* cells.

4. HPLC analysis

HPLC analysis was performed on an Agilent 1200 series system (Agilent Technologies) and an Agilent C18 column (4.6 x 150mm, 5µm). The following gradient was used (acetonitrile versus 0.1% formic acid in water, at 1.3mL/min and 40°C): 0 min (20% acetonitrile), 20 min (100% acetonitrile), 24 min (100% acetonitrile), 25 min

(20% acetonitrile), 30 min (20% acetonitrile), and the detection was set at 210, 270, 290 and 450 nm.

5. LC-PDA-MS analysis

LC-PDA-MS data were acquired on a Shimadzu Nexera UHPLC attached to a Shimadzu ion-trap-ToF (IT-ToF) mass spectrometer. A 5-μL aliquot of each sample was injected onto a Phenomenex Kinetex EVO-C18 column (100 × 2.1mm, 2.6μm). The following gradient was used (acetonitrile versus 0.1% formic acid in water, at 0.5 mL/min and 40°C): 0 min (20% acetonitrile), 10 min (100% acetonitrile), 12 min (100% acetonitrile), 12.1 (20% acetonitrile), 16.1 min (20% acetonitrile). The instrument collected UV/visible spectra from 200-600 nm and MS spectra from *m/z* 200-2000. MS also collected MS/MS (fragmentation) spectra from the most abundant ions using collision-induced dissociation energy of 50% and a precursor ion width of 3 Da. Spray chamber conditions were 250°C curved desorbation line (CDL), 300°C heat block, 1.5 $L.min^{-1}$ nebuliser gas, and drying gas "on". Sodium trifluoroacetate was used for mass calibration according to the manufacturer's instructions. The LC and MS data were analysed using LabSolutions (Shimadzu), profiling solutions (Shimadzu) and ACD/Spectrus Processor (Advanced Chemistry Development).

6. High resolution MS

For the accurate mass measurement, high-resolution mass spectra were acquired on a Synapt G2-Si mass spectrometer (Waters) operated in negative ion mode with a scan time of 1.0 second in the mass range of *m/z* 50 to 1200. An aliquot of 7 μL of each sample was injected onto an Acquity UPLC® BEH C18 column, 1.7 μm, 1x100 mm (Waters) maintained at 45 °C and eluted with mobile phases A (water + 0.1% formic acid) and B (acetonitrile + 0.1% formic acid) at a flow rate of 80 μL/min. The following gradient was used: 0-1 min (15% B), 11 min (99% B), 11.1 min (15 % B), 15

min (15 % B). The following parameters were used: capillary voltage = 2.5 kV; cone voltage = 40 V; source temperature = 130 °C; desolvation temperature = 350 °C; source offset 80 V. Leu-enkephalin peptide was used as a lock mass (*m/z* = 554.2620) measured every 30 seconds during the run.

7. Determination of the optimum fermentation period

A one-litre flask containing 500 mL of synthetic medium + 30 mM Glu was inoculated with *Euglena* cells to obtain approximately 0.2 g of wet cells per litre (1:20 dilution). An aliquot of 10 mL was taken every day for 13 days, starting 24 h after inoculation, and centrifuged at 3000 x g and 4 ^{o}C for 20 min. The cells were harvested and extracted with 90% MeOH. The solvent was evaporated, and the residue of the extract was stored in a freezer at - 20 ^{o}C until HPLC analysis. Samples were prepared immediately before analysis by adding 1 mL MeOH and passing the solutions through SOLA HRP SPE cartridges (Thermo Fisher Scientific) and a 0.2 µm PTFE filter (Whatman, Sigma-Aldrich).

8. Large-scale cultivation, isolation and purification of euglenatides

Synthetic medium + 30 mM Glu (18 L) was inoculated with *Euglena* cells to obtain approximately 0.2 g of wet cells per litre (1:20 dilution). The culture was cultivated at ambient temperature 22-25 ^{o}C under daylight lamps (2000 lumens). After ten days, the culture was centrifuged at 3130 x g and 4 ^{o}C for 20 min, and the pellet (53 g) was extracted with 1 L 90% MeOH on a magnetic stirrer. After an hour, the aqueous MeOH extract was partitioned with an equal volume of hexane to remove lipids and pigments. The aqueous MeOH layer was centrifuged to remove the debris and then evaporated to obtain the residue (1.68 g). This amount was dissolved in 50 mL MeOH along with 15 g of C18 powder, and the solvent was evaporated. The resulting powder was halved

into two columns packed with 65 g reversed-phase resin, previously equilibrated with 20% acetonitrile before a frit was added on the top. The extract loaded on the column was fractionated on an automated flash-chromatography system (CombiFlash Rf, Teledyne Isco) using 18 mL/min flow rate and a linear gradient from 20% to 70% acetonitrile in water (in 40 min) followed by a ramp to 100% acetonitrile in 4 min before washing the column for 16 min.

Fractions containing the target compounds were concentrated to dryness on a centrifugal evaporator to give a residue amount of 186.7 mg. This amount was dissolved in MeOH, and the compounds were purified on a semi-preparative C18 column attached to Gilson HPLC system to afford 2.8 mg of euglenatide A, 5.7 mg of euglenatide B, 2.8 mg of euglenatide C, 2.3 mg of euglenatide D and 2.4 mg of euglenatide E. Isocratic elution with 34% acetonitrile in water and 3 mL/min flow rate were used, and the elution was monitored by UV detection at 210 and 270 nm. The purity of the isolated compounds was confirmed by injecting an aliquot in an Agilent 1100 LC-DAD-MS system using a Zorbax SB-C8 column (2.1 × 30 mm, 5 μm, flow rate 0.3 mL/min, 40 °C) with a linear gradient from 10% B to 100% B in 6 min followed by 2 min hold at 100% B before returning to10% B and equilibrating the system for 2 min. Solvent A was 10% acetonitrile in water, and solvent B was 90% acetonitrile in water, both supplemented with 1.3 mM trifluoroacetic acid and ammonium formate.

*Euglenatide A (**1**)*: 2.8 mg; light yellow amorphous powder; $[\alpha]_D^{25}$ -24.2 (c 0.76, MeOH); UV λ_{max} (MeOH) 260, 269, 280 nm; IR ν_{max} 3347, 3286, 2873, 2811, 1651 and 1607 cm^{-1}; *m/z* 825.4612 $[M-H]^-$ (calcd. for $C_{39}H_{65}N_6O_{13}$, 825.4615, Δ= -0.4 ppm); ^1H NMR (DMSO-d_6, 500 MHz) 8.85, (d, J = 5.7 Hz, 1H), 7.79 (s, 1H), 7.78 (s, 1H), 7.73 (d, J = 7.7 Hz, 1H), 7.29 (s, 1H), 7.08, (brd , J = 12.7 Hz, 1H), 6.92 (d, J = 9.6 Hz, 1H), 6.24 (dd, J = 14.0, 10.6 Hz, 1H), 6.20 (dd, J = 14.6, 10.3 Hz, 1H), 6.15 (dd, J = 14.0,

10.6 Hz, 1H), 6.07 (dd, *J* = 14.0, 10.6 Hz, 1H), 5.72 (dt , *J* = 14.1, 7.1 Hz, 1H), 5.51(dd, *J* = 14.7, 7.9 Hz, 1H), 5.17 (brd, *J* = 7.5 Hz, 1H), 4.55 (m, 1H), 4.45 (m, 1H), 4.44 (m, 1H), 4.42 (m, 1H), 4.34 (m, 1H), 4.31 (m, 1H), 4.28 (m, 1H), 3.92 (m, 1H), 3.87 (d, *J* = 9.6, 7.5 Hz, 1H), 3.81 (m, 1H), 3.73 (m, 1H), 3.47 (m, 1H), 3.42 (m, 1H), 3.41 (m, 1H), 3.29 (m, 2H), 3.27 (m, 1H), 3.22 (m, 2H), 3.13 (s, 3H), 3.06 (brd, *J* = 12.9 Hz, 1H), 2.94 (dd, *J* = 16.8, 4.8 Hz, 1H), 2.69 (m, 1H), 2.57 (dd, *J* = 16.5, 3.4 Hz, 1H), 2.06 (q , *J* = 7 Hz, 2H) 1.89 (m, 2H), 1.83 (m, 1H), 1.71 (m, 1H), 1.69 (m, 1H), 1.49 (m, 1H), 1.34 (m, 2H), 1.31 (br dd, *J* = 15.0, 4.8 Hz, 1H), 1.25 (m, 6H), 1.24 (m, 2H), 1.20 (m, 1H), 1.07 (d, *J* = 6.9 Hz, 3H), 0.85 (t , *J* = 6.9 Hz, 3H); ^{13}C NMR (DMSO-d_6, 125 MHz) 176.2, 173.6, 172.6, 172.3, 171.2, 170.7, 135.2, 134, 133, 131.4, 130.4, 130.1, 77.9, 73, 68.1, 67.6, 66.3 (2C), 63.1, 55.6, 51.6, 50.6, 49.3, 49.2, 43.9, 41.8, 40.4, 37.7, 35.8, 34.5, 32.3, 32.2, 31.3, 28.8, 28.6 (2C), 22.1, 17, 14.

*Euglenatide B (**2**)*: 5.7 mg; light yellow amorphous powder; $[\alpha]_D^{25}$ -27.7 (c 0.97, MeOH); UV λ_{max} (MeOH) 260, 269, 280 nm; IR ν_{max} 3347, 3286, 2873, 2811, 1651 and 1607 cm^{-1}; *m/z* 808.4464 [M-H]$^-$ (calcd. for $C_{38}H_{62}N_7O_{12}$, 808.4462, Δ= 0.3 ppm); ^{1}H NMR (DMSO-d_6, 500 MHz) 9.02 (d, *J* = 6.5 Hz, 1H), 7.78 (s, 1H), 7.77 (d, *J* = 7.9 Hz, 1H), 7.65 (d, *J* = 7.8 Hz, 1H), 7.36 (s, 1H), 7.29 (s, 1H), 7.06 (s , 1H), 6.90 (d, *J* = 9.6 Hz, 1H), 6.88 (s , 1H), 6.24 (dd, *J* = 14.5, 10.4 Hz, 1H), 6.20 (dd, *J* = 14.6, 10.3 Hz, 1H), 6.15 (dd, *J* = 14.6, 10.3 Hz, 1H), 6.07 (dd, *J* = 15.1, 10.4 Hz, 1H), 5.72 (dt , *J* = 15.1, 7.1 Hz, 1H), 5.51(dd, *J* = 14.6, 7.6 Hz, 1H), 5.13 (brd, *J* = 7.7 Hz, 1H), 4.45 (m, 1H), 4.44 (brd, *J* = 4.7 Hz, 1H), 4.38 (m, 1H), 4.32 (m, 1H), 4.31 (m, 1H), 4.06 (m, 1H), 3.92 (m, 1H), 3.86 (d, *J* = 9.6, 7.7 Hz, 1H), 3.73 (m, 1H), 3.47 (m, 1H), 3.43 (m, 1H), 3.32 (m, 1H), 3.27 (m, 1H), 3.26 (m, 1H), 3.13 (s, 3H), 3.03 (brd, *J* = 13 Hz, 1H), 2.94 (dd, *J* = 16.8, 4.8 Hz, 1H), 2.86, (dd, *J* = 15.7, 4.0 Hz, 1H), 2.66 (m, 1H), 2.57 (dd, *J* = 16.9, 3.7 Hz, 1H), 2.47, (dd, *J* = 15.9, 4.7 Hz, 1H), 2.06 (q , *J* = 7 Hz, 2H), 1.86 (m,

1H), 1.71 (m, 1H), 1.69 (m, 1H), 1.49 (m, 1H), 1.35 (m, 2H), 1.30 (br dd, *J* = 14.5, 5.1 Hz, 1H), 1.25 (m, 6H), 1.24 (m, 2H), 1.20 (m, 1H), 1.04 (d, *J* = 7.1 Hz, 3H), 0.85 (t , *J* = 6.8 Hz, 3H); ^{13}C NMR (DMSO-d_6, 125 MHz) 176.3, 173.4, 172.4, 172.3, 172.1, 171.1, 169.6, 135.1, 133.9, 132.8, 131.3, 130.3, 130, 77.8, 72.9, 67.5, 66.3, 63, 55.5, 51.6, 50.3, 49.3, 49.1, 43.8, 41.7, 40.3, 37.5, 35.8, 35, 34.3, 32.1, 31.2, 28.7, 28.5 (2C), 22, 16.8, 13.9.

*Euglenatide C (**3**)*: 2.8 mg; light yellow amorphous powder; $[\alpha]_D^{25}$ -22.1 (c 0.59, MeOH); UV λ_{max} (MeOH) 260, 269, 280 nm; IR v_{max} 3347, 3286, 2873, 2811, 1651 and 1607 cm^{-1}; *m/z* 822.4615 [M-H]$^-$ (calcd. for $C_{39}H_{64}N_7O_{12}$, 822.4618, Δ= -0.4 ppm); ^{1}H NMR (DMSO-d_6, 500 MHz) 9.02 (d, *J* = 6.3 Hz, 1H), 7.79 (s, 1H), 7.76 (d, *J* = 8.5 Hz, 1H), 7.65 (d, *J* = 7.8 Hz, 1H), 7.36 (s, 1H), 7.29 (s, 1H), 7.06 (s , 1H), 6.90 (d, *J* = 9.6 Hz, 1H), 6.88 (s , 1H), 6.24 (dd, *J* = 14.5, 10.4 Hz, 1H), 6.20 (dd, *J* = 14.6, 10.3 Hz, 1H), 6.15 (dd, *J* = 14.6, 10.3 Hz, 1H), 6.07 (dd, *J* = 15.1, 10.4 Hz, 1H), 5.72 (dt , *J* = 15.1, 7.1 Hz, 1H), 5.51(dd, *J* = 14.6, 7.6 Hz, 1H), 5.13 (brd, *J* = 7.7 Hz, 1H), 4.45 (m, 1H), 4.32 (m, 1H), 4.31 (m, 1H), 4.27 (brd, *J* = 5.9 Hz, 1H), 4.18 (brd, *J* = 4.9 Hz, 1H), 4.06 (m, 1H), 3.92 (m, 1H), 3.86 (d, *J* = 9.6, 7.7 Hz, 1H), 3.73 (m, 1H), 3.47 (m, 1H), 3.39 (m, 1H), 3.27 (m, 1H), 3.18 (m, 1H), 3.13 (s, 3H), 3.03 (brd, *J* = 11.2 Hz, 1H), 2.94 (dd, *J* = 16.9, 5 Hz, 1H), 2.86, (dd, *J* = 15.4, 3.9 Hz, 1H), 2.66 (m, 1H), 2.57 (dd, *J* = 17, 3.6 Hz, 1H), 2.47, (dd, *J* = 15.5, 4 Hz, 1H), 2.06 (q , *J* = 7.1 Hz, 2H), 1.86 (m, 1H), 1.71 (m, 1H), 1.69 (m, 1H), 1.49 (m, 1H), 1.35 (m, 2H), 1.30 (br dd, *J* = 14.5, 5.2 Hz, 1H), 1.25 (m, 6H), 1.24 (m, 2H), 1.20 (m, 1H), 1.02 (t, *J* = 6.2 Hz, 6H), 0.85 (t , *J* = 6.8 Hz, 3H); ^{13}C NMR (DMSO-d_6, 125 MHz) 176.2, 173.4, 172.5, 172.3, 172.1, 171.1, 169.5, 135.1, 133.9, 132.8, 131.3, 130.3, 130, 77.8, 72.9, 70.6, 69.9, 63, 55.5, 51.6, 50.5, 49.3, 49.1, 43.8, 41.7, 40.3, 37.5, 35.8, 35, 34.2, 32.1, 31.2, 28.7, 28.5 (2C), 22, 19.5, 16.9, 13.9.

*Euglenatide D (**4**)*: 2.3 mg; light yellow amorphous powder; $[\alpha]_D^{25}$ -11.2 (c 0.88, MeOH); UV λ_{max} (MeOH) 260, 269, 280 nm; IR ν_{max} 3347, 3286, 2873, 2811, 1651 and 1607 cm^{-1}; *m/z* 809.4659 $[M-H]^-$ (calcd. for $C_{39}H_{65}N_6O_{12}$, 809.4666, Δ= -0.9 ppm); ^{1}H NMR (DMSO-d_6, 500 MHz) 8.85, (d, *J* = 5.3 Hz, 1H), 7.93 (s, 1H), 7.87 (d, *J* = 8.2 Hz, 1H), 7.71 (d, *J* = 7.5 Hz, 1H), 7.43 (s, 1H), 6.93 (overlap, 1H), 6.92 (overlap, 1H), 6.24 (dd, *J* = 14.5, 10.5 Hz, 1H), 6.20 (dd, *J* = 14.5, 10.5 Hz, 1H), 6.15 (dd, *J* = 14.7, 10.6 Hz, 1H), 6.07 (dd, *J* = 15.0, 10.5 Hz, 1H), 5.72 (dt , *J* = 15.1, 7.7 Hz, 1H), 5.51(dd, *J* = 14.7, 7.7 Hz, 1H), 4.57 (m, 1H), 4.55 (m, 1H), 4.45 (m, 1H), 4.44 (m, 1H), 4.42 (m, 2H), 4.34 (m, 1H), 4.26 (m, 1H), 4.08 (m, *J* = 9.6, 6.5, 3.3 Hz, 1H), 3.85 (m, 1H), 3.75 (m, 1H), 3.51 (m, 1H), 3.42 (m, 1H), 3.41 (m, 1H), 3.29 (m, 2H), 3.24 (m, 1H), 3.22 (m, 2H), 3.13 (s, 3H), 2.99 (dd, *J* = 16.9, 4.15 Hz, 1H), 2.92 (brd, *J* = 12.4 Hz, 1H), 2.66 (m, 1H), 2.58 (dd, *J* = 16.7, 3.2 Hz, 1H), 2.06 (q , *J* = 7 Hz, 2H) 1.89 (m, 2H), 1.85 (m, 1H), 1.69 (m, 1H), 1.49 (m, 1H), 1.35 (m, 2H), 1.32 (m, 1H), 1.30 (m, 1H), 1.26 (m, 2H), 1.25 (m, 4H), 1.23 (m, 2H), 1.07 (brd, *J* = 7.1 Hz, 3H), 0.85 (t , *J* = 6.8 Hz, 3H); ^{13}C NMR (DMSO-d_6, 125 MHz) 175.9, 174.1, 172.3, 171.2, 170.7, 170.4, 135.4, 133.8, 133.1, 131.5, 130.4, 130, 77.8, 68, 67.7, 66.3 (2C), 63.4, 55.7, 51.6, 50.4, 49.4, 45.2, 44.2, 43.6, 41.9, 38.2, 35.6, 34.2, 32.2 (2C), 31.3, 29, 28.8, 28.6, 22.1, 17.4, 14.

*Euglenatide E (**5**)*: 2.4 mg; light yellow amorphous powder; $[\alpha]_D^{25}$ -14.7 (c 0.65, MeOH); UV λ_{max} (MeOH) 260, 269, 280 nm; IR ν_{max} 3347, 3286, 2873, 2811, 1651 and 1607 cm^{-1}; *m/z* 792.4516 $[M-H]^-$ (calcd. for $C_{38}H_{62}N_7O_{11}$, 792.4513, Δ= 0.4 ppm); ^{1}H NMR (DMSO-d_6, 500 MHz) 8.96 (d, *J* = 6.5 Hz, 1H), 7.91 (s, 1H), 7.87 (d, *J* = 7.9 Hz, 1H), 7.64 (d, *J* = 7.8 Hz, 1H), 7.39 (s, 1H), 7.36 (s, 1H), 6.94 (overlap, 1H), 6.92 (overlap, 1H), 6.88 (s , 1H), 6.24 (dd, *J* = 14.5, 10.3 Hz, 1H), 6.19 (dd, *J* = 14.6, 10.2 Hz, 1H), 6.15 (dd, *J* = 14.5, 10.3 Hz, 1H), 6.07 (dd, *J* = 15.1, 10.4 Hz, 1H), 5.72 (dt , *J* = 15.1, 7.1 Hz, 1H), 5.51(dd, *J* = 14.6, 7.6 Hz, 1H), 4.55 (m, 1H), 4.47 (brd, *J* = 4.7 Hz,

1H), 4.42 (m, 1H), 4.41 (m, 1H), 4.30 (m, 1H), 4.11 (m, 1H), 3.75 (m, 1H), 3.51 (m, 1H), 3.43 (m, 1H), 3.32 (m, 1H), 3.26 (m, 1H), 3.24 (m, 1H), 3.13 (s, 3H), 3.01 (dd, *J* = 16.8, 5 Hz, 1H), 2.93 (brd, *J* = 13 Hz, 1H), 2.87 (dd, *J* = 15.7, 4.0 Hz, 1H), 2.64 (m, 1H), 2.57 (dd, *J* = 16.9, 3.7 Hz, 1H), 2.47, (dd, *J* = 15.9, 4.7 Hz, 1H), 2.16 (dd, *J* = 13.8, 3.3 Hz, 1H), 2.06 (q , *J* = 7 Hz, 2H), 1.87 (m, 1H), 1.69 (m, 1H), 1.48 (m, 1H), 1.35 (m, 2H), 1.32 (m, 1H), 1.30 (m, 1H), 1.27 (m, 2H), 1.25 (m, 6H), 1.02 (d, *J* = 7.1 Hz, 3H), 0.85 (t , *J* = 6.8 Hz, 3H); ^{13}C NMR (DMSO-d_6, 125 MHz) 176.1, 173.9, 172.3, 172.2, 171.1, 170.4, 169.7, 135.3, 133.8, 133, 131.5, 130.3, 129.9, 77.8, 67.6, 66.3, 63.3, 55.6, 51.7, 50.3, 49.4, 45.2, 44.2, 43.6, 41.7, 40.9, 38, 35.6, 35, 34.2, 32.2, 31.3, 29, 28.8, 28.5, 22.1, 17.1, 14.

9. Marfey's analysis

To determine the configurations of the two asparagine amino acids, euglenatides A and B were hydrolysed in sealed vials by dissolving 0.5 mg of each in 1 mL of 6 N HCl and heating at 110 °C for 16 h. The hydrolysates were evaporated to dryness in a centrifugal evaporator, and the residues were dissolved in 50 µL of Milli-Q water. Stock solutions of the amino acid standards (50 mM, L-Asp and D-Asp, Sigma-Aldrich) were prepared in Milli-Q water. The hydrolysates and standard amino acids (50 µL) were treated with 20 µL of 1 M $NaHCO_3$ and 150 µL of 1-fluoro-2,4-dinitrophenyl-5-L-valine-amide (L-FDVA, Marfey's reagent; 1% w/v in acetone) and heated at 40 °C for 1 h. Reactions were quenched with 20 µL of 1 N HCl, and an aliquot (10 µL) was diluted with acetonitrile (40 µL) and analysed by an Agilent 1100 LC-DAD-MS system (UV detection at 340 nm and ESI-MS detection in positive and negative modes). The following gradient was used (solvent A versus solvent B) on a Waters X-Bridge C18 column (4.6 × 150 mm, 5 µm) maintained at 40 °C, 0.0 min (10% B), 50.0 min (27% B), 54.0 min (100% B), 60.0 min (100% B), 61.0 min (10% B), 70.0 min (10% B), at a

flow of 1 mL/min. Solvent A was 10% acetonitrile in water, and solvent B was 90% acetonitrile in water, both supplemented with 1.3 mM trifluoroacetic acid and ammonium formate.

L-βAib and D-βAib were purchased from Sigma-Aldrich and Marfey derivatives prepared similarly to the Asp standards above. The LC-PDA-MS analysis was performed on a Shimadzu 2020 single quadrupole LC-PDA-MS system (UV/visible detection range from 200 to 600 nm and ESI-MS detection in positive and negative modes) using an Accucore C18 column (2.1 × 100 mm, 2.6 μm) maintained at 50 °C. An isocratic solvent system of MeOH/water (25/75) with 0.1% formic acid was used at a flow of 0.350 mL/min over 70 min. A derivative of L-*anti*-4,5-dihydroxynorvalinate derivative was kindly provided by Dr. Armin Bauer at Sanofi-Aventis, and mixtures of L-*syn*-Dnv, L-*anti*-Dnv, D-*syn*-Dnv and D-*anti*-Dnv were obtained by enzymatic synthesis (next section). These samples were hydrolysed in a sealed vial with 6 N HCl (1 mg/mL) at 110 °C for 16 h, and the hydrolysate was evaporated to dryness in a centrifugal evaporator. Marfey analysis was performed as described above with these standards and euglenatide hydrolysates. The LC-PDA-MS analysis was performed on a Shimadzu Prominence/Nexera IT-ToF LC-PDA-MS system (UV/visible detection range from 200 to 600 nm and ESI-MS detection in positive and negative modes) using an Accucore C18 column (2.1 × 100 mm, 2.6 μm) maintained at 30 °C. The following elution system was used (MeOH versus 0.1% formic acid in water, at 0.200 mL/min): 0.0-34.0 min (30% MeOH), 35.0 min (100% MeOH), 40.0 min (100% MeOH), 42.0 (30% MeOH), 50.0 min (30% MeOH).

10. Synthesis of Dnv stereoisomers using recycling enzyme cascades

To a mixture containing 0.5 M pyruvic acid (80 μL, 0.04 mmol), 0.5 M L- or D-Ala (400 μL, 0.2 mmol), 0.1 M $MgCl_2$ (40 μL, 4 μmol), 10 mM PLP (40 μL, 0.4 μmol), and H_2O

(2.7 mL) adjusted to pH 7.8 with 0.1 M NaOH, were added 1 $mg.mL^{-1}$ aldolase (250 µL, Uniprot Id. A0A081HJP9) and L-α-transaminase (TA) (6 mg, Uniprot Id. G0VQA2) or D- α-TA (1.5 mg, Uniprot Id. E6LHY8). A solution of 0.5 M glycolaldehyde (480 µL, 0.24 mmol) was then added over 3 h using a syringe pump. The reaction mixture was stirred at 20 °C for 4 h (with D-α-TA) or 24 h (with L-α-TA). The mixture was poured on a column of Dowex® 50WX8 (H+ form, 1 mL). The column was washed with H_2O (5 mL) and then eluted with 1 M NH_3 (10 mL). The ninhydrin positive fractions were pooled and concentrated under reduced pressure. The residue was analysed by ^1H NMR to determine the relative amounts of isomers of Dnv and residual Ala. Product configuration was assigned by comparison with spectroscopic data published for L-*syn* or *anti*-Dnv. [10–12] The following results were obtained for the L- or D-series:

L-Series: L-Ala (70%) + L-*syn*-Dnv (26%) + L-*anti*-Dnv (4%)

D-Series: D-Ala (25%) + D-*syn*-Dnv (10%) + D-*anti*-Dnv (65%)

11. Antimicrobial assays

Euglenatides A-E were evaluated for their antimicrobial activity against bacteria (methicillin-sensitive *S. aureus* (MSSA ATCC 29213), methicillin-resistant *S. aureus* (MRSA MB5393) and *E. coli* ATCC 25922), yeast (*C. albicans* ATCC 64124), and mould (*A. fumigatus* ATCC 46645) following previously described methods.[3–5] Each peptide was serially diluted in DMSO with a dilution factor of 2 to provide ten concentrations for all assays, and the final concentrations of each peptide were ranging from 128 to 0.25 µg/mL. Results were analysed using Microsoft Excel and GraphPad Prism 8.0 software.

To assess the antibacterial activity, thawed stock inocula suspensions from cryovials of each microorganism (MSSA ATCC 29213, MRSA MB5393 and *E. coli* ATCC 25922) were streaked onto LBA (Luria-Bertani agar plates, 40 g/L) and

incubated overnight at 37 °C. Isolated colonies of each microorganism were inoculated into Luria-Bertani broth medium (LB, 25 g/L) and incubated overnight (MSSA and MRSA) or for two hours (*E.coli*) at 37 °C with shaking at 220 rpm. After incubation for the specified periods, cultures were diluted in Miller Hinton II medium to attain assay inocula of approximately 5-6 × 10^5 CFU/mL (MSSA and *E. coli*) and 1.1 × 10^6 CFU/mL (MRSA). In 96-well microplates, 90 µL/well of the diluted inoculum were mixed with 1.6 µL/well of each concentration of each compound and 8.4 µL/well of Miller Hinton II medium. Aztreonam 20-0.078125 µg/mL was used as a positive control with *E. coli* and vancomycin 32-0.25 µg/mL with MSSA and MRSA. Absorbance was measured at 612 nm with a multimode plate reader (EnVision Perkin Elmer) at T_0 (zero time) immediately before incubation at 37 °C for 20 h. After this period, the assay plates were agitated using a DPC Micromix-5 and the absorbance was measured at T_f (final time). The following equation was used to calculate growth inhibition: Growth (% control) = 100 × {[(T_f Absorbance of treated culture - T_0 Absorbance of treated culture) - (T_f Absorbance of broth medium - T_0 Absorbance of broth medium)]/ [(T_f Absorbance of untreated culture - T_0 Absorbance of untreated culture) - (T_f Absorbance of broth medium - T_0 Absorbance of broth medium)]}.

To evaluate the activity against yeast, Sabouraud Dextrose Agar (SDA) plates were inoculated with frozen stocks of *C. albicans* ATCC 64124 and incubated at 35 °C. After 24 h, a few colonies were harvested from the plates and suspended in RPMI liquid medium prepared from RPMI-1640 medium (20.8 g of RPMI powder in 1.8 L) supplemented with 13.4 g of yeast nitrogen base, 72 mL of 50% glucose and 80 mL of 1 M HEPES. The liquid medium was filtered after adjusting the volume to 2 L with Milli-Q water. The optical density of C. *albicans* suspension was adjusted to 0.25 at 660 nm using the liquid medium as a diluent and blank. This inoculum was diluted 1:10

and reserved on ice until used for inoculation. In 96-well microplates, 90 µL/well of the diluted inoculum was mixed with 1.6 µL/well of each concentration of each compound and 8.4 µL/well of the liquid medium. Amphotericin B was used as a positive control. Absorbance was measured at 612 nm with a multimode plate reader (EnVision Perkin Elmer) at T_0 (zero time) immediately before incubation at 37 °C for 20 h. After this period, the assay plates were agitated using a DPC Micromix-5, and the absorbance was measured at T_f (final time). Growth inhibition was calculated using the equation mentioned above.

To test the antifungal activity against *A. fumigatus*, PDA plates were flooded with Tween saline (0.025% v/v of Tween 80 and 8 g/L NaCl), and colonies were harvested in RPMI liquid medium by gently scraping the surface of the agar with a sterile spatula to prepare a conidial suspension. This suspension was filtered through sterile chiffon, and the concentration was determined by counting the conidia in a Neubauer chamber. The inoculum was approximately 2.5×10^4 CFU/mL. Resazurin stock solution of 0.02 g in 100 mL was prepared from resazurin sodium salt (R7017, Sigma Aldrich) in Milli-Q water, sterilised by filtration and used as an indicator of eukaryotic cell viability (0.002% final concentration). Resazurin is a blue oxidation-reduction dye that is itself weakly fluorescent until it is irreversibly reduced to the pink-coloured and highly red-fluorescent resorufin.[4] In 96-well microplates, 90 µL/well of the inoculum were mixed with 1.6 µL/well of each concentration of each compound and 8.4 µL/well of the liquid medium. Amphotericin B and rifampicin were used as positive and negative controls, respectively. The plates were incubated at 37 °C for 25-30 h without agitation. After incubation, fluorescence was recorded on a multimode plate reader (EnVision Perkin Elmer) using wavelength settings for resorufin (excitation 570 nm, emission 600 nm). Growth inhibition was calculated using the equation mentioned

above but using the fluorescence instead of absorbance. Wells with 0.002% resazurin in broth medium were used as blanks.

12. Cancer cell growth inhibition assay

Three cancer cell lines (from ECACC) were cultured in RPMI 1640 medium (A549 and THP-1 cell lines) or DMEM (MCF-7 cell line) medium supplemented with 10% fetal calf serum, 2 mM L-glutamine, 100 U/mL penicillin and 100 µg/mL streptomycin (Invitrogen). Cells (3×10^4 /100 µL for THP-1 and 6×10^3 /100 µL for A549 and MCF-7) were seeded in 96-well plates and treated immediately (THP-1) or after 22 h (A549 and MCF-7) with 1 µL of DMSO (vehicle control) or 1 µL of the compound in DMSO at different concentrations in triplicate, and plates were incubated at 37 °C and 5% CO_2. Cell viability was assessed by CellTiter 96 Aqueous One Solution Cell Proliferation Assay (Promega) following the manufacturer's instructions. The assay contains MTS (3-(4,5-dimethylthiazol-2-yl)-5-(3-carboxymethoxyphenyl)-2-(4-sulfophenyl)-2H-tetrazolium) reagent, which is a colourimetric method that measures mitochondrial metabolic activity. After 72 h of incubation, MTS assay reagent (10 µL) was added to the cells, and plates were incubated further for 1.5 h for A549 and MCF-7 and 4 h for THP-1. Following this, absorbance was measured at 490 nm using a Polarstar Optima microplate reader (BMG Labtech). Wells with only culture medium and MTS solution were used to determine the background, and cells treated with 1% DMSO were used as the negative controls. Cell viability of treated cells was calculated relative to the negative controls after background correction of all wells. IC_{50} values were calculated using GraphPad Prism 8.0 software.

13. *E. gracilis* growth inhibition assay

E. gracilis cells were harvested from four-day-old culture by centrifugation at 2000 x g and 10 ^{o}C for 5 min and suspended in EG:JM or synthetic medium + 30 mM Asn. The absorbance at 740 nm was adjusted on a CLARIOstar microplate reader to 0.1 (≈ 4 g of wet cells per litre) using EG:JM or synthetic medium + 30 mM Asn as a diluent and blank. This inoculum was diluted 1:20 (≈ 0.2 g of wet cells per litre) and used to inoculate a 96-well microtiter plate. Each well contained 99 µL of the diluted inoculum and 1 µL of DMSO (vehicle control) or 1 µL of the compound in DMSO at two-fold serial dilutions with the highest concentration at 100 µM and the lowest at 1.25 µM. The plate was incubated at ambient temperature 22-25 ^{o}C under daylight lamps (2000 lumens), and the absorbance was measured at 740 nm after four and seven days of incubation. Wells with only growth medium were used for background correction, cells treated with 1% DMSO were used as negative controls, and cells treated with 1 mM of vorinostat were used as positive controls. Three replicates per condition were performed, and the percentage of growth inhibition was calculated relative to the negative controls after background correction of all wells. Results were analysed using Microsoft Excel and GraphPad Prism 8.0 software.

14. *C.elegans* starvation recovery assay

Starvation recovery was examined using the nematode *Caenorhabditis elegans* N2 Bristol strain obtained from the Caenorhabditis Genetics Centre (CGC). We kept eggs in 0.5ml aliquots of liquid media (S-buffer: 5.85 g NaCl, 1.123 g K_2HPO_4, 5.926 g KH_2PO_4 per 1 l dH_2O) without any nutrients, hence starving and arresting development of the nematodes at the L1 larvae stage. The liquid media was supplemented with either 10 µM, 25 µM or 50 µM of euglenatide B diluted in DMSO to a final concentration of 1% DMSO in the S-buffer, and a DMSO control with no euglenatide was also

included. The starvation vials where kept at 20 °C and inverted twice daily for aeration. We ran three separate starvation experiments when worms were starved either for 10 days, 20 days or 30 days. We used three replicate tubes containing 100 eggs each per treatment (i.e. euglenatide concentration) for each starvation experiment. After starvation we transferred the worms onto standard Nematode Growth Medium (NGM) plates seeded with *Escherichia coli* OP50 as a food source. We counted the number of viable reproductive adults after four, five and six days on food. For statistical analyses, we used the total number of sexually mature adults recovered on plates by day six. The presence of euglenatide in the media reduced the number of viable adults with increasing concentration of euglenatide in 10-day starvation (Kruskal-Wallis test: $\chi^2 = 9.8$, d.f. = 3, $p = 0.02$); 20-day starvation (Kruskal-Wallis test: $\chi^2 = 9.4$, d.f. = 3, $p = 0.02$); and 30-day starvation (Kruskal-Wallis test: $\chi^2 = 10.6$, d.f. = 3, $p = 0.014$).

15. Euglenatide-related metabolites and molecular networking analysis

E. gracilis, E. sanguinea and *E. mutabilis* were cultivated in the following media: EG:JM without peptide extracts + 30 mM Glu; EG:JM without peptide extracts + 30 mM Asn; EG:JM without peptide extracts + 60 mM Asn; EG:JM without peptide extracts + 60 mM Glu; EG:JM without peptide extracts + 30 mM Glu + 30 mM Asn; EG:JM medium. Inocula preparation, cultivation and extraction were performed as described above. Extracts were analysed by a Shimadzu LC-PDA-MS as described above. The LC and MS data were processed using ACD/Spectrus, and the MS/MS spectra were converted to an mzXML file format using LabSolutions software (Shimadzu). GNPS spectral library[6] was searched for similar molecules using the converted files of MS/MS spectra (GNPS; https://gnps.ucsd.edu) and used to construct a molecular network of euglenatide-related metabolites. The following GNPS

parameters were used:[7] Parent Mass Tolerance = 1 Da, Min Pairs Cos = 0.6, Min Matched Peaks = 3, Network TopK = 15, MSCluster = ON, Minimum Peak Intensity = 25, Filter Precursor Window = OFF, Filter Library = OFF, and Filter peaks in 50 Da Window = OFF. The molecular network was exported from GNPS and analysed using the visualisation software Cytoscape.[8] The GNPS job can be found on the following linkhttps://gnps.ucsd.edu/ProteoSAFe/status.jsp?task=8c575ac25fb8422aaffd212eae5c1b15. The MASSIVE dataset is available to the public with the following name and number: Euglenatides from *Euglena* species MSV000088616.

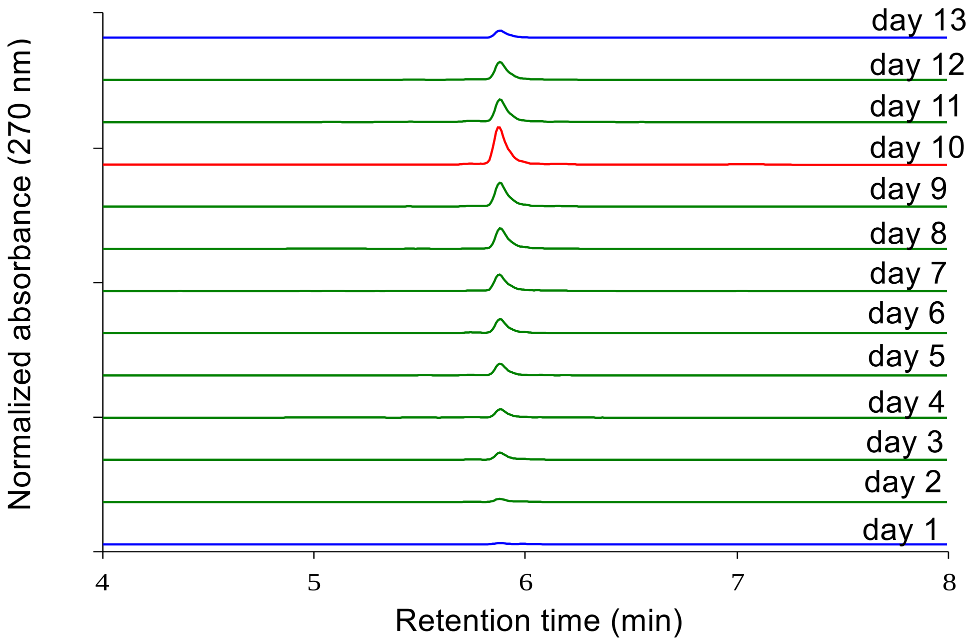

Figure S1 Comparative UHPLC traces (at 270 nm; 100 × 2.1mm, 2.6µm Accucore C18 column; 0.5 mL/min flow rate; 20-100% acetonitrile over 10 min) of *E. gracilis* extracts from day 1 to day 13 of inoculation.

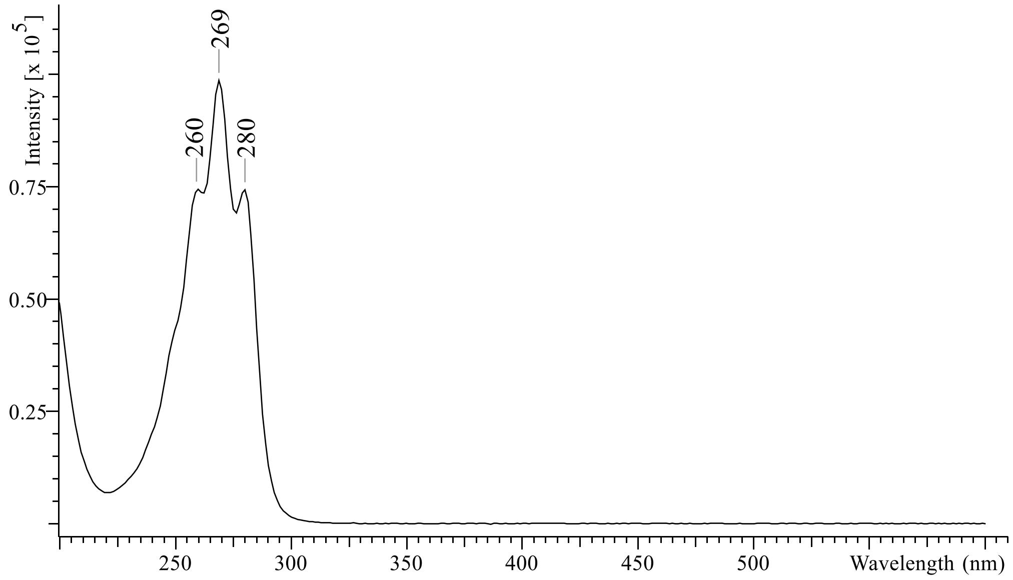

Figure S2 UV-Vis profile of *E. gracilis* metabolites showing absorbance maxima at 260, 269 and 280 nm.

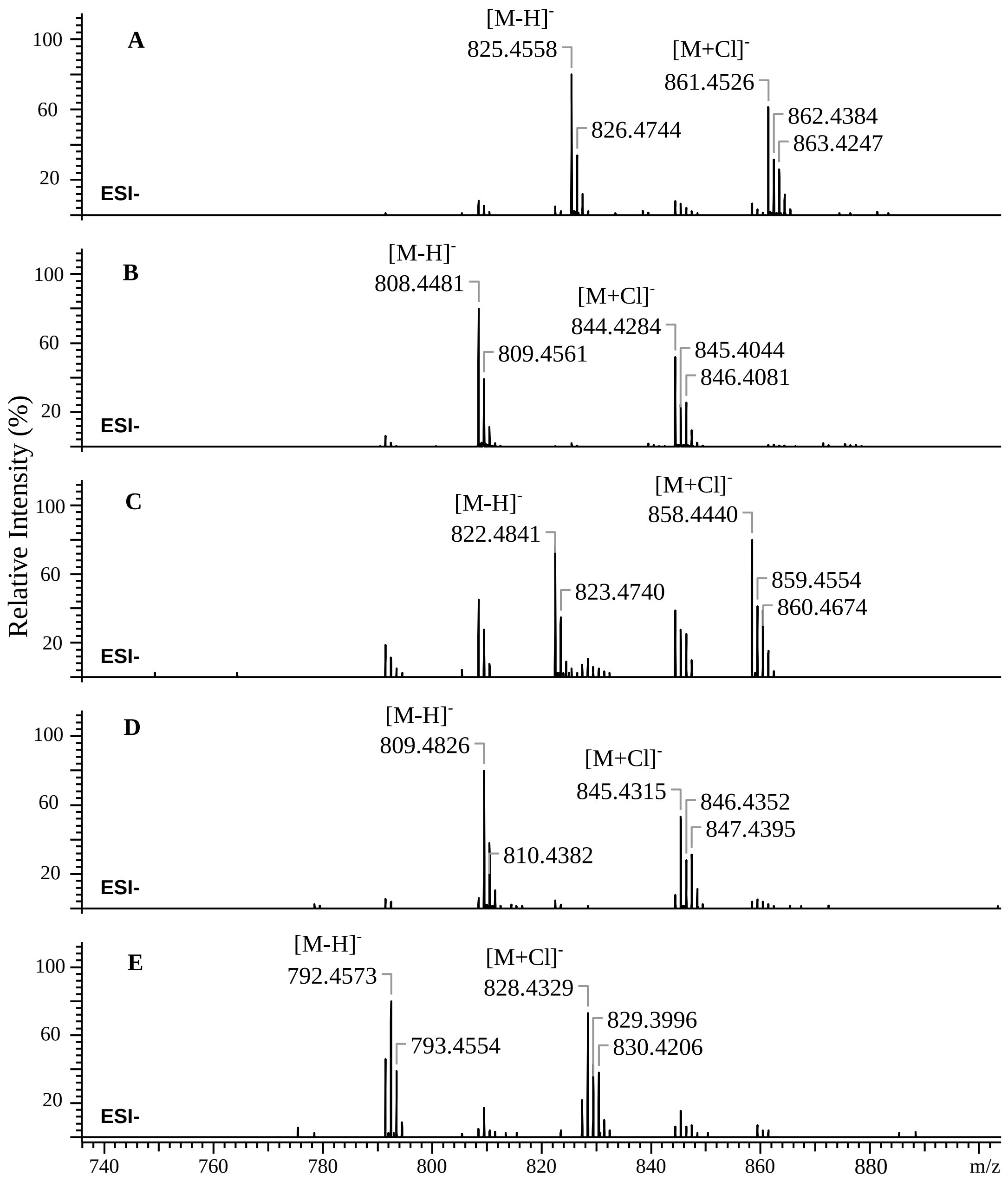

Figure S3 Negative ion mode ESI-MS spectra of euglenatides A, B, C, D and E showing $[M-H]^-$ and $[M+Cl]^-$.

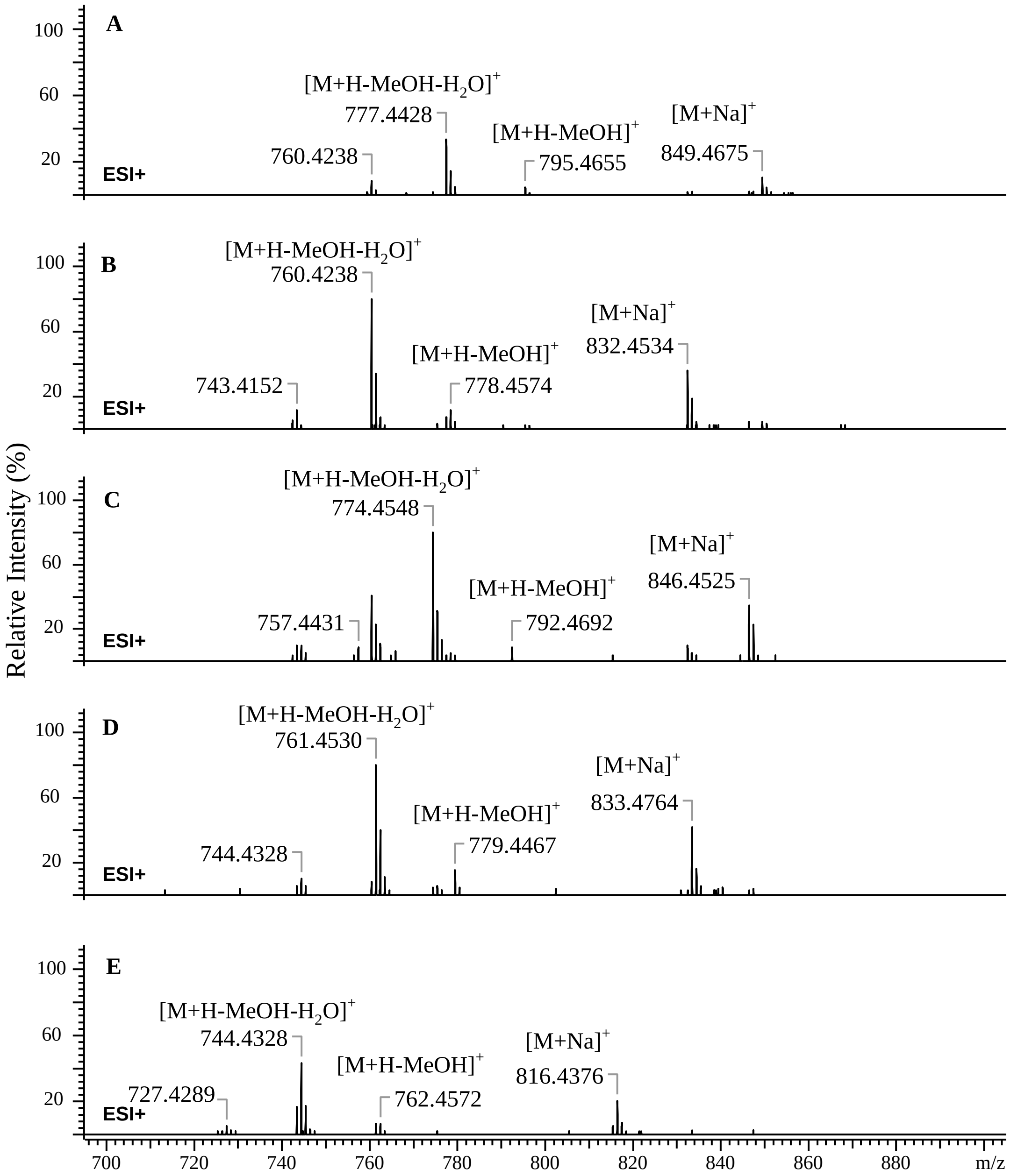

Figure S4 Positive ion mode MS spectra of euglenatides A, B, C, D and E showing $[M+Na]^+$ and two characteristic protonated product ions corresponding to a neutral loss of 50 Da (loss of H_2O and MeOH) and 32 Da (loss of MeOH).

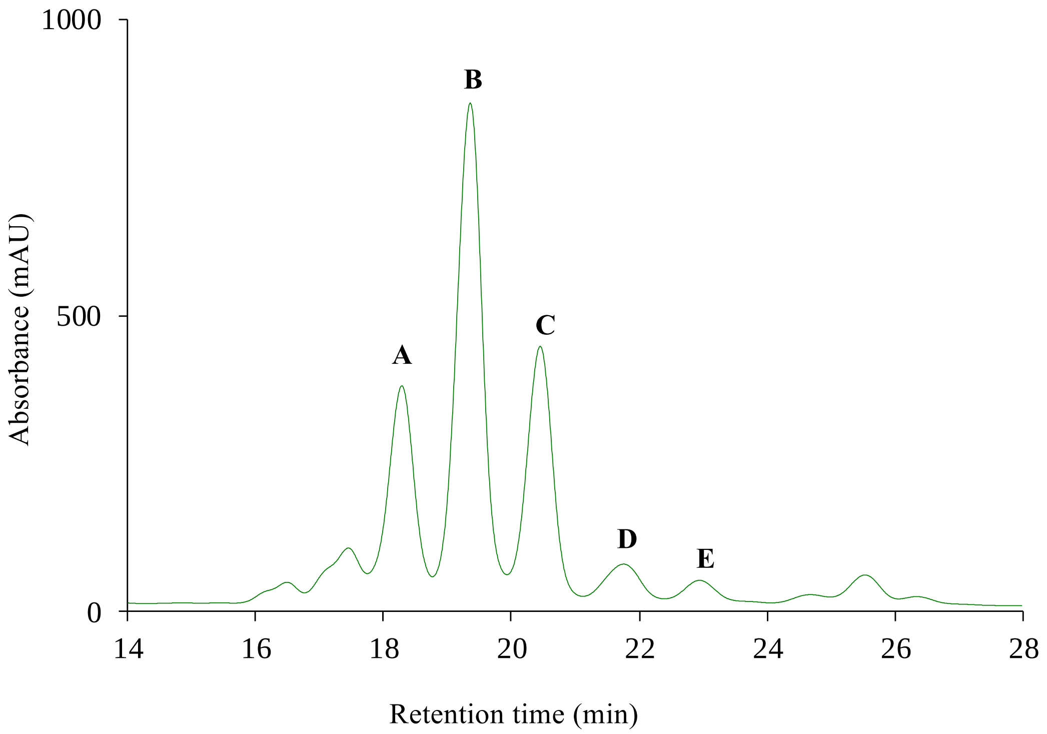

Figure S5 Semi-preparative HPLC trace (at 270 nm) of *E. gracilis* metabolites with euglenatides A-E indicated.

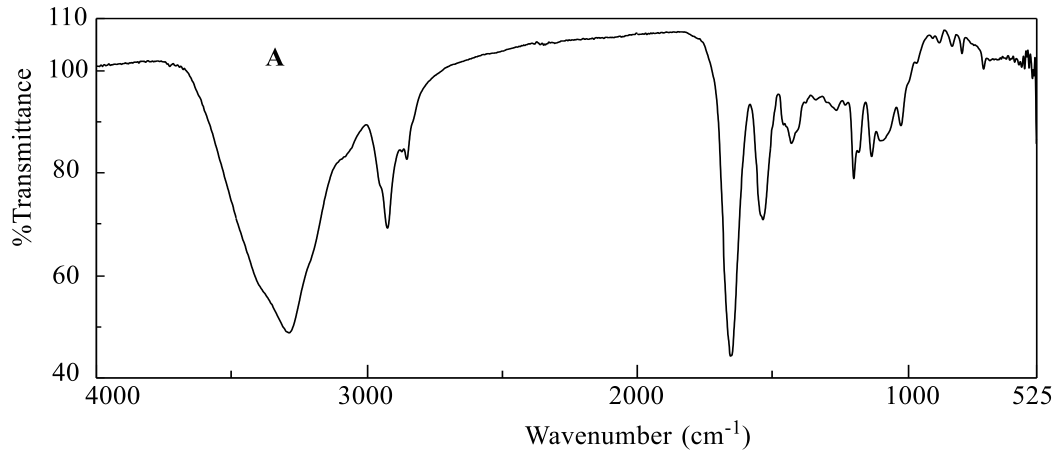

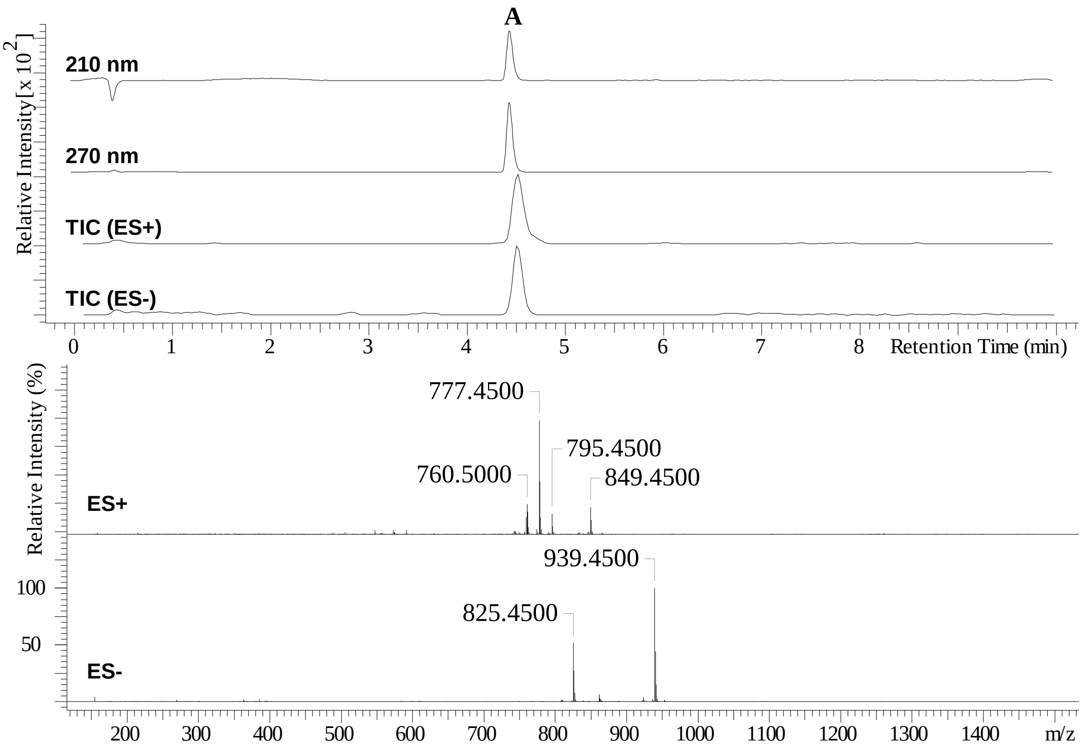

Figure S6 LC-UV-MS chromatograms, IR and MS spectra of euglenatide A.

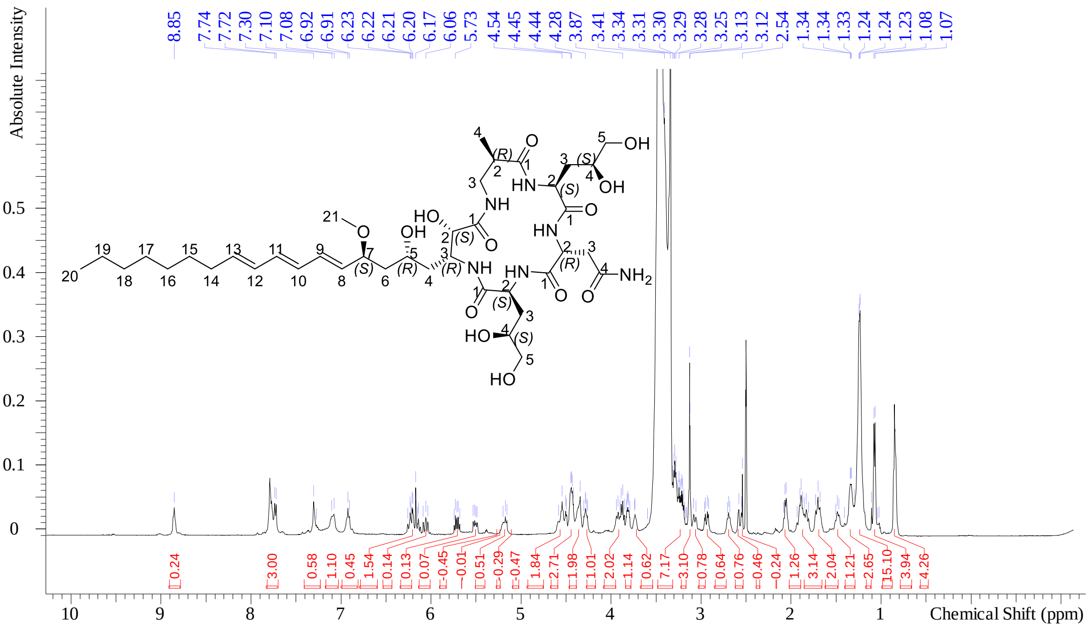

Figure S7 ^{1}H NMR spectrum of euglenatide A in DMSO-d_6.

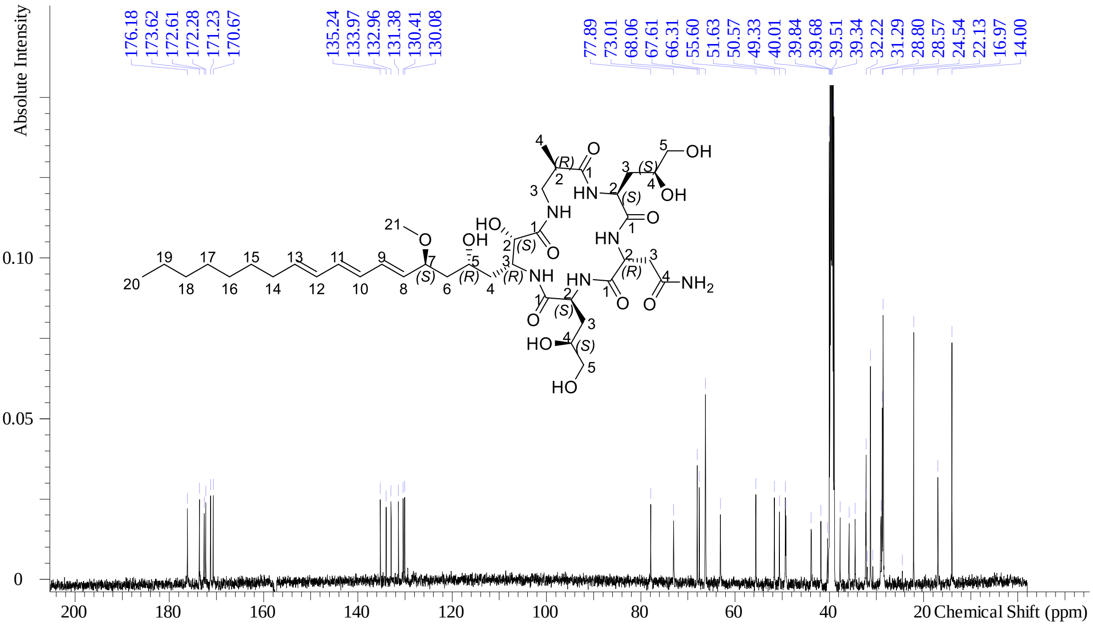

Figure S8 ^{13}C NMR spectrum of euglenatide A in DMSO-d_6.

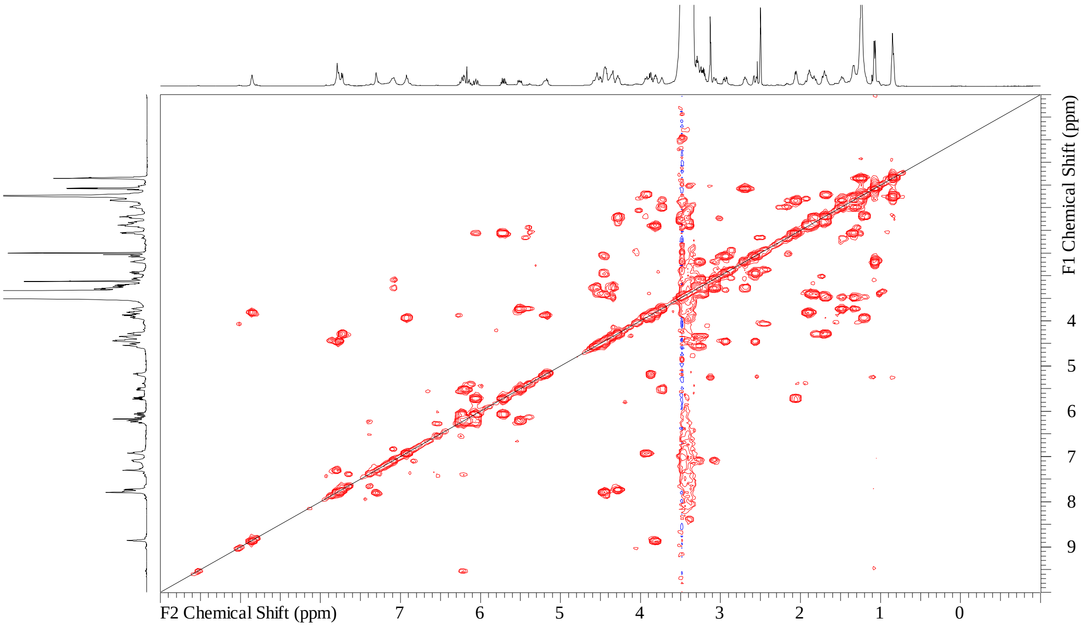

Figure S9 COSY NMR spectrum of euglenatide A in DMSO-d_6.

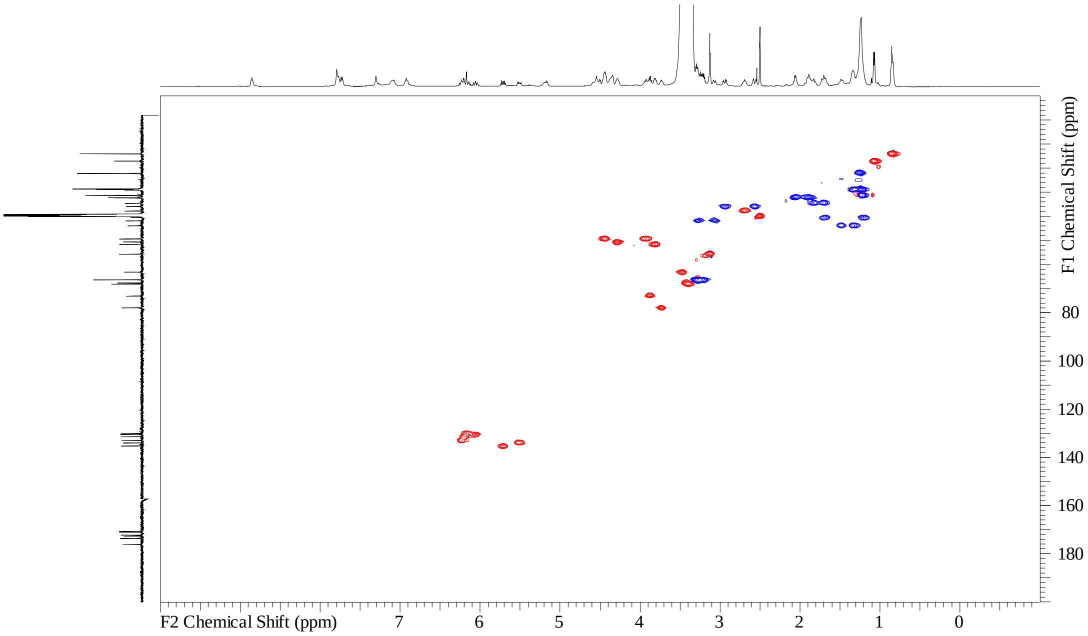

Figure S10 HSQC NMR spectrum of euglenatide A in DMSO-d_6.

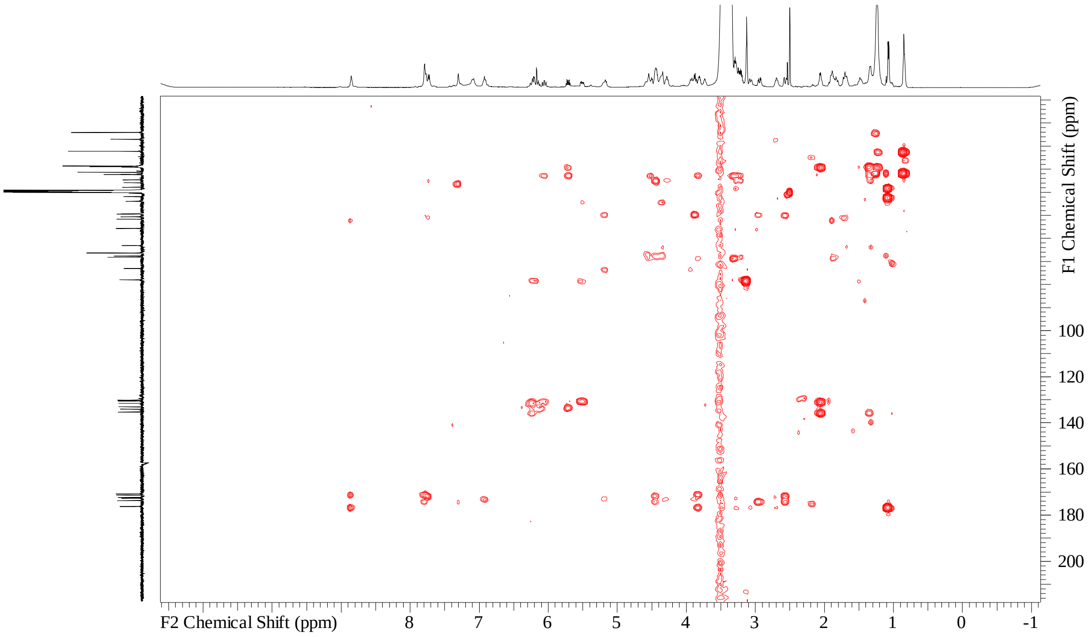

Figure S11 HMBC NMR spectrum of euglenatide A in DMSO-d_6.

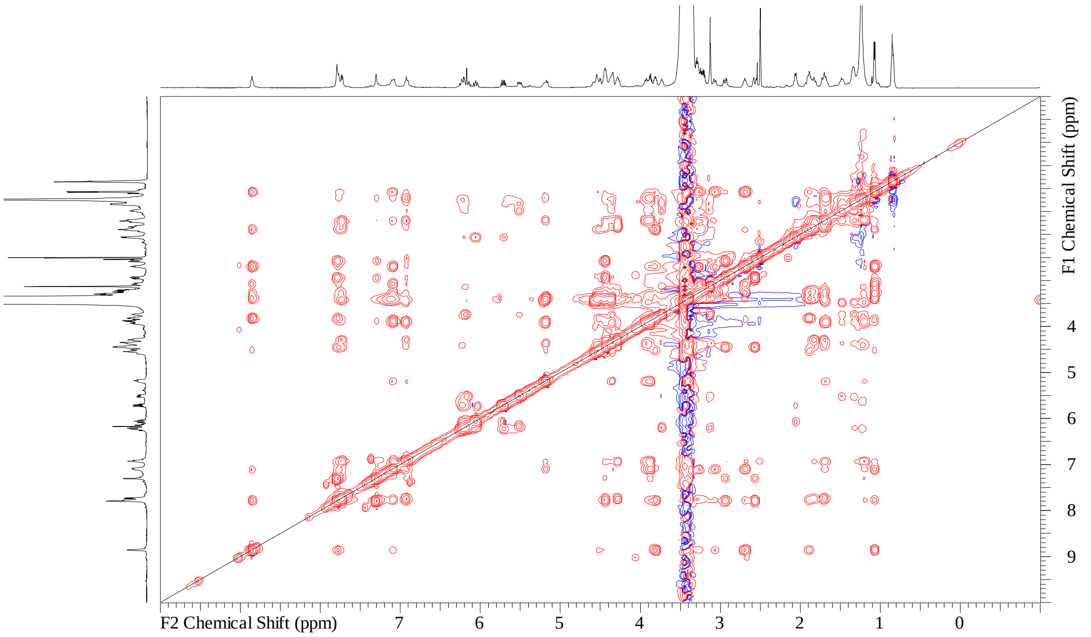

Figure S12 NOESY NMR spectrum of euglenatide A in DMSO-d_6.

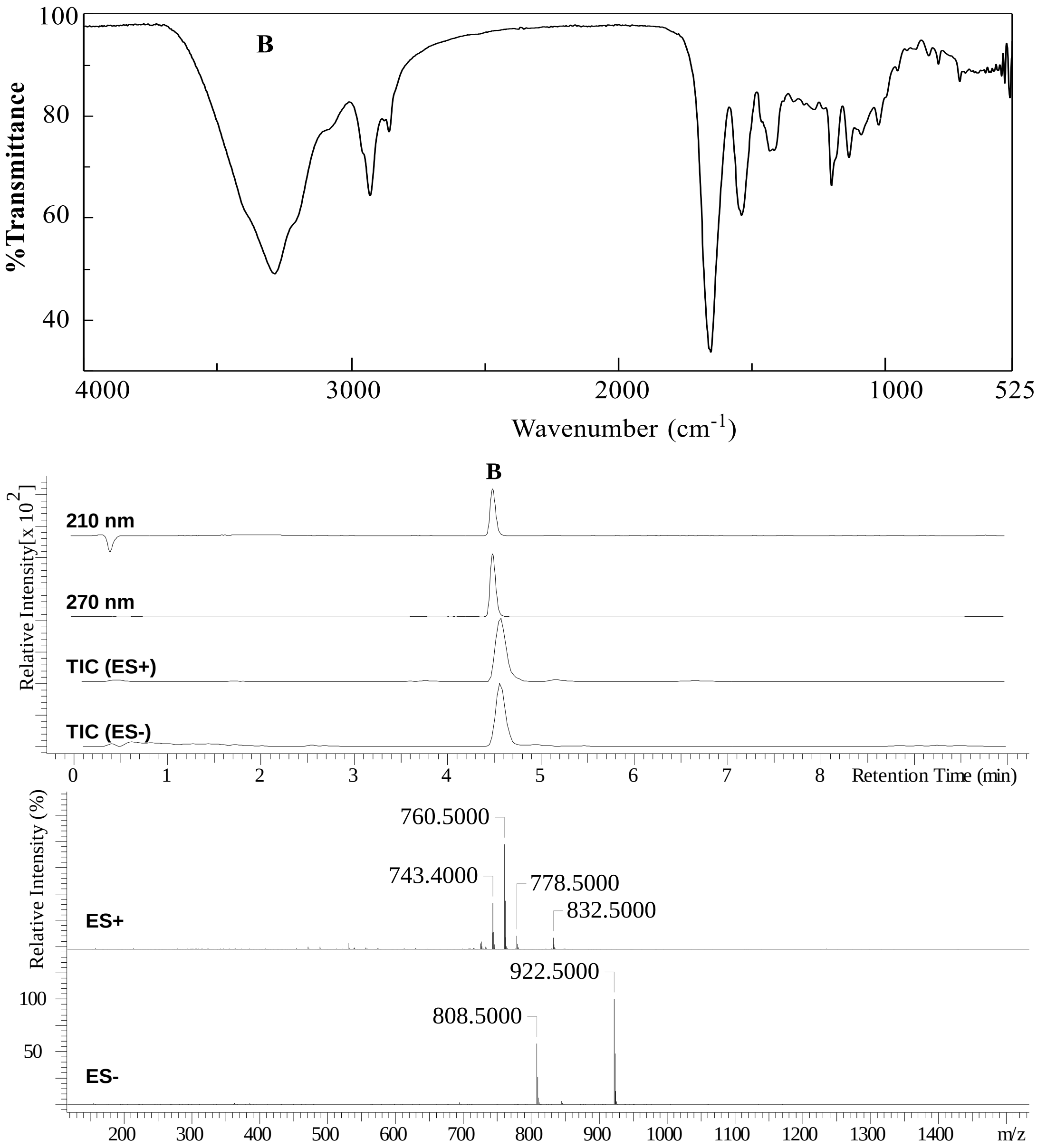

Figure S13 LC-UV-MS chromatograms, IR and MS spectra of euglenatide B.

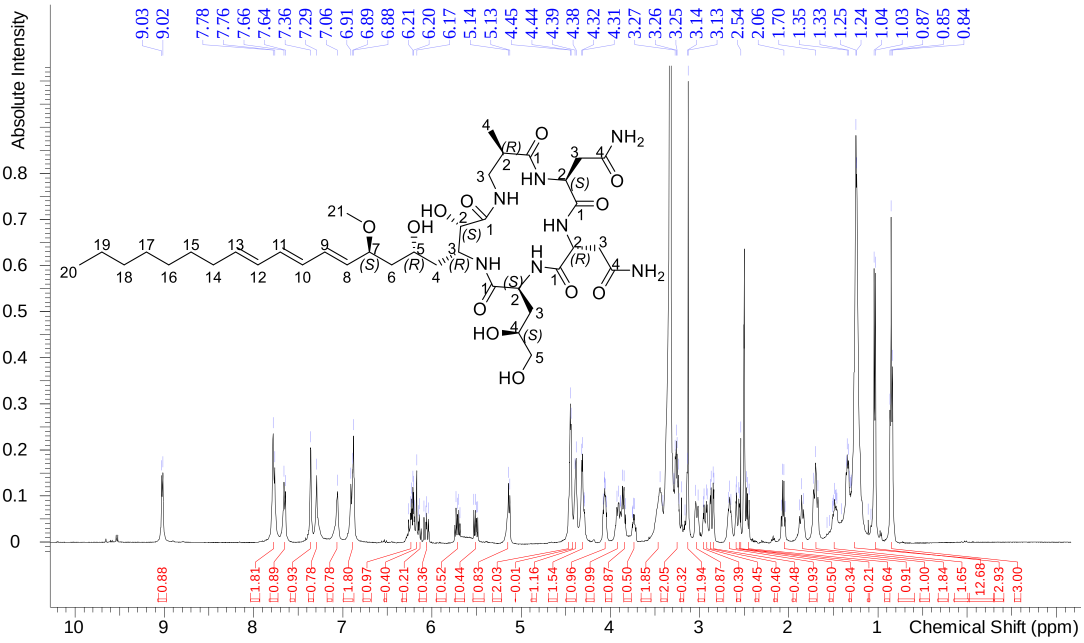

Figure S14 ^{1}H NMR spectrum of euglenatide B in DMSO-d_6.

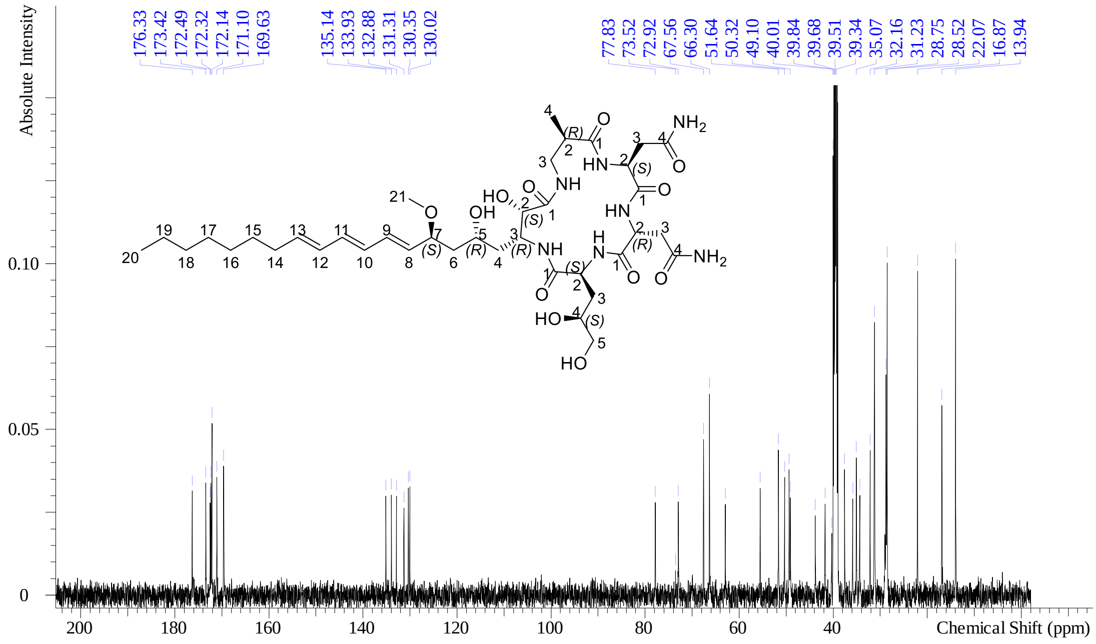

Figure S7 ^{13}C NMR spectrum of euglenatide B in DMSO-d_6.

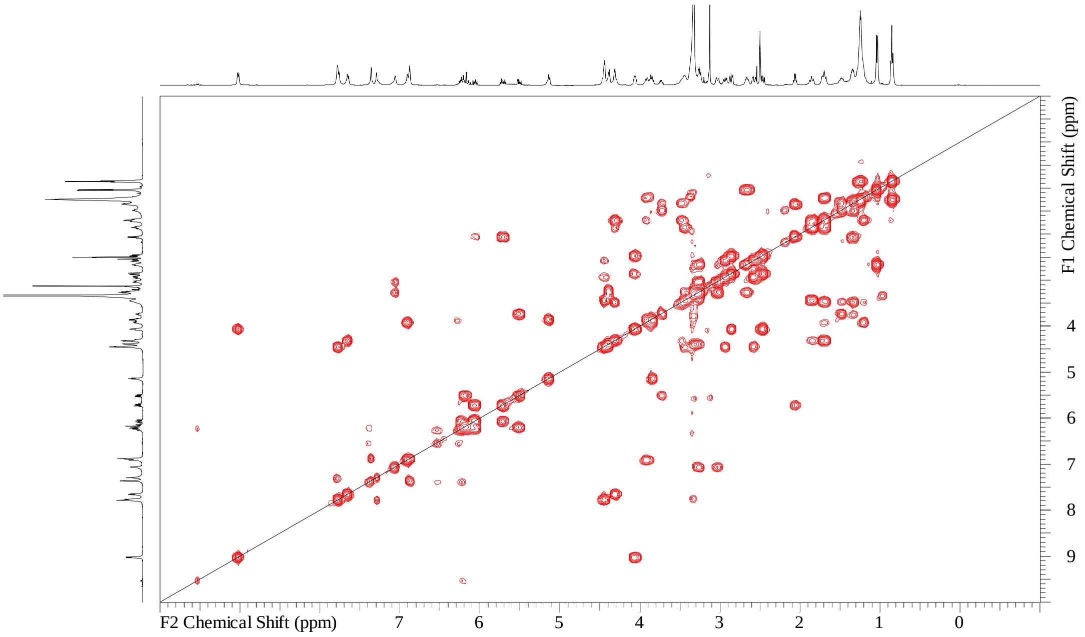

Figure S8 COSY NMR spectrum of euglenatide B in DMSO-d_6.

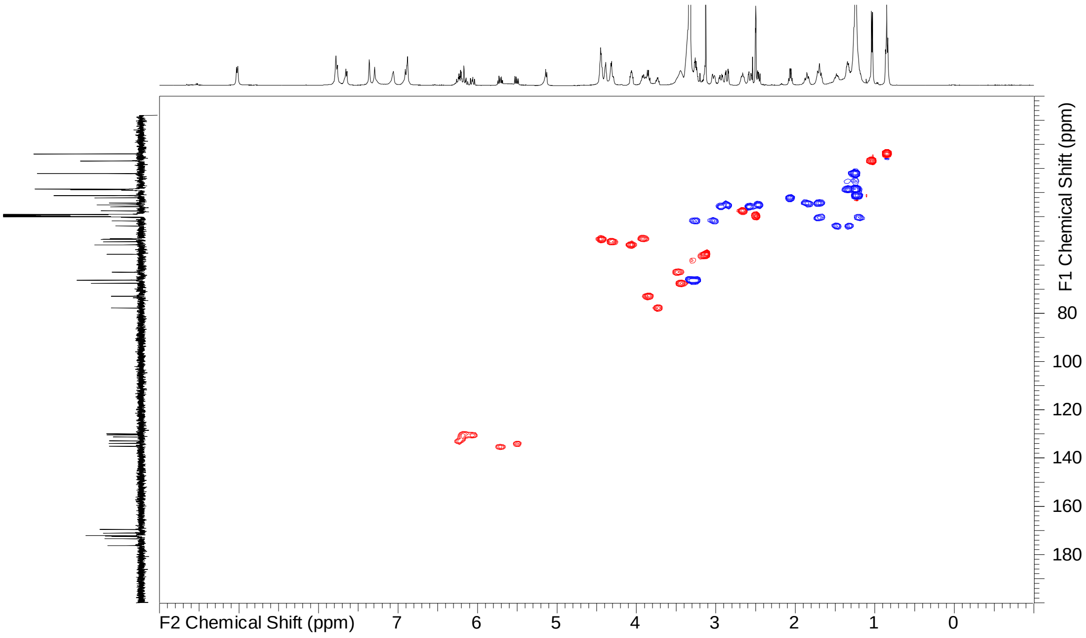

Figure S9 HSQC NMR spectrum of euglenatide B in DMSO-d_6.

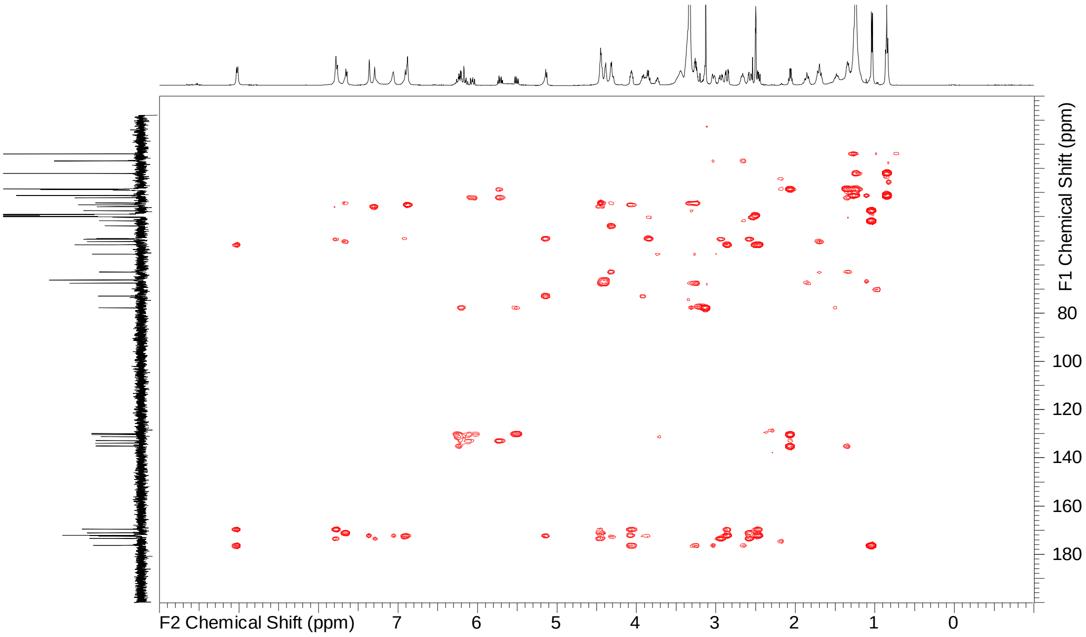

Figure S108 HMBC NMR spectrum of euglenatide B in DMSO-d_6.

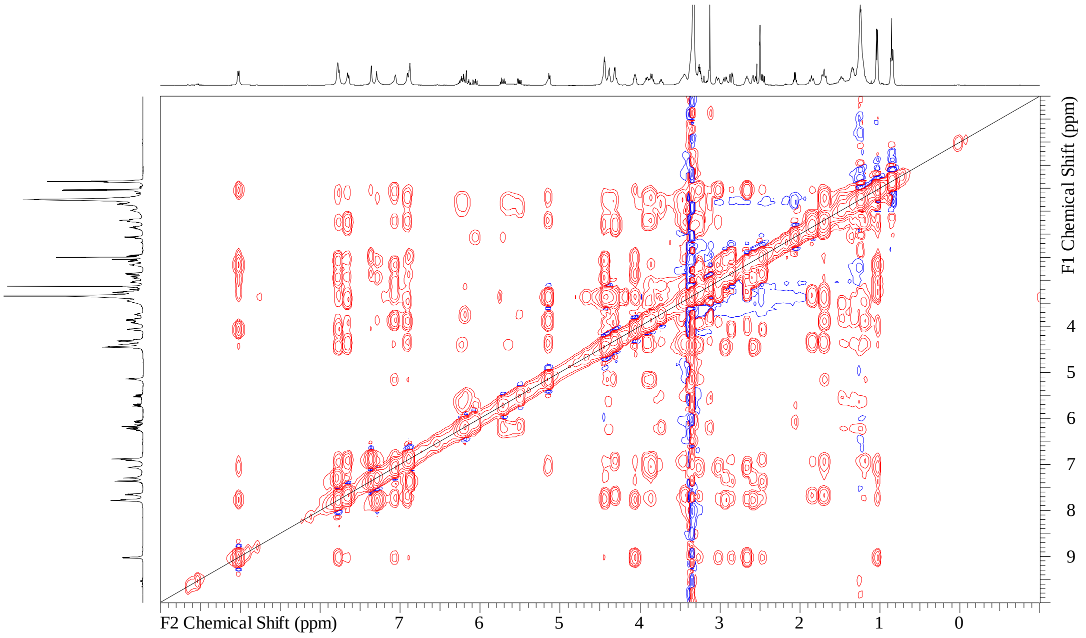

Figure S11 NOESY NMR spectrum of euglenatide B in DMSO-d_6.

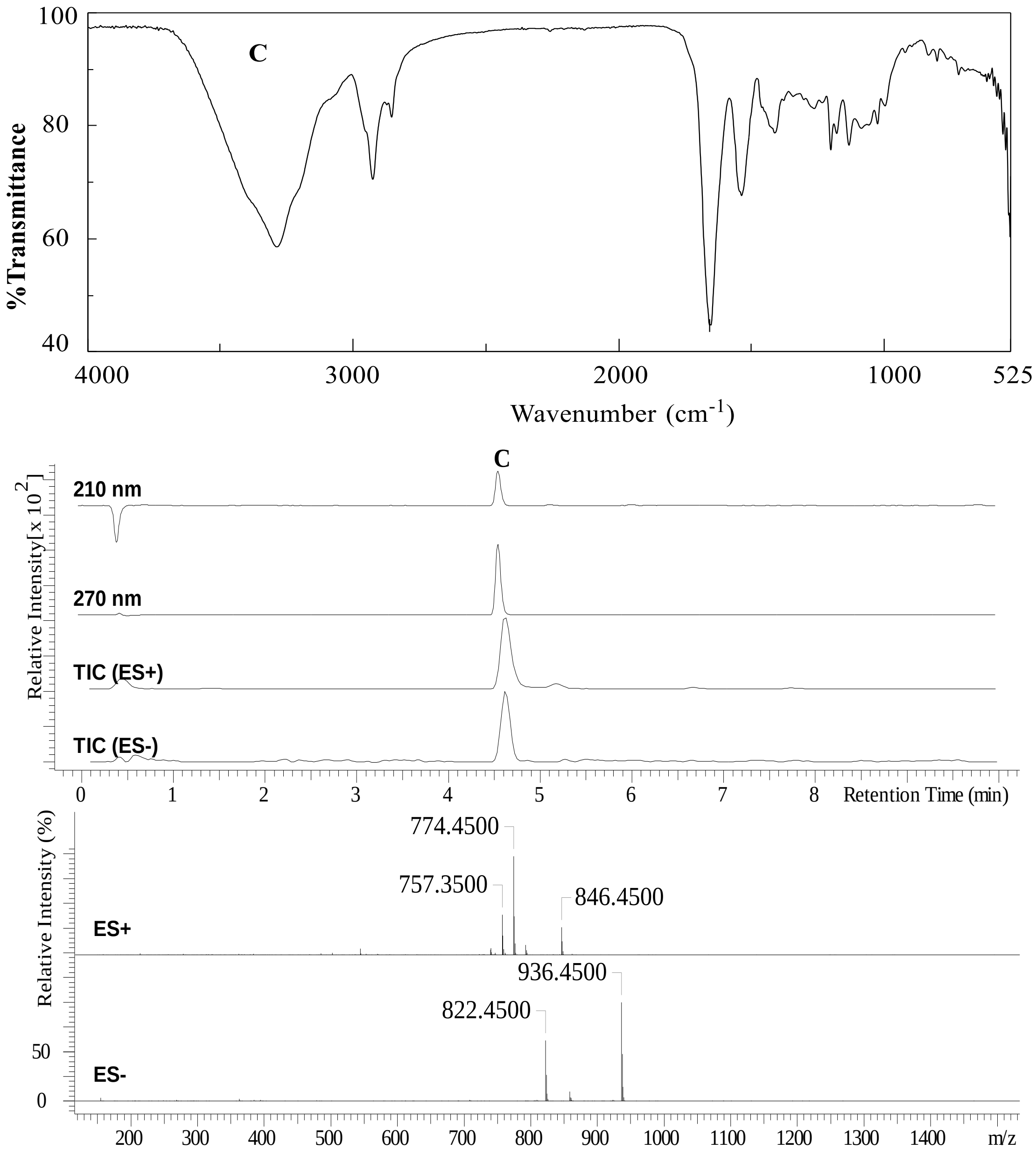

Figure S20 LC-UV-MS chromatograms, IR and MS spectra of euglenatide C.

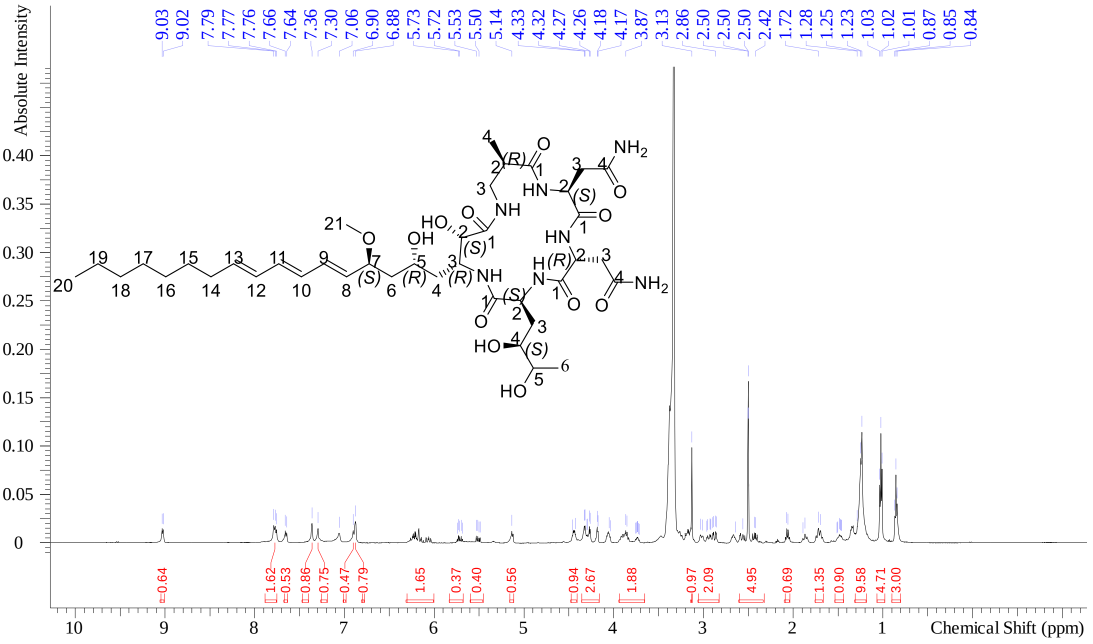

Figure S21 ^{1}H NMR spectrum of euglenatide C in DMSO-d_6.

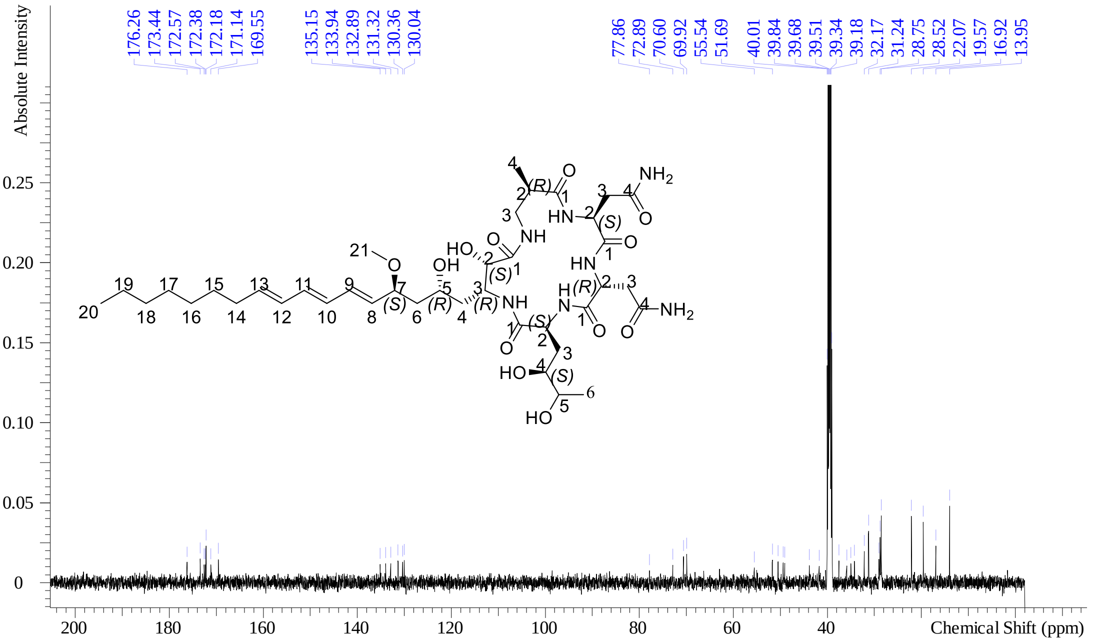

Figure S22 ^{13}C NMR spectrum of euglenatide C in DMSO-d_6.

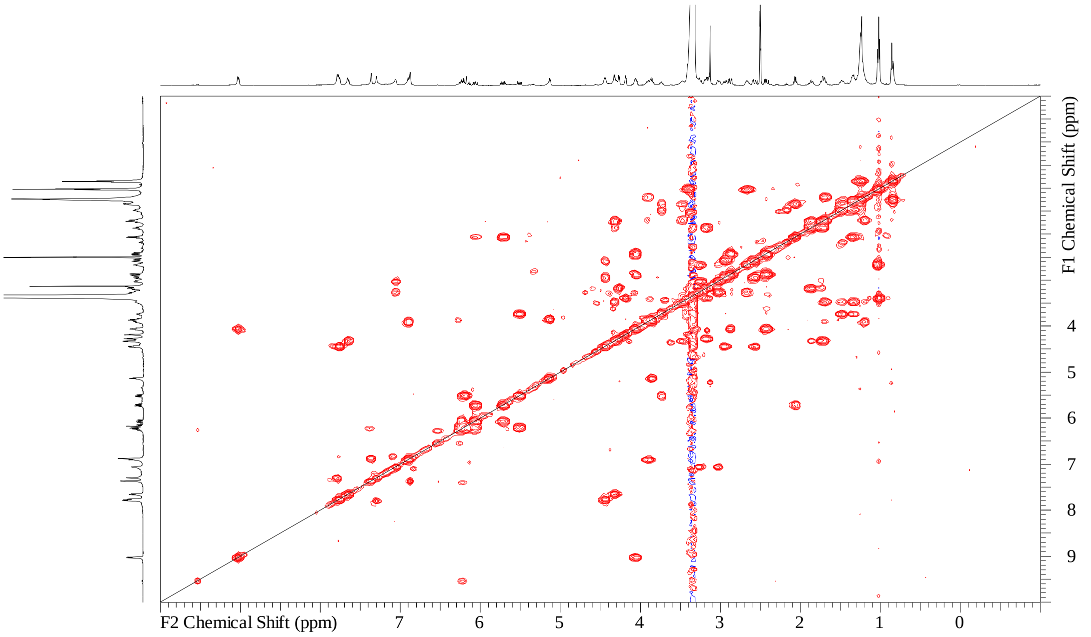

Figure S212 COSY NMR spectrum of euglenatide C in DMSO-d_6.

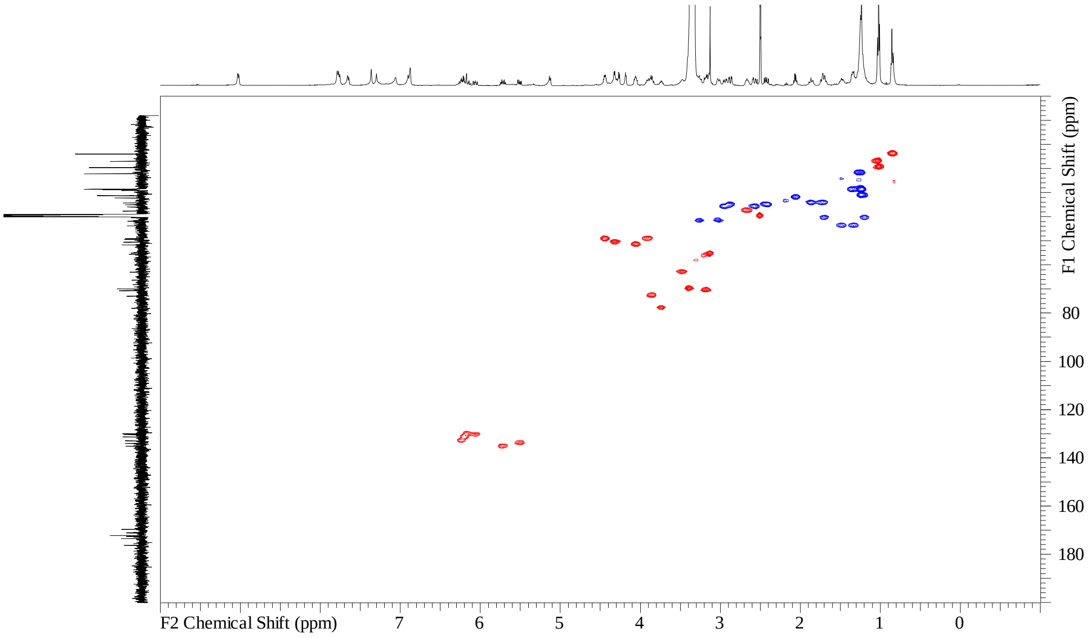

Figure S24 HSQC NMR spectrum of euglenatide C in DMSO-d_6.

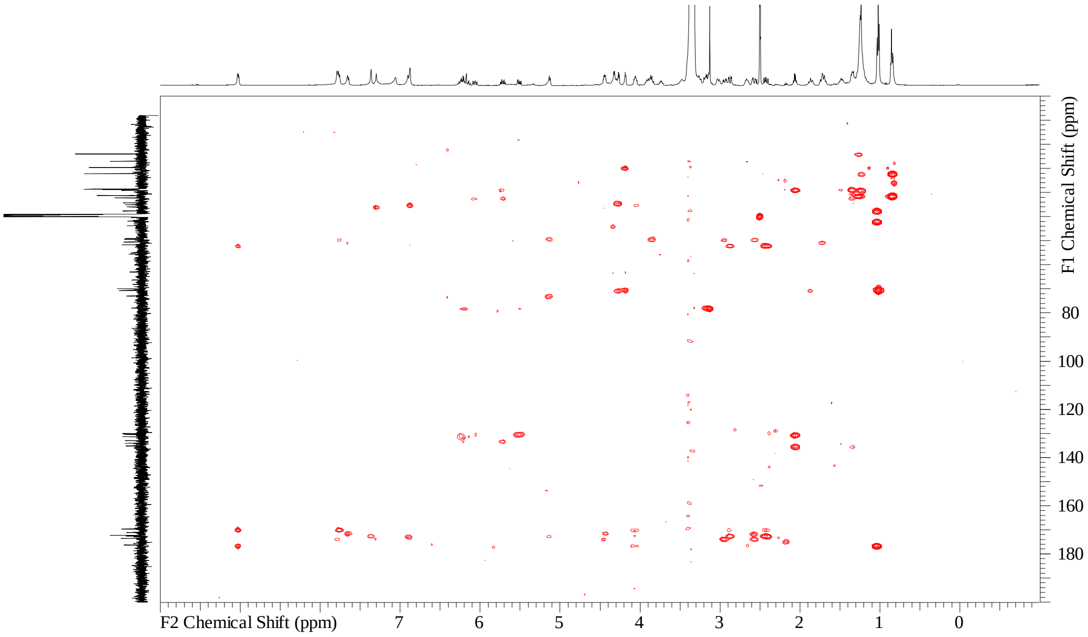

Figure S25 HMBC NMR spectrum of euglenatide C in DMSO-d_6.

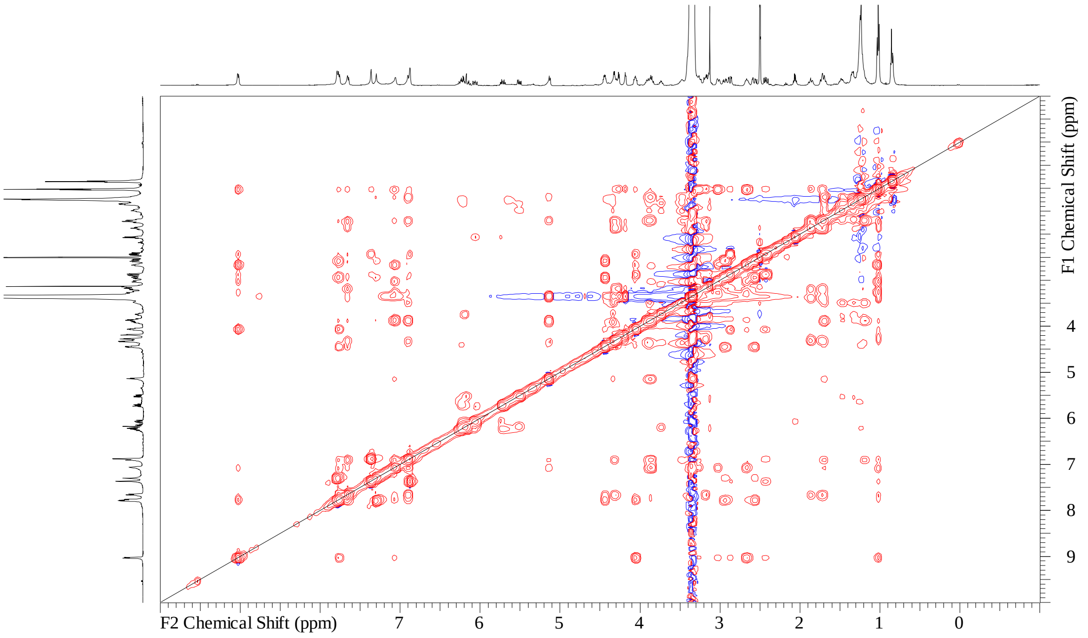

Figure S26 NOESY NMR spectrum of euglenatide C in DMSO-d_6.

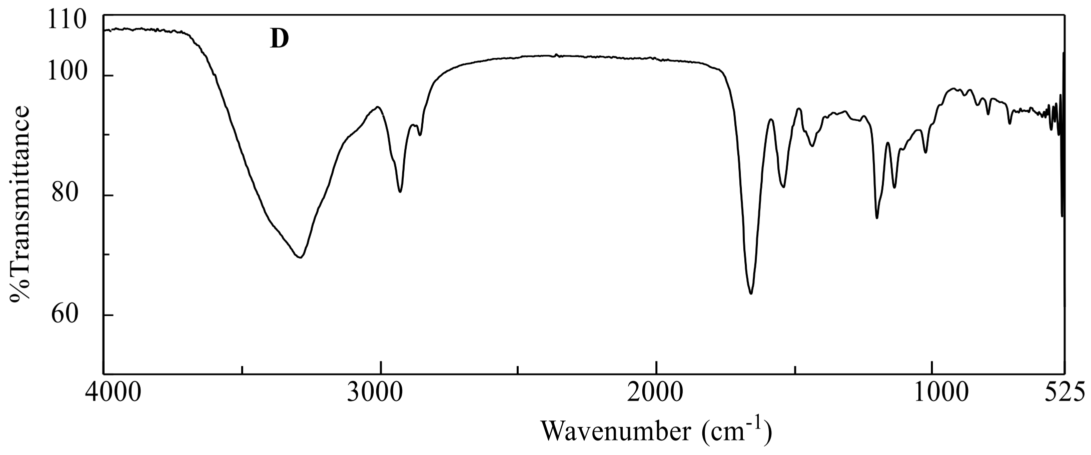

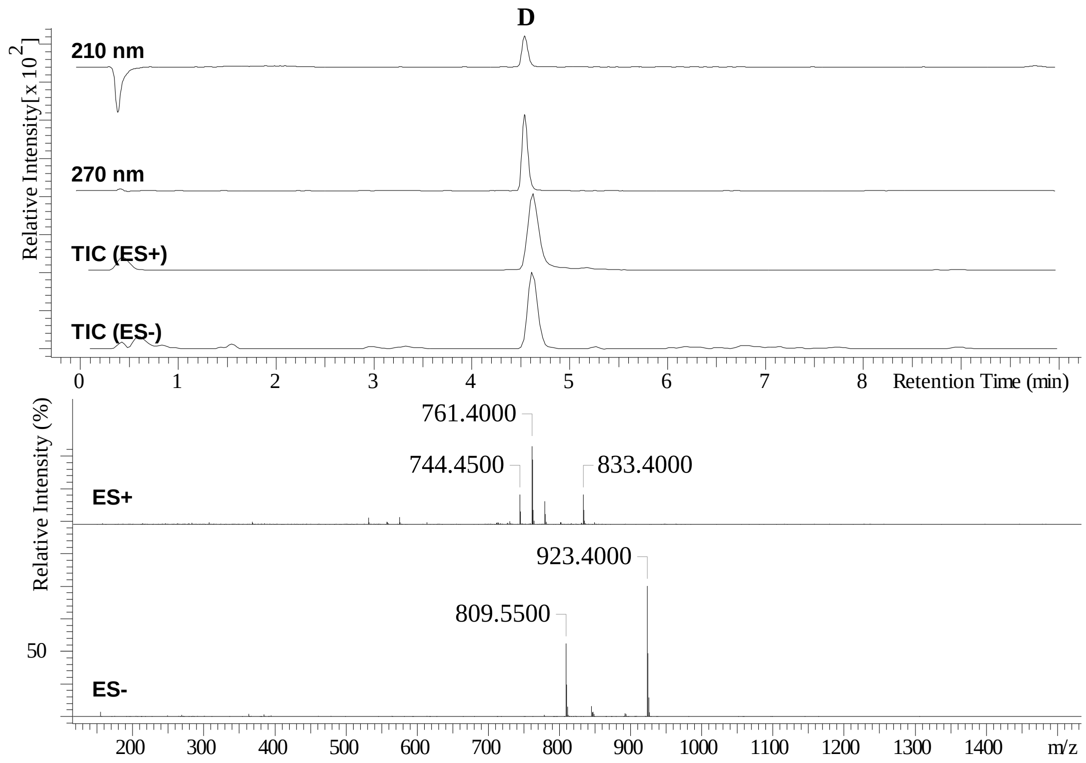

Figure S27 LC-UV-MS chromatograms, IR and MS spectra of euglenatide D.

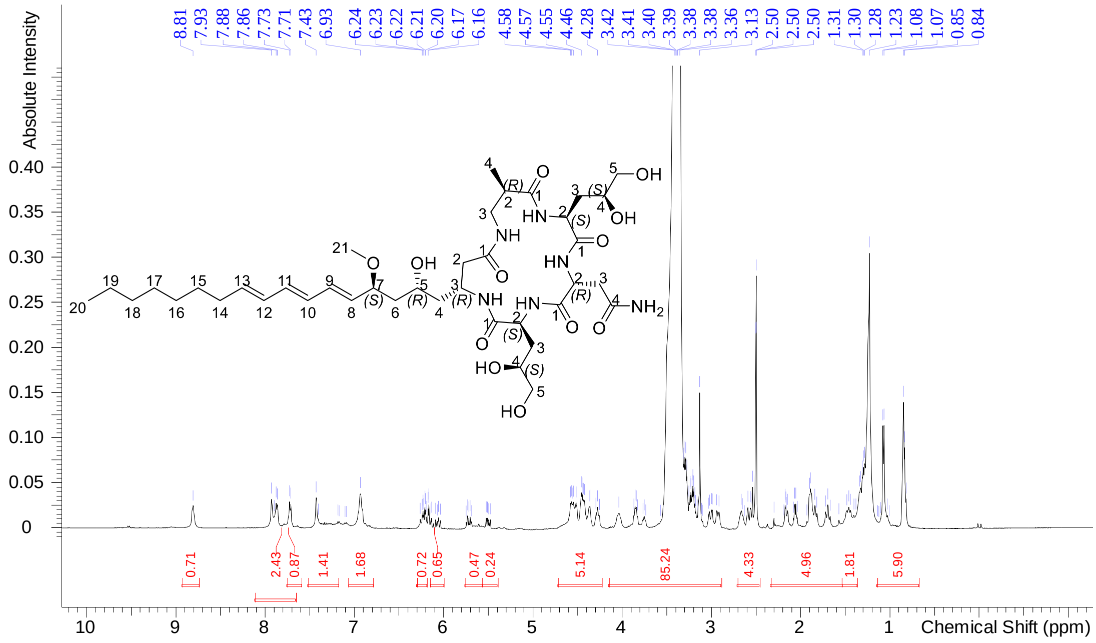

Figure S28 ^{1}H NMR spectrum of euglenatide D in DMSO-d_6.

^{13}C NMR spectrum of euglenatide D in DMSO-d_6

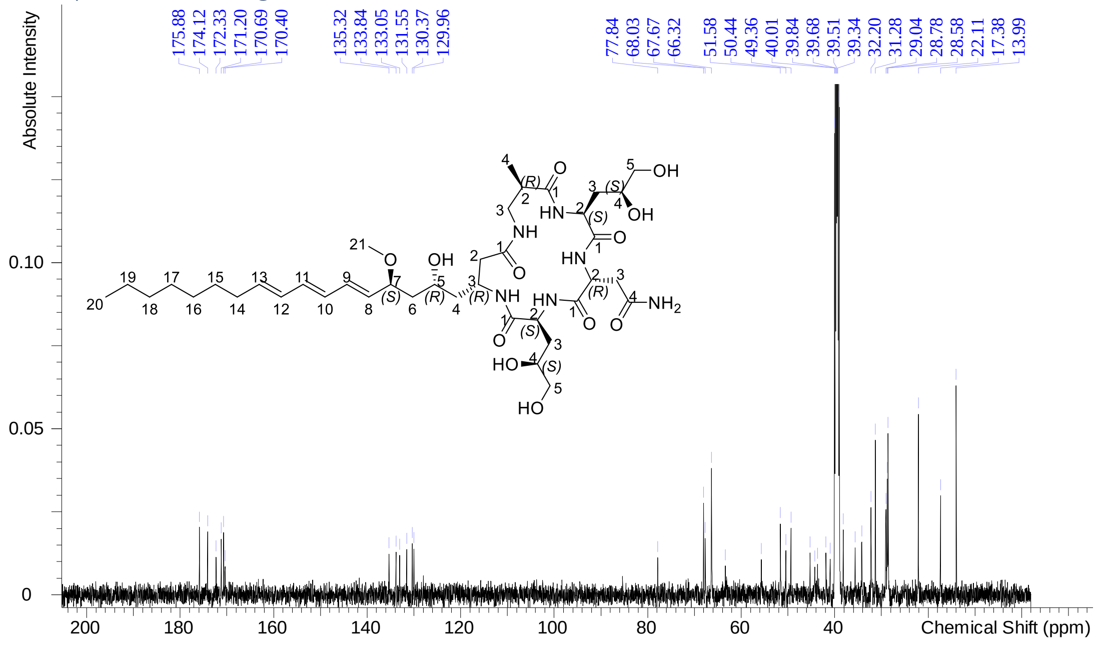

Figure S29 ^{13}C NMR spectrum of euglenatide D in DMSO-d_6.

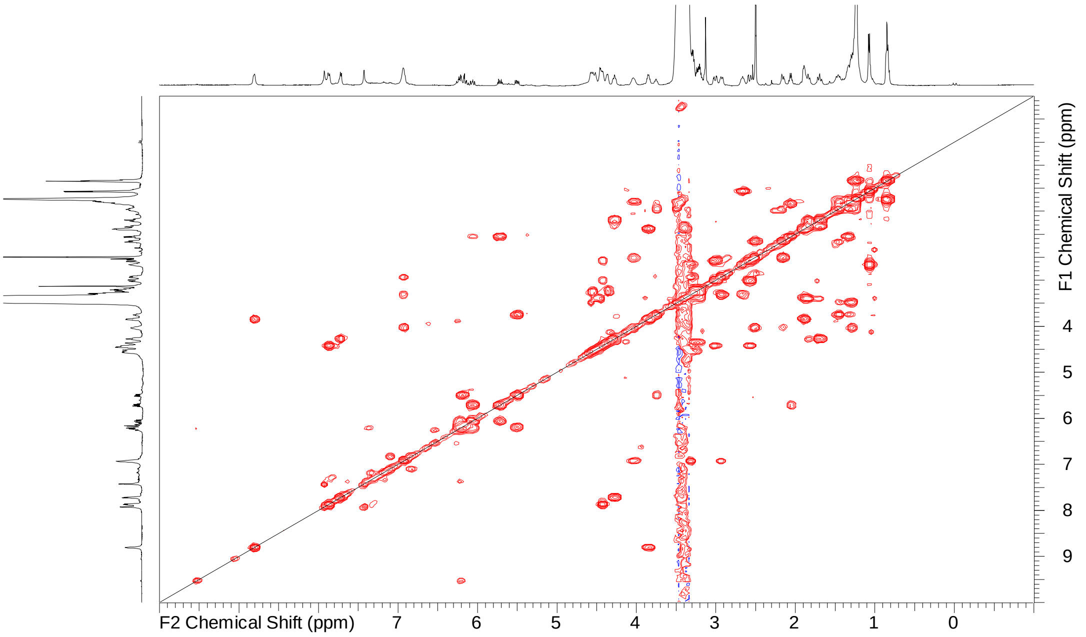

Figure S30 COSY NMR spectrum of euglenatide D in DMSO-d_6.

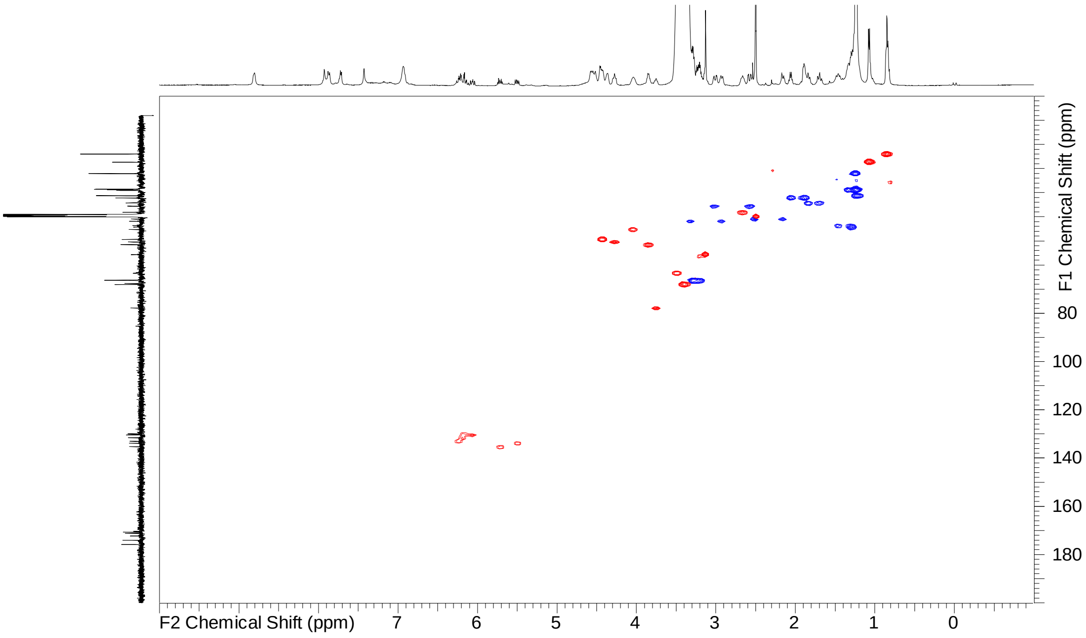

Figure S31 HSQC NMR spectrum of euglenatide D in DMSO-d_6.

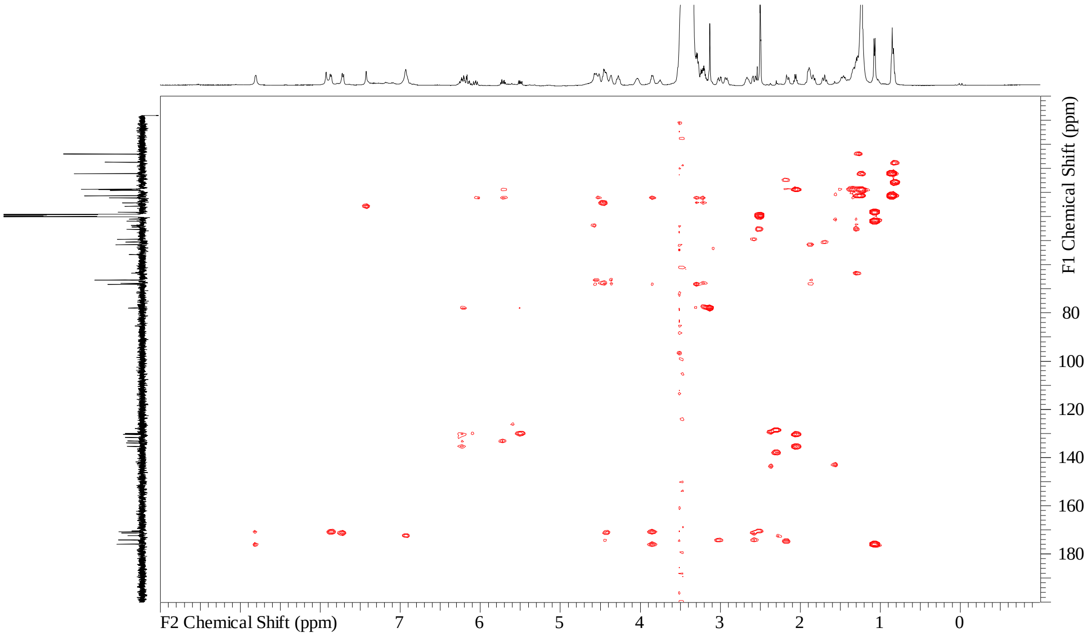

Figure S32 HMBC NMR spectrum of euglenatide D in DMSO-d_6.

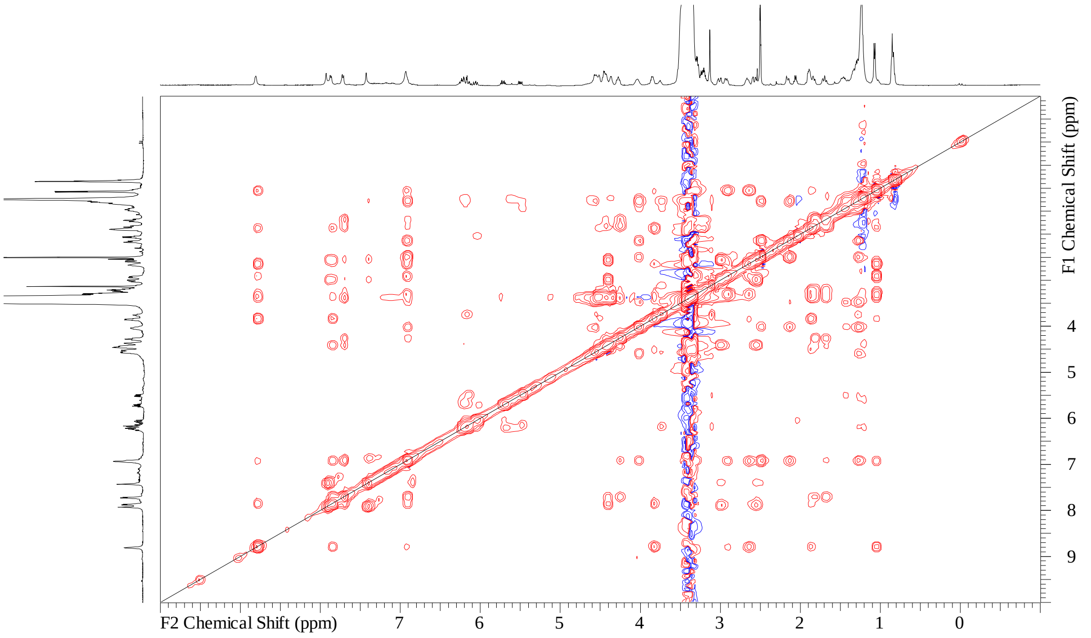

Figure S33 NOESY NMR spectrum of euglenatide D in DMSO-d_6.

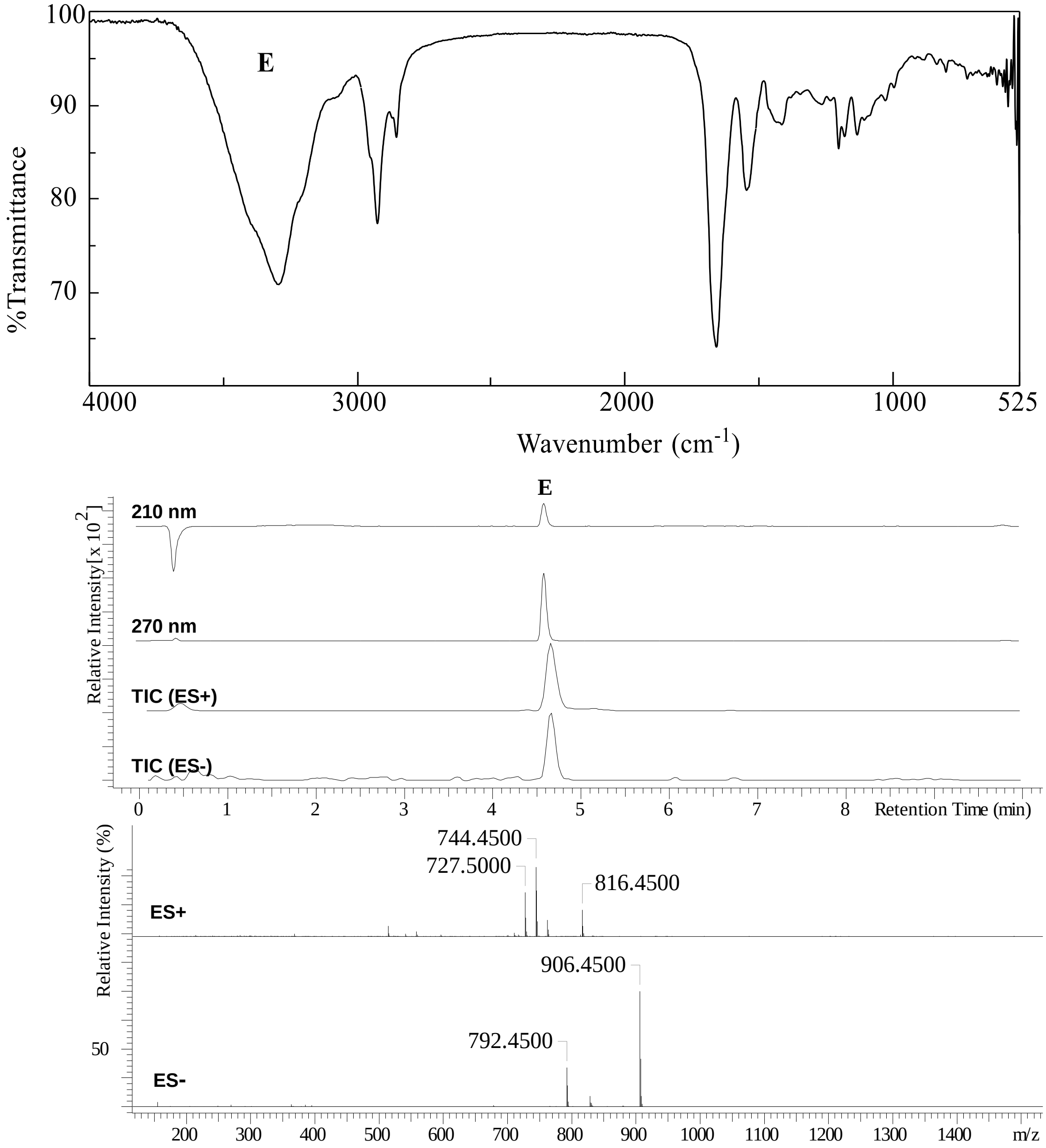

Figure S34 LC-UV-MS chromatograms, IR and MS spectra of euglenatide E.

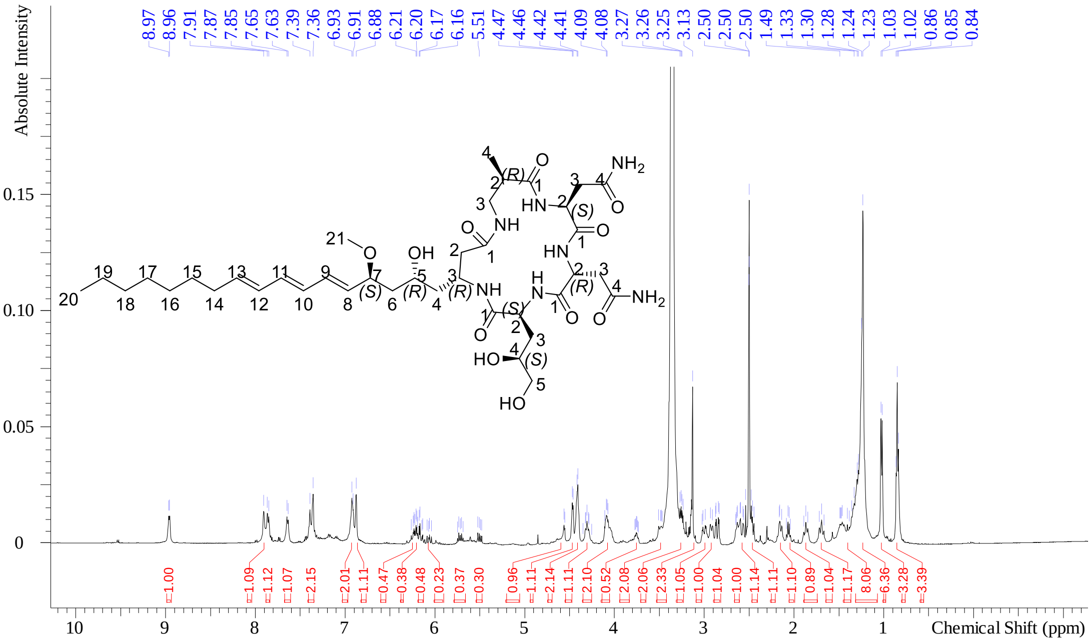

Figure S13 ¹H NMR spectrum of euglenatide E in DMSO-d_6.

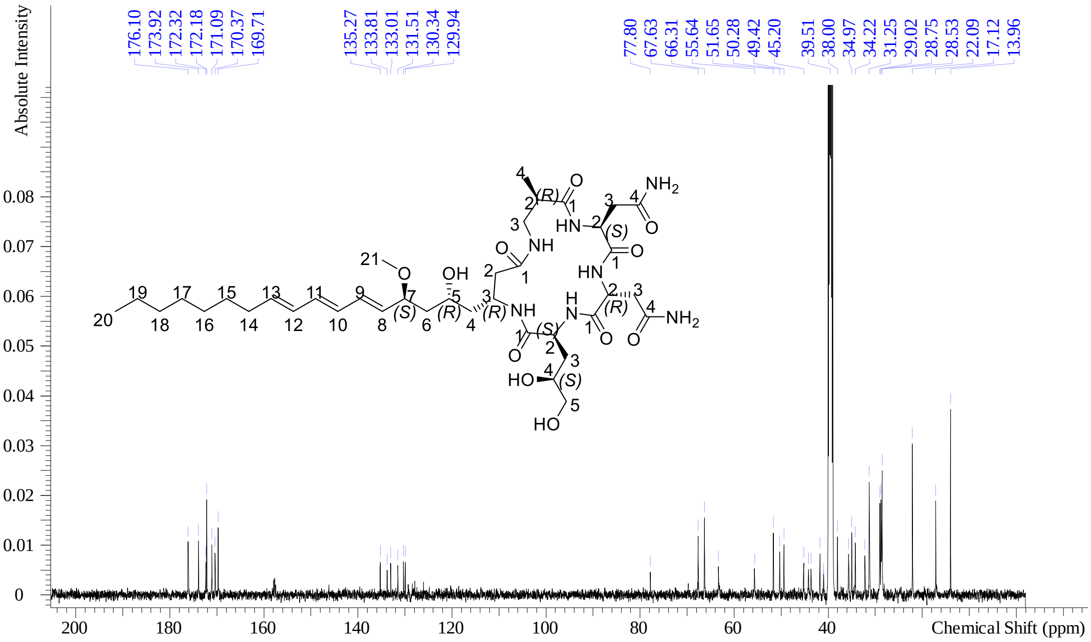

Figure S14 ¹³C NMR spectrum of euglenatide E in DMSO-d_6.

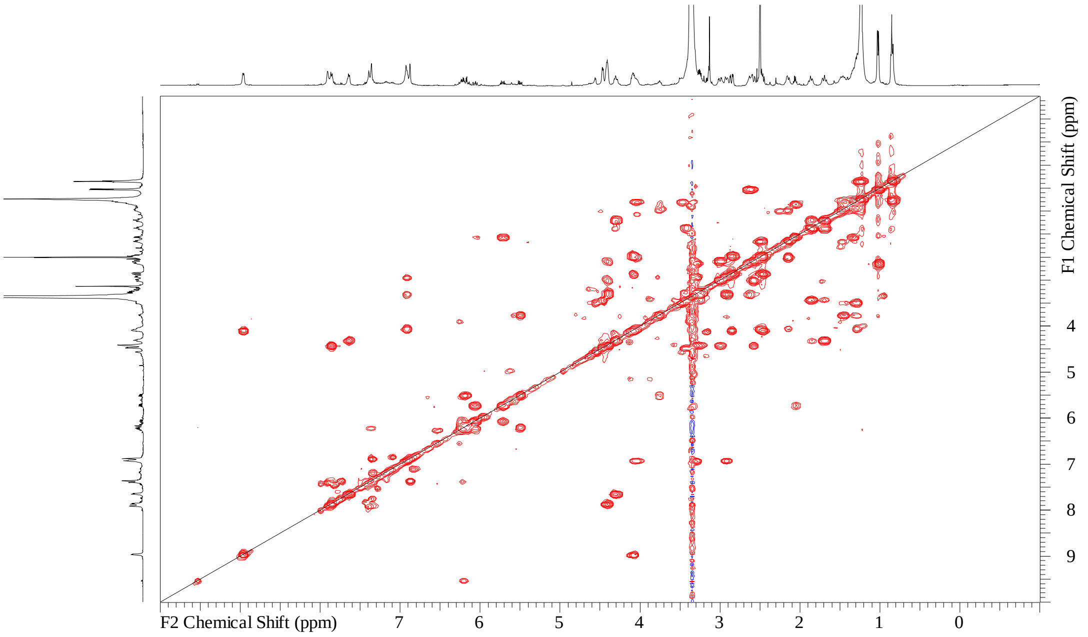

Figure S15 COSY NMR spectrum of euglenatide E in DMSO-d_6.

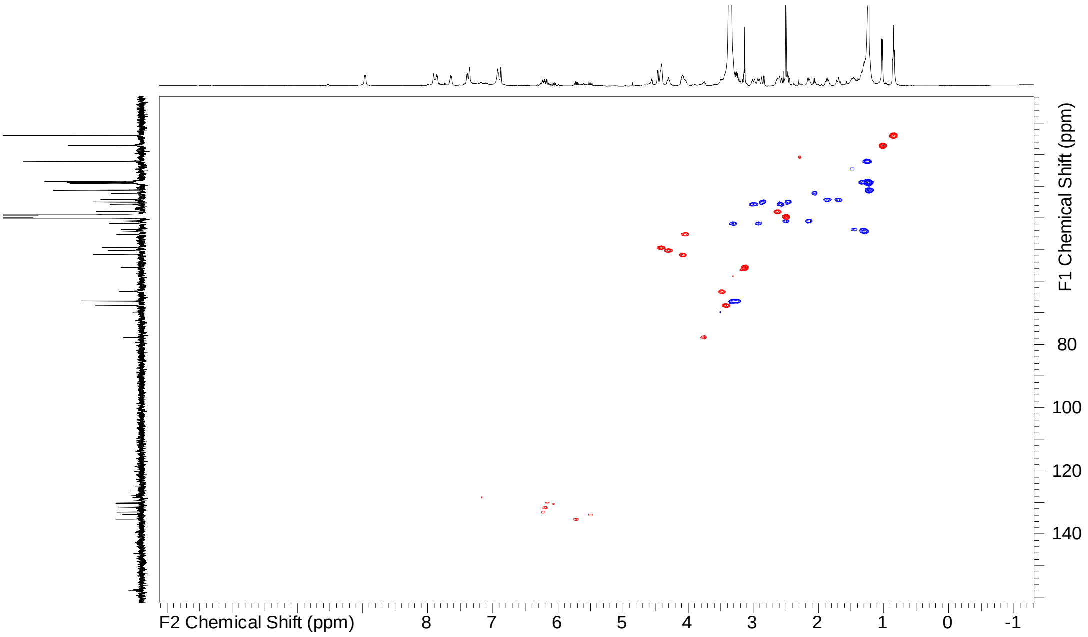

Figure S16 HSQC NMR spectrum of euglenatide E in DMSO-d_6.

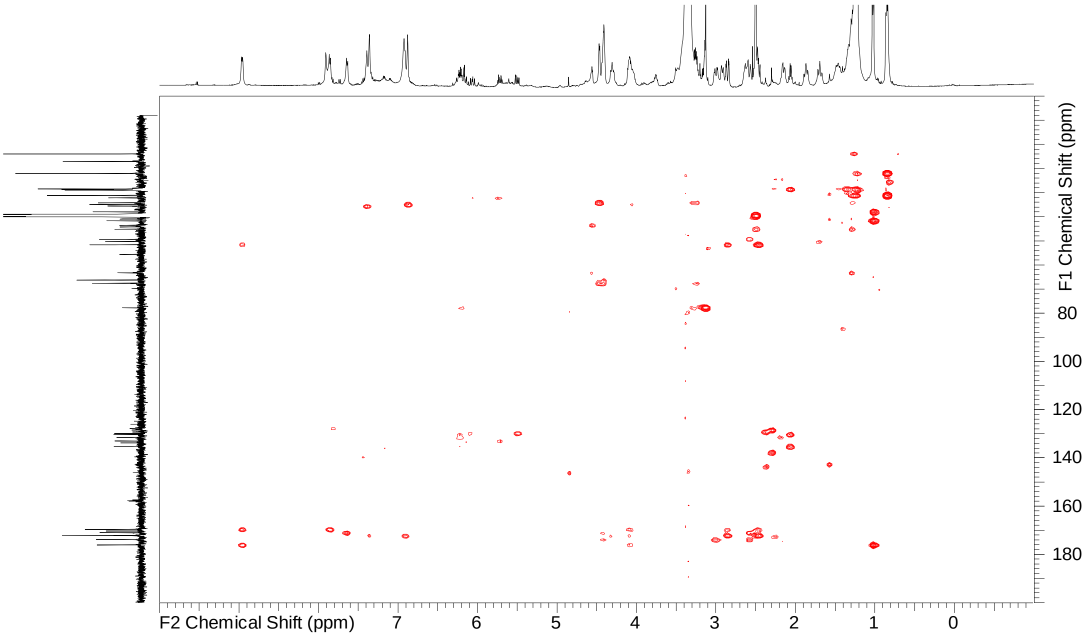

Figure S17 HMBC NMR spectrum of euglenatide E in DMSO-d_6.

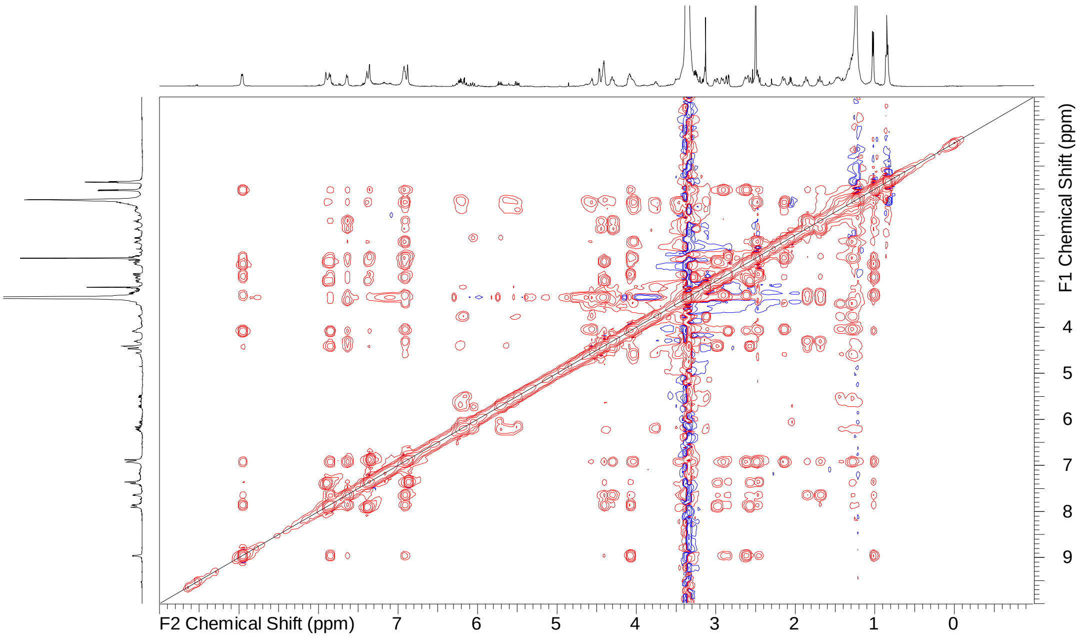

Figure S18 NOESY NMR spectrum of euglenatide E in DMSO-d_6.

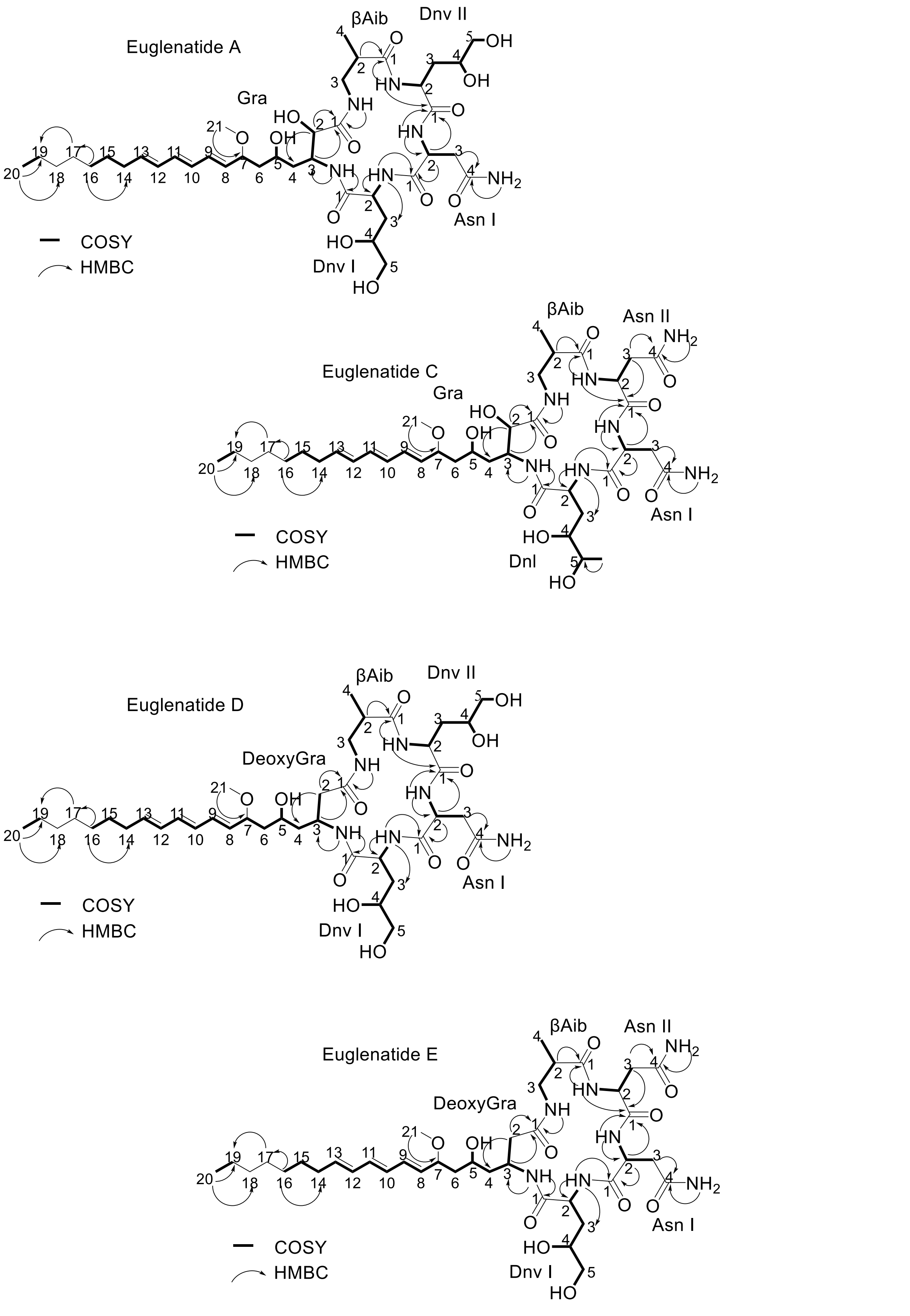

Figure S19 Key COSY and HMBC correlations used to establish the molecular connectivity of euglenatides, A, C, D and E.

Table S1 EG (*E. gracilis*) medium.

**Components**	**Per 1000 mL**
Sodium acetate trihydrate	1 g
Tryptone	3 g
Yeast extract	2 g
Calcium chloride	10 mg
Add constituents above to 1 litre of deionized water and autoclave at 15 psi for 15 minutes.	

Table S2 JM (Jaworski's medium).

**Stock solutions**	**Per 200 mL**
(1) $Ca(NO_3)_2.4H_2O$	4.0 g
(2) KH_2PO_4	2.48 g
(3) $MgSO_4.7H_2O$	10.0 g
(4) $NaHCO_3$	3.18 g
(5) EDTAFeNa	0.45 g
$EDTANa_2.2H_2O$	0.495 g
(6) H_3BO_3	0.496 g
$MnCl_2.4H_2O$	0.278 g
$(NH_4)_6Mo_7O_{24}.4H_2O$	0.20 g
(7) Cyanocobalamin	0.008 g
Thiamine HCl	0.008 g
Biotin	0.008 g
(8) $NaNO_3$	16.0 g
(9) Na_2HPO_4	7.2 g
Take 1 mL of each stock solution, make up to 1 litre with deionized water and autoclave at 15 psi for 15 minutes.	

Table S3 ^{1}H and ^{13}C NMR data (500 and 125 MHz, DMSO-d_6) of euglenatide B.

Position (No.)	^{13}C ppm	^{1}H ppm mult. (*J* in Hz)	Position (No.)	^{13}C ppm	^{1}H ppm mult. (*J* in Hz)
Dnv			βAib		
C (1)	172.4		C (1)	176.3	
CH (2)	50.3	4.32, m	CH (2)	37.5	2.66, m
NH (2)		7.65, d (7.8)	CH2 (3)	41.7	3.03, brd (13.0), 3.27, m
CH2 (3)	34.3	1.86, m, 1.71, m	NH (3)		7.06, s
CH (4)	67.5	3.43, m	CH3 (4)	16.8	1.04, d (7.1)
OH (4)		4.44, brd (4.7)			
CH2 (5)	66.3	3.32, m, 3.26, m			
OH (5)		4.38, m			
Asn I			Gra		
C (1)	171.1		C (1)	172.3	
CH (2)	49.3	4.45, m	CH (2)	72.9	3.86, dd (9.6, 7.7)
NH (2)		7.77, d (7.9)	OH (2)		5.13, br d (7.7)
CH2 (3)	35.8	2.94, dd (16.8, 5.0) 2.57, dd (16.9, 3.7)	CH (3)	49.1	3.92, m
			NH (3)		6.90 d (9.6)
C (4)	173.4		CH2 (4)	40.3	1.69, m, 1.20, m
NH2 (4)		7.78 s, 7.29 s	CH (5)	63.0	3.47, m
			OH (5)		4.31, m
			CH2 (6)	43.8	1.30, br dd (14.5, 5.1), 1.49, m
Asn II					
C (1)	169.6		CH (7)	77.8	3.73, m
CH (2)	51.6	4.06. m	CH (8)	133.9	5.51, dd (14.6, 7.6)
NH (2)		9.02, d (6.5)	CH (9)	131.3	6.20, dd (14.6, 10.3)
CH2 (3)	35.0	2.86, dd (15.7, 4.0) 2.47, dd (15.9, 4.7)	CH (10)	130.0	6.15, dd (14.6, 10.3)
			CH (11)	132.8	6.24, dd (14.5, 10.4)
C (4)	172.1		CH (12)	130.3	6.07, dd (15.1, 10.4)
NH2 (4)		7.36, s, 6.88, s	CH (13)	135.1	5.72, dt (15.1, 7.1)
			CH2 (14)	32.1	2.06, q (7.0)
			CH2 (15)	28.7	1.35, m
			CH2 (16)	28.52	1.25, m
			CH2 (17)	28.54	1.25, m
			CH2 (18)	31.2	1.24, m
			CH2 (19)	22.0	1.25, m
			CH3 (20)	13.9	0.85, t (6.8)
			CH3 (21)	55.5	3.13, s

Table S4 ^{1}H and ^{13}C NMR data (500 and 125 MHz, DMSO-d_6) of euglenatide E.

Position (No.)	^{13}C ppm	^{1}H ppm mult. (*J* in Hz)	Position (No.)	^{13}C ppm	^{1}H ppm mult. (*J* in Hz)
Dnv			βAib		
C (1)	172.3		C (1)	176.1	
CH (2)	50.3	4.30, m	CH (2)	38.0	2.64, m
NH (2)		7.64, d (7.8)	CH2 (3)	41.7	2.93, brd (13.0), 3.26, m
CH2 (3)	34.2	1.69, m, 1.87, m	NH (3)		6.94, overlap
CH (4)	67.6	3.43, m	CH3 (4)	17.1	1.02, d (7.1)
OH (4)		4.47, brd (4.7)			
CH2 (5)	66.3	3.24, m, 3.32, m			
OH (5)		4.41, m			
Asn I			DeoxyGra		
C (1)	171.1		C (1)	170.4	
CH (2)	49.4	4.42, m	CH2 (2)	40.9	2.16, dd (13.8, 3.3), 2.47, overlap (9.6)
NH (2)		7.87, d (7.9)			
CH2 (3)	35.6	2.57, dd (16.9, 3.7), 3.01, dd (16.8, 5.0)	CH (3)	45.2	4.08, m (9.6, 6.5, 3.3)
			NH (3)		6.92, overlap
C (4)	173.9		CH2 (4)	44.2	1.32, m
NH2 (4)		7.91 s, 7.39 s	CH (5)	63.3	3.51, m
			OH (5)		4.55, m
			CH2 (6)	43.6	1.30, m, 1.48, m
Asn II			CH (7)	77.8	3.75, m
C (1)	169.7		CH (8)	133.8	5.51, dd (14.6, 7.6)
CH (2)	51.7	4.11. m	CH (9)	131.5	6.19, dd (14.6, 10.2)
NH (2)		8.96, d (6.5)	CH (10)	129.9	6.15, dd (14.5, 10.3)
CH2 (3)	35.0	2.47, dd (15.9, 4.7), 2.87, dd (15.7, 4.0)	CH (11)	133.0	6.24, dd (14.5, 10.3)
			CH (12)	130.3	6.07, dd (15.1, 10.4)
C (4)	172.2		CH (13)	135.3	5.72, dt (15.1, 7.1)
NH2 (4)		7.36, s, 6.88, s	CH2 (14)	32.2	2.06, q (7.0)
			CH2 (15)	28.8	1.35, m
			CH2 (16)	28.5	1.25, m
			CH2 (17)	29.0	1.25, m
			CH2 (18)	31.3	1.25, m
			CH2 (19)	22.1	1.27, m
			CH3 (20)	14.0	0.85, t (6.8)
			CH3 (21)	55.6	3.13, s

Table S5 ^{1}H and ^{13}C NMR data (500 and 125 MHz, DMSO-d_6) for euglenatide C.

Position (No.)	^{13}C ppm	^{1}H ppm mult. (*J* in Hz)	Position (No.)	^{13}C ppm	^{1}H ppm mult. (*J* in Hz)
Dnl			βAib		
C (1)	172.5		C (1)	176.2	
CH (2)	50.5	4.32, m	CH (2)	37.5	2.66, m
NH (2)		7.65, d (7.8)	CH2 (3)	41.7	3.03, brd (11.2), 3.27, m
CH2 (3)	34.2	1.86, m, 1.71, m	NH (3)		7.06, s
CH (4)	70.6	3.18, m	CH3 (4)	16.9	1.02, t (6.2)
OH (4)		4.27, brd (5.9)			
CH (5)	69.9	3.39, m			
OH (5)		4.18, brd (4.9)			
CH3 (6)	19.5	1.02, t (6.2)			
Asn I			Gra		
C (1)	171.1		C (1)	172.3	
CH (2)	49.3	4.45, m	CH (2)	72.9	3.86, dd (9.6, 7.7)
NH (2)		7.76, d (8.5)	OH (2)		5.13, brd (7.7)
CH2 (3)	35.8	2.57, dd (17.0, 3.6), 2.94, dd (16.9, 5.0)	CH (3)	49.1	3.92, m
			NH (3)		6.90 d (9.7)
C (4)	173.4		CH2 (4)	40.3	1.69, m, 1.20, m
NH2 (4)		7.79 s, 7.29 s	CH (5)	63.0	3.47, m
			OH (5)		4.31, m
			CH2 (6)	43.8	1.30, br dd (14.5, 5.2), 1.49, m
Asn II					
C (1)	169.5		CH (7)	77.8	3.73, m
CH (2)	51.6	4.06. m	CH (8)	133.9	5.51, dd (14.6, 7.6)
NH (2)		9.02, d (6.3)	CH (9)	131.3	6.20, dd (14.6, 10.3)
CH2 (3)	35.0	2.86, dd (15.4, 3.9) 2.47, dd (15.5, 4.0)	CH (10)	130.0	6.15, dd (14.6, 10.3)
			CH (11)	132.8	6.24, dd (14.5, 10.4)
C (4)	172.1		CH (12)	130.3	6.07, dd (15.1, 10.4)
NH2 (4)		7.36, s, 6.88, s	CH (13)	135.1	5.72, dt (15.1, 7.1)
			CH2 (14)	32.1	2.06, q (7.1)
			CH2 (15)	28.7	1.35, m
			CH2 (16)	28.52	1.25, m
			CH2 (17)	28.54	1.25, m
			CH2 (18)	31.2	1.24, m
			CH2 (19)	22.0	1.25, m
			CH3 (20)	13.9	0.85, t (6.8)
			CH3 (21)	55.5	3.13, s

Table S6 ^{1}H and ^{13}C NMR data (500 and 125 MHz, DMSO-d_6) of euglenatide A.

Position (No.)	^{13}C ppm	^{1}H ppm mult. (*J* in Hz)	Position (No.)	^{13}C ppm	^{1}H ppm mult. (*J* in Hz)
Dnv I			βAib		
C (1)	172.6		C (1)	176.2	
CH (2)	50.6	4.28, m	CH (2)	37.7	2.69, m
NH (2)		7.73, d (7.7)	CH2 (3)	41.8	3.06, brd (12.9), 3.27, m
CH2 (3)	34.5	1.83, m, 1.71, m	NH (3)		7.08, brd (12.7)
CH (4)	67.6	3.42, m	CH3 (4)	17.0	1.07, d (6.9)
OH (4)		4.44, m			
CH2 (5)	66.3	3.22, m, 3.29, m			
OH (5)		4.34, m			
Asn I			Gra		
C (1)	171.2		C (1)	172.3	
CH (2)	49.3	4.45, m	CH (2)	73.0	3.87, d (9.6, 7.5)
NH (2)		7.78, s	OH (2)		5.17, brd (7.5)
CH2 (3)	35.8	2.57, dd (16.5, 3.4), 2.94, dd (16.8, 4.8)	CH (3)	49.2	3.92, m
			NH (3)		6.92 d (9.6)
C (4)	173.6		CH2 (4)	40.4	1.69, m, 1.20, m
NH2 (4)		7.79 s, 7.29 s	CH (5)	63.1	3.47, m
			OH (5)		4.31, m
			CH2 (6)	43.9	1.31, br dd (15.0, 4.8), 1.49, m
Dnv II					
C (1)	170.7		CH (7)	77.9	3.73, m
CH (2)	51.6	3.81, m	CH (8)	134.0	5.51, dd (14.7, 7.9)
NH (2)		8.85, d (5.7)	CH (9)	131.4	6.20, dd (14.6, 10.3)
CH2 (3)	32.3	1.89, m	CH (10)	130.1	6.15, dd (14.0, 10.6)
CH (4)	68.1	3.41, m	CH (11)	133.0	6.24, dd (14.0, 10.6)
OH (4)		4.42, m	CH (12)	130.4	6.07, dd (14.0, 10.6)
CH2 (5)	66.3	3.22, m, 3.29, m	CH (13)	135.2	5.72, dt (14.1, 7.1)
OH (5)		4.55, m	CH2 (14)	32.2	2.06, q (7.0)
			CH2 (15)	28.8	1.34, m
			CH2 (16)	28.6	1.25, m
			CH2 (17)	28.6	1.25, m
			CH2 (18)	31.3	1.24, m
			CH2 (19)	22.1	1.25, m
			CH3 (20)	14.0	0.85, t (6.9)
			CH3 (21)	55.6	3.13, s

Table S7 ^{1}H and ^{13}C NMR data (500 and 125 MHz, DMSO-d_6) for euglenatide D.

Position (No.)	^{13}C ppm	^{1}H ppm mult. (*J* in Hz)	Position (No.)	^{13}C ppm	^{1}H ppm mult. (*J* in Hz)
Dnv I			βAib		
C (1)	172.3		C (1)	175.9	
CH (2)	50.4	4.26, m	CH (2)	38.2	2.66, m
NH (2)		7.71, d (7.5)	CH2 (3)	41.9	2.92, brd (12.4), 3.24, m
CH2 (3)	34.2	1.85, m, 1.69, m	NH (3)		6.93, overlap
CH (4)	67.7	3.42, m	CH3 (4)	17.4	1.07, br d (7.1)
OH (4)		4.44, m			
CH2 (5)	66.3	3.22, m, 3.29, m			
OH (5)		4.34, m			
Asn I			DeoxyGra		
C (1)	171.2		C (1)	170.4	
CH (2)	49.4	4.42, m	CH2 (2)	41.0	2.16, dd (12.8, 3.1), 2.47, overlap (9.6)
NH (2)		7.87, d (8.2)			
CH2 (3)	35.6	2.58, dd (16.7, 3.2), 2.99, dd (16.9, 4.15)	CH (3)	45.2	4.08, m (9.6, 6.5, 3.3)
			NH (3)		6.92, overlap
C (4)	174.1		CH2 (4)	44.2	1.32, m
NH2 (4)		7.93 s, 7.43 s	CH (5)	63.4	3.51, m
			OH (5)		4.55, m
			CH2 (6)	43.6	1.30, m, 1.49, m,
Dnv II			CH (7)	77.8	3.75, m
C (1)	170.7		CH (8)	133.8	5.51, dd (14.7, 7.7)
CH (2)	51.6	3.85, m	CH (9)	131.5	6.20, dd (14.5, 10.5)
NH (2)		8.85, d (5.3)	CH (10)	130.0	6.15, dd (14.7, 10.6)
CH2 (3)	32.2	1.89, m	CH (11)	133.1	6.24, dd (14.5, 10.5)
CH (4)	68.0	3.41, m	CH (12)	130.4	6.07, dd (15.0, 10.5)
OH (4)		4.42, m	CH (13)	135.3	5.72, dt (15.1, 7.7)
CH2 (5)	66.3	3.22, m, 3.29, m	CH2 (14)	32.2	2.06, q (7.0)
OH (5)		4.57, m	CH2 (15)	28.8	1.35, m
			CH2 (16)	28.6	1.25, m
			CH2 (17)	29.0	1.25, m
			CH2 (18)	31.3	1.23, m
			CH2 (19)	22.1	1.26, m
			CH3 (20)	14.0	0.85, t (6.8)
			CH3 (21)	55.7	3.13, s

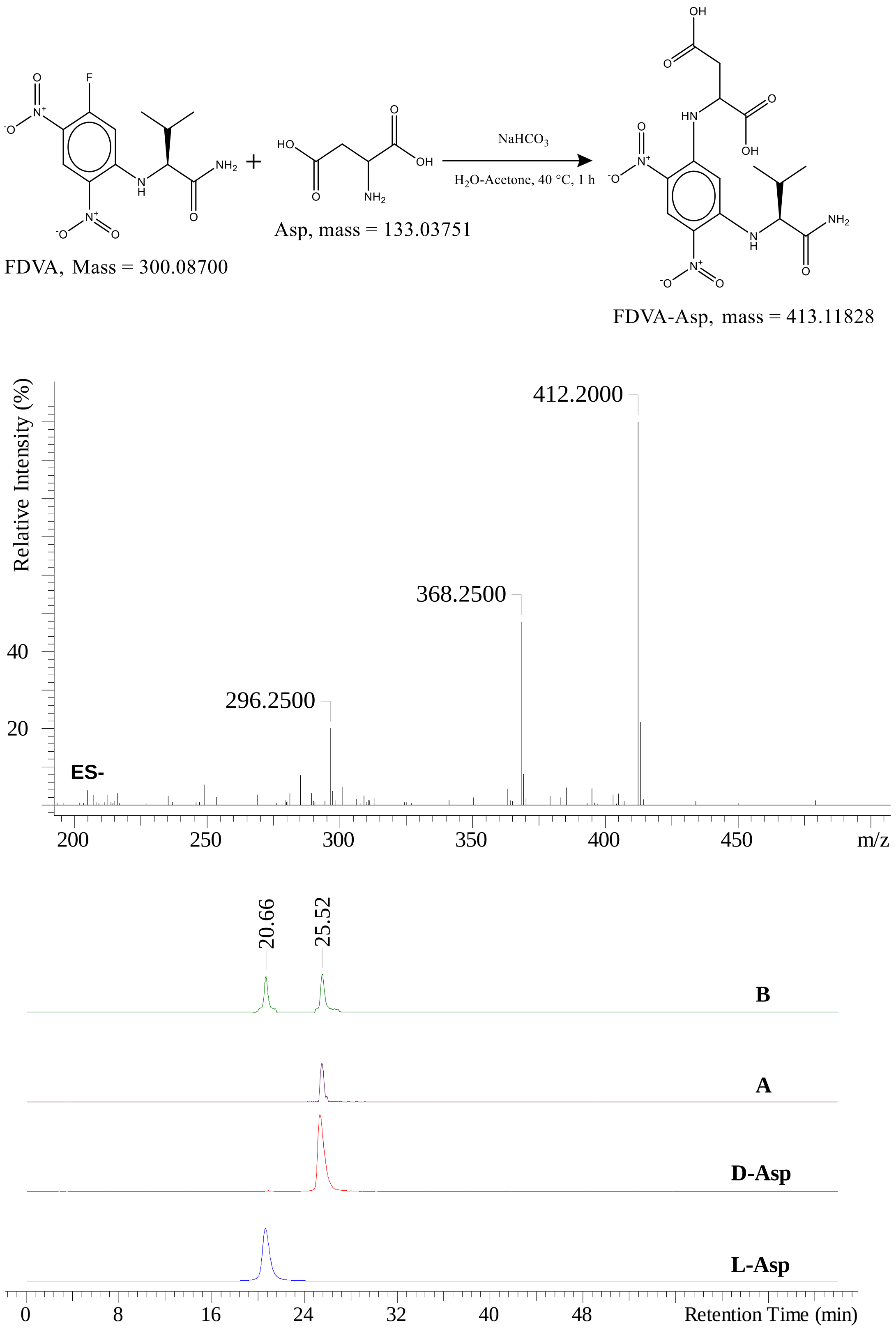

Figure S20 Top: Reaction of Asp with Marfey's reagent. Bottom: extracted negative ion chromatograms of the *m/z* 412 in derivatised Asp standards and euglenatides A and B hydrolysates.

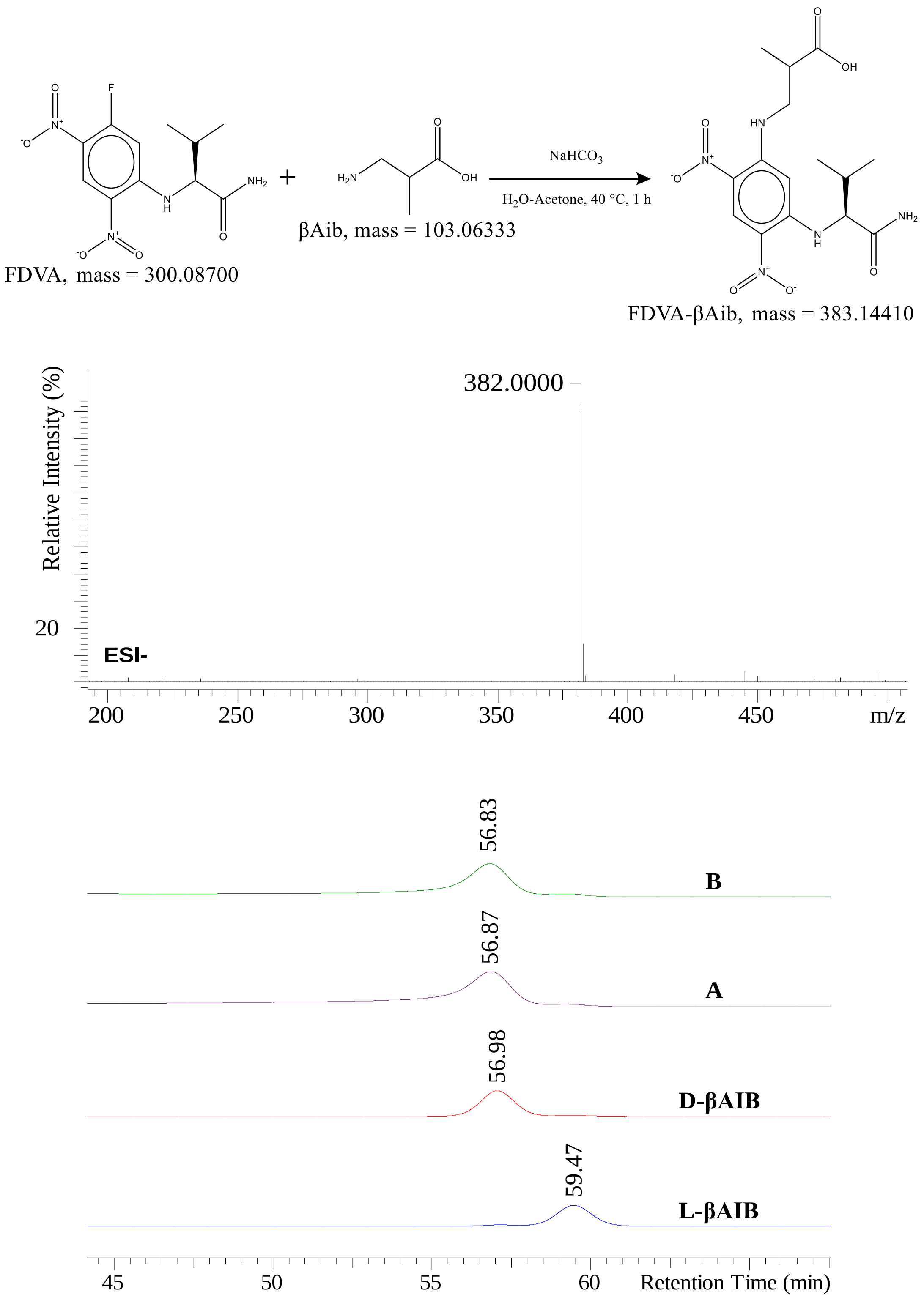

Figure S21 Top: Reaction of βAib with Marfey's reagent. Bottom: extracted negative ion chromatograms of the *m/z* 382 in derivatised βAib standards and euglenatides A and B hydrolysates.

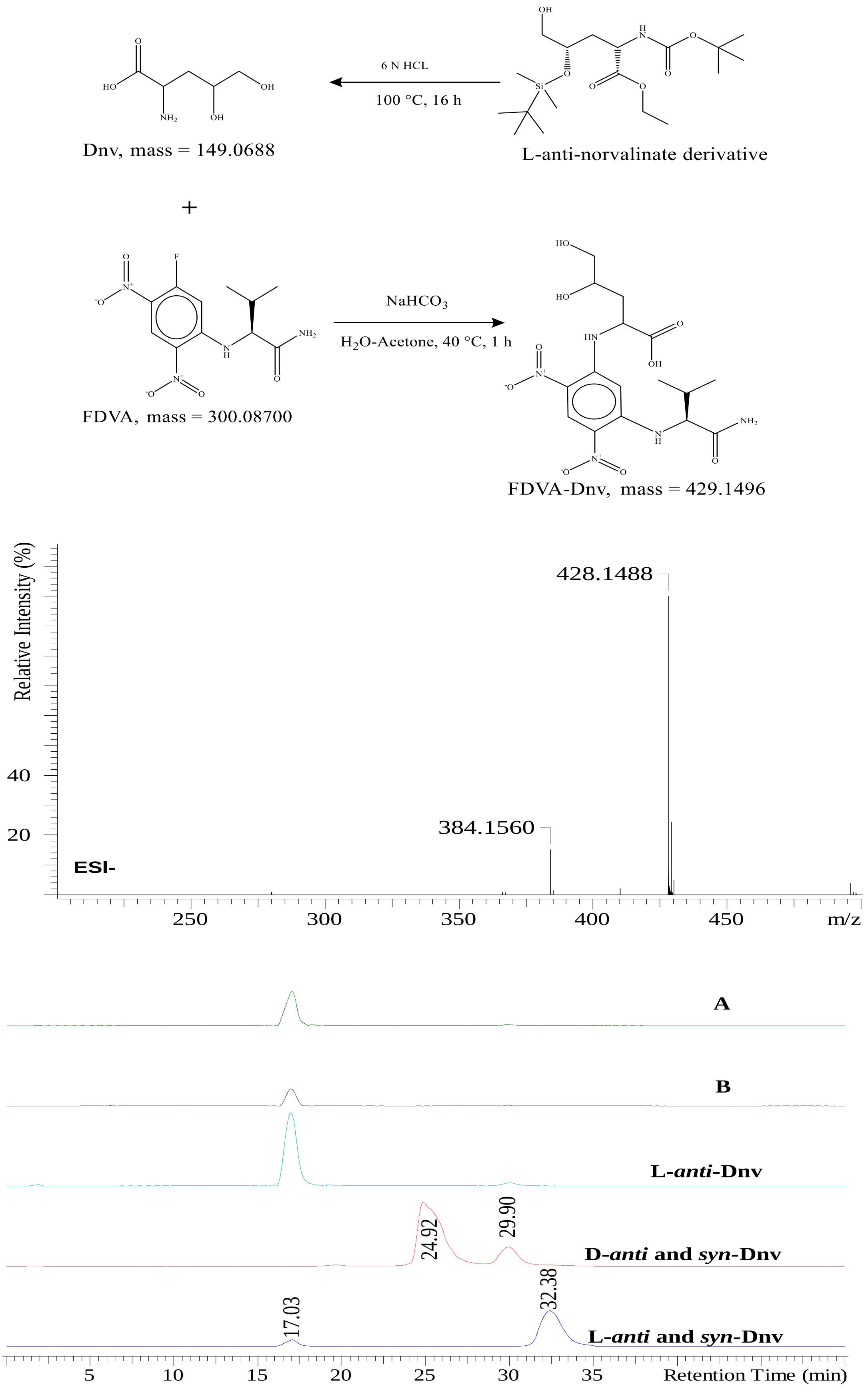

Figure S22 Top: hydrolysis of the protected Dnv derivative and reaction with Marfey's reagent. Bottom: extracted ion chromatograms and spectra of the *m/z* 428 in derivatised Dnv standards and euglenatides A and B hydrolysates.

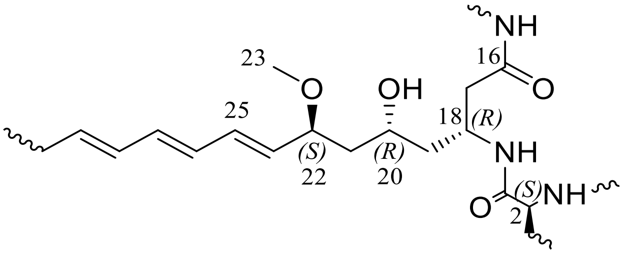

Table S8 Comparison of ^{1}H NMR chemical shifts in the triene side chain between euglenatides and nemamide A.

Proton position	nemamide A	euglenatide E	euglenatide B	$\Delta\delta$
C17	2.18, brd (*J* 17a,17b = 14.5)<br>2.47, overlap (*J* 17b,18 = 9.8)	2.16, dd (13.8, 3.3),<br>2.47, overlap	3.86, dd (9.6, 7.7)	-0.02, 0
C18	4.08, m (*J* 18,19 =6.7)	4.08, m	3.92, m	0
C19	1.32, m (*J* 19,20 = 6.7)	1.31, m	1.20, m ,1.69 m	-0.01
C20	3.51, m (*J* 20,21a = 8.2)	3.51, m	3.47, m	0
C21a	1.30, m (*J* 21a,21b =14.5)	1.30, m	1.30, m	0
C21b	1.48, m (*J* 21b,22 = 9.8)	1.48, m	1.49, m	0
C22	3.76, m (*J* 22,24 = 7.5)	3.75, m	3.73, m	-0.01
C23	3.13, s	3.13, s	3.13, s	0
C24	5.51, dd (*J* 24,25 = 15.4)	5.51, dd (*J* 14.6, 7.6)	5.51, dd (*J* 14.6, 7.6)	0
C25	6.19, dd (*J* 25,26 = 11.2)	6.19, dd (*J* 14.6, 10.2)	6.20, dd (*J* 14.6, 10.3)	0

Assignment of C17 stereochemistry in euglenatide B was based on comparison of dihedral angles and coupling constant with nemamide A.

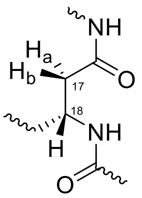

nemamide A

H17$_b$ - H18 coupling = large, 9.8 Hz (dihedral angle close to zero)
H17$_a$ - H18 coupling = small, not reported (dihedral angle close to 60°)

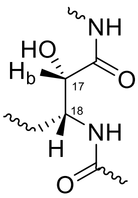

euglenatide B

H17$_b$ - H18 coupling = large, 9.6 Hz (dihedral angle close to zero)

Table S9 Comparison of ^{13}C NMR chemical shifts in the triene side chain between euglenatides and nemamide A.

Carbon position	nemamide A	euglenatide E	euglenatide B	Δδ
C16	170.3	170.4	172.3	0.1
C17	40.6	40.9	72.9	0.3
C18	44.9	45.2	49.1	0.3
C19	43.7	44.2	40.3	0.5
C20	63.3	63.3	63.0	0
C21	43.5	43.6	43.8	0.1
C22	77.6	77.8	77.8	0.2
C23	55.5	55.6	55.5	0.1
C24	133.7	133.8	133.9	0.1
C25	131.3	131.5	131.3	0.2

Table S10 Comparison of NOESY correlations and *J* coupling between protons at C2, N2, N18 and C17 in the triene side chain of euglenatides and reported data for nemamide A.

	C2H	N2H	N18H	C17H	C18H	^{1}H NOESY correlations	J coupling (Hz)
euglenatide E	4.30	7.64	6.93	Ha, 2.16<br>Hb, 2.47	4.08	C2H-N2H<br>C2H-N18H<br>N2H-N18H<br>C17Hb-N18H	C17Hb-C18H, 9.6<br>C17Ha-C18H, 3.3
euglenatide B	4.32	7.65	6.90	CH, 3.86<br>OH, 5.13	3.92	C2H-N2H<br>C2H-N18H<br>N2H-N18H<br>C17H-N18H	C17H-C18H, 9.6<br>C17H-O17H, 7.7
nemamide A	4.51	7.46	7.05	Ha, 2.18<br>Hb, 2.47	4.08	C2H-N18H<br>N2H-N18H<br>C17Hb-N18H	C17Hb-C18H, 9.8

Table S11 Comparison of NOESY correlations and *J* coupling between protons at C17, C18, C20, C21, C22 and C24 in the triene side chain of euglenatides and reported data for nemamide A.

	C17H	C18H	C20H	C21H	C22H	C24H	^{1}H NOESY correlations	J coupling (Hz)
euglenatide E	Ha, 2.16<br>Hb, 2.47	4.08	3.51	Ha, 1.30<br>Hb, 1.48	3.75	5.51	C17Ha -C20H<br>CH18-C20H<br>C18H -C21Hb<br><br>C20H- C22H<br>C21Ha-C24H<br>C21Hb-C24H	C21Hb-C20H, 5.1<br>C21Ha-C20H, 14.5<br><br><br>C21Hb-C22H, 8.9<br>C21Ha-C22H, 3.54
euglenatide B	CH, 3.86<br>OH, 5.13	3.92	3.47	Ha, 1.30<br>Hb, 1.49	3.73	5.51	C17H -C20H<br>CH18-C20H<br>C18H -C21Hb<br><br>C20H- C22H<br>C21Ha-C24H<br>C21Hb-C24H	C21Hb-C20H, 5.1<br>C21Ha-C20H, 14.5<br><br><br>C21Hb-C22H, 8.9<br>C21Ha-C22H, 3.54
nemamide A	Ha, 2.18<br>Hb, 2.47	4.08	4.08	Ha, 1.30<br>Hb, 1.48	3.76	5.51	C17Ha -C20H<br>CH18-C20H<br>C18H -C21Hb<br><br>C20H- C22H<br>C21Ha-C24H<br>C21Hb-C24H	C21Ha-C20H, 8.2<br><br><br><br>C21Hb-C22H, 9.8

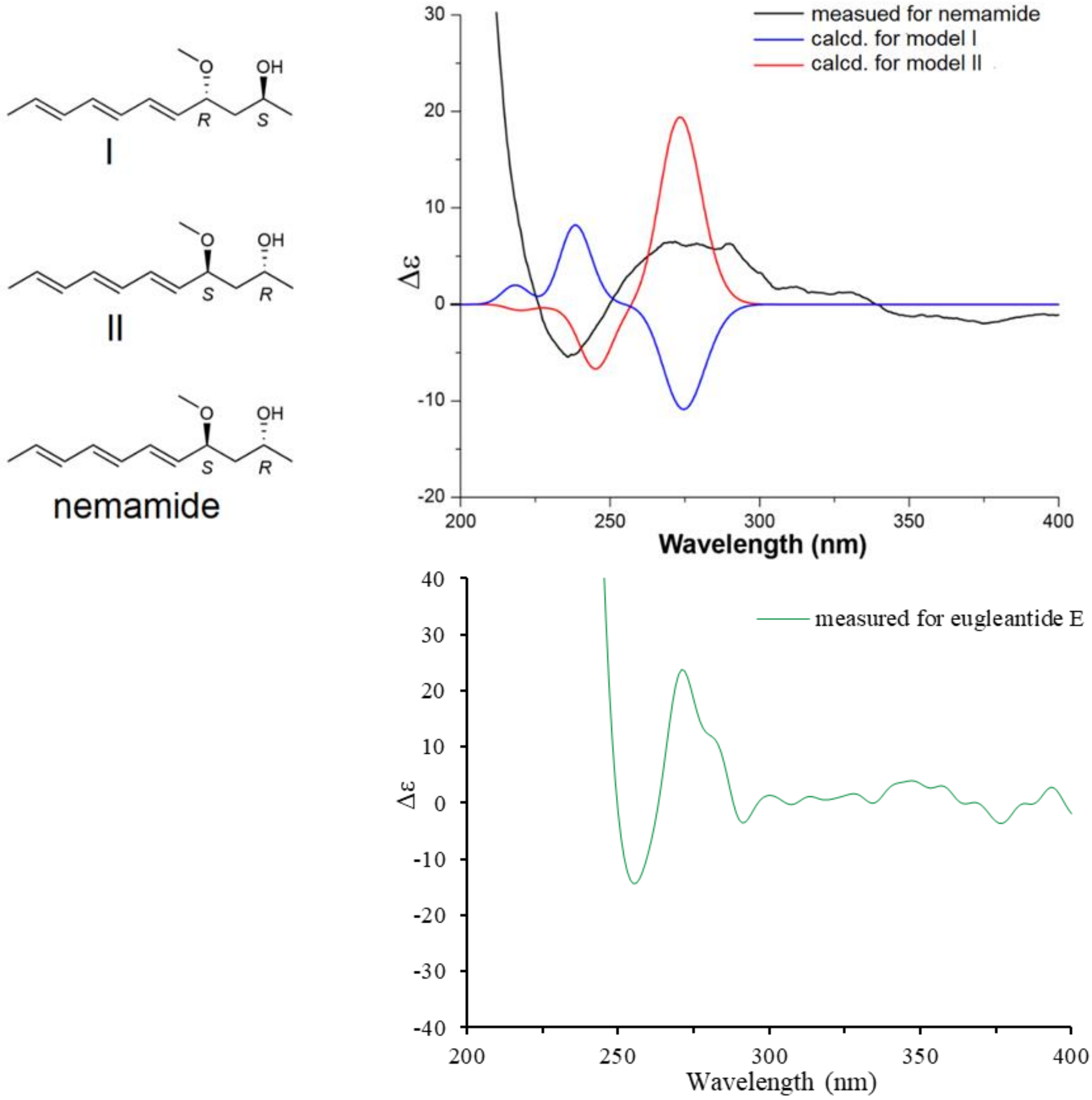

Figure S23 Comparison of CD spectra between euglenatide E and those reported for nemamide A and triene diastereomers.[9]

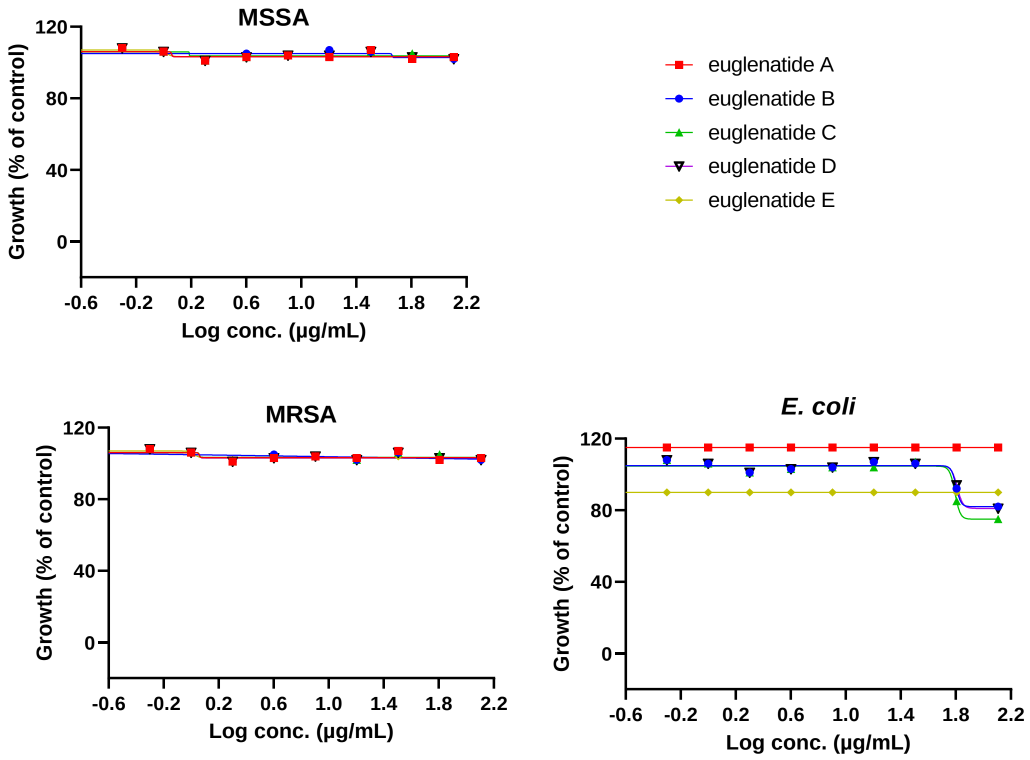

Figure S24 Dose–response curves of bacterial strains (MSSA, MRSA and E. coli) incubated with euglenatides A, B, C, D and E.

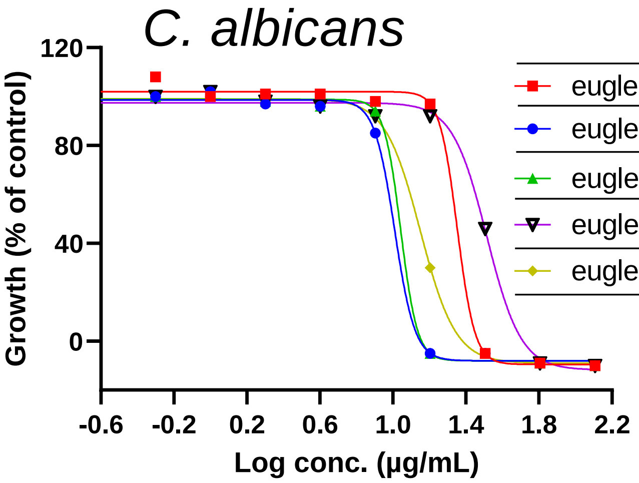

	IC_{50} (µg/mL)	IC_{50} (µM)
euglenatide A	22.5	27.2
euglenatide B	10.2	12.6
euglenatide C	11.0	13.4
euglenatide D	32.7	40.3
euglenatide E	14.1	17.8

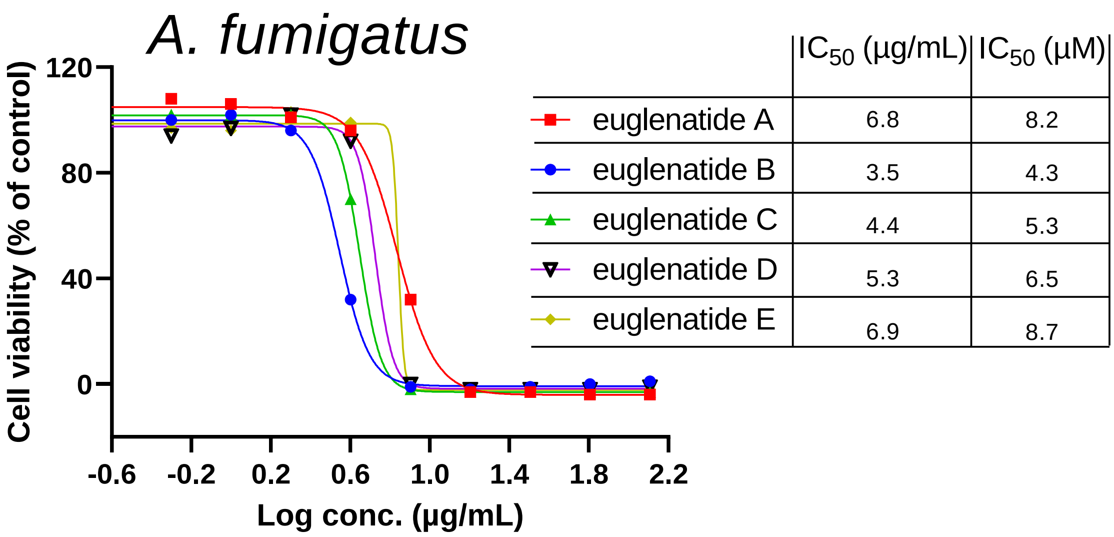

	IC_{50} (µg/mL)	IC_{50} (µM)
euglenatide A	6.8	8.2
euglenatide B	3.5	4.3
euglenatide C	4.4	5.3
euglenatide D	5.3	6.5
euglenatide E	6.9	8.7

Figure S25 Dose–response curves and corresponding IC_{50} values of *C. albicans* and *A. fumigatus* incubated with euglenatides A, B, C, D and E.

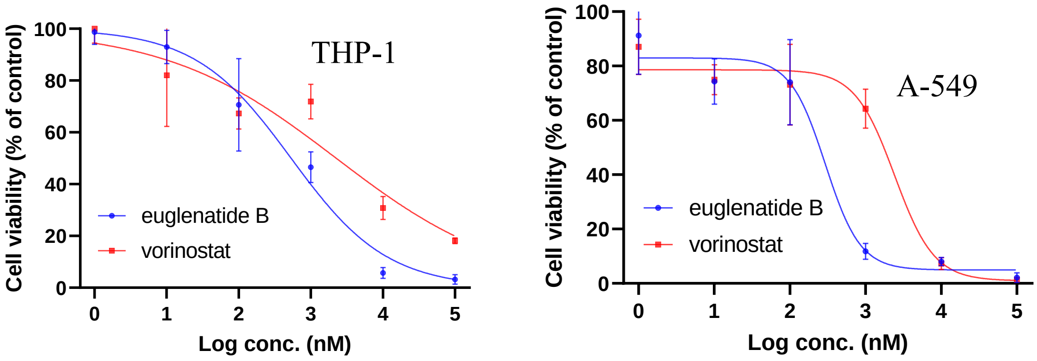

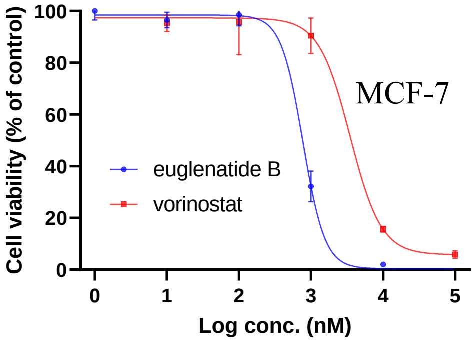

Compound	IC50(μM) ± SEM against THP-1	IC50(μM) ± SEM against A-549	IC50(μM) ± SEM against MCF-7
euglenatide B	0.53 ± 0.16	0.77 ± 0.03	0.29 ± 0.02
vorinostat	2.3 ± 0.50	3.5 ± 0.31	2.4 ± 0.60

Figure S26 Dose–response curves of THP-1, MCF-7 and A-549 cell lines incubated with euglenatide B or vorinostat (positive control). Data points represent the average ± standard error of three replicates.

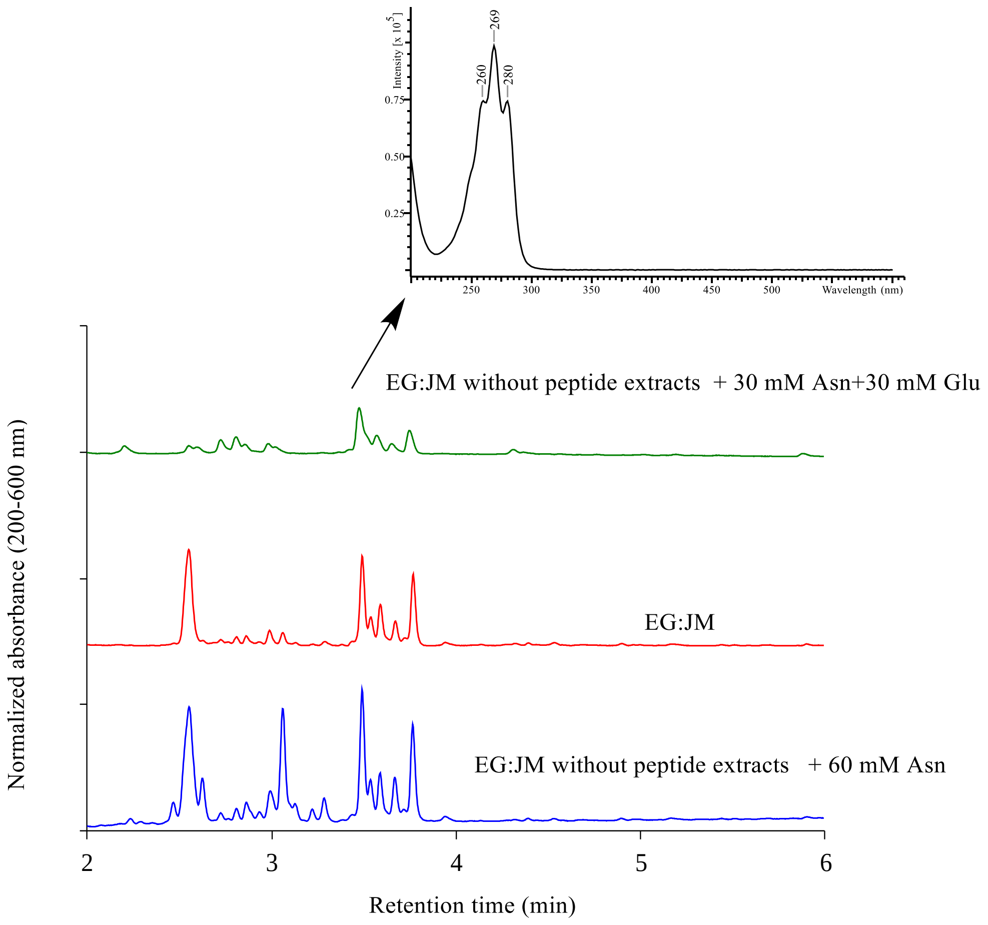

Figure S27 Comparative total absorbance (TAC, 200-600 nm) of *E sanguinea* cultivated in EG:JM medium without peptide extracts + 60 mM Asn, EG:JM medium and EG:JM medium without peptide extracts + 30 mM Asn + 30 mM Glu.

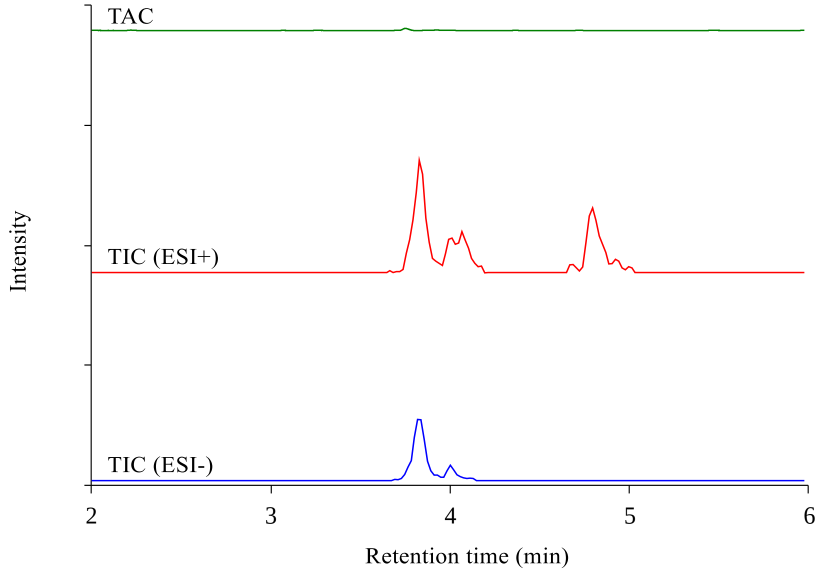

Figure S28 Comparative total absorbance (200-600 nm) and total ion chromatograms of *E. mutabilis* cultivated in EG:JM medium.

References

[1] C. N. Campbell, Scottish Association for Marine Science, “Elimination of bacteria from microalgal culture using antibiotics,” https://www.ccap.ac.uk/documents/Antibiotic_treatment.pdf**.**
[2] E. C. O’Neill, M. Trick, L. Hill, M. Rejzek, R. G. Dusi, C. J. Hamilton, P. V. Zimba, B. Henrissat, R. A. Field, *Mol. Biosyst.* **2015**, *11*, 2808–2820.
[3] J. Martín, T. da S Sousa, G. Crespo, S. Palomo, I. González, J. R. Tormo, M. de la Cruz, M. Anderson, R. T. Hill, F. Vicente, et al., *Mar. Drugs* **2013**, *11*, 387–398.
[4] M. C. Monteiro, M. de la Cruz, J. Cantizani, C. Moreno, J. R. Tormo, E. Mellado, J. R. De Lucas, F. Asensio, V. Valiante, A. A. Brakhage, et al., *J. Biomol. Screen.* **2012**, *17*, 542–549.
[5] C. Audoin, D. Bonhomme, J. Ivanisevic, M. Cruz, B. Cautain, M. Monteiro, F. Reyes, L. Rios, T. Perez, O. Thomas, *Mar. Drugs* **2013**, *11*, 1477–1489.
[6] M. Wang, J. J. Carver, V. V Phelan, L. M. Sanchez, N. Garg, Y. Peng, D.-T. D. D. Nguyen, J. Watrous, C. A. Kapono, T. Luzzatto-Knaan, et al., *Nat. Biotechnol.* **2016**, *34*, 828–837.
[7] T. H. Eyles, N. M. Vior, A. W. Truman, *ACS Synth. Biol.* **2018**, *7*, 1211–1218.
[8] P. Shannon, *Genome Res.* **2003**, *13*, 2498–2504.
[9] Q. Shou, L. Feng, Y. Long, J. Han, J. K. Nunnery, D. H. Powell, R. A. Butcher, *Nat. Chem. Biol.* **2016**, *12*, 770–772.
[10] R. G. Soengas, J. C. Estévez, R. J. Estévez, *Tetrahedron: Asymmetry* **2003**, *14*, 3955–3963.
[11] J. Ariza, M. Díaz, J. Font, R. M. Ortuño, *Tetrahedron* **1993**, *49*, 1315–1326.
[12] K. Isono, K. Asahi, S. Suzuki, *J. Am. Chem. Soc.* **1969**, *91*, 7490–7505.
